# Hybrid Uracil Derivatives with Caffeine and Gramine Obtained via Click Chemistry as Potential Antioxidants and Inhibitors of Plant Pathogens

**DOI:** 10.3390/molecules30132714

**Published:** 2025-06-24

**Authors:** Milda Szlaużys, Kamil Ostrowski, Damian Nowak, Wiesław Prukała, Justyna Starzyk, Beata Jasiewicz, Lucyna Mrówczyńska

**Affiliations:** 1 Department of Bioactive Products, Faculty of Chemistry, Adam Mickiewicz University, Uniwersytetu Poznańskiego 8, 61-614 Poznań, Poland; milda.szlauzys@amu.edu.pl (M.S.); kamil.ostrowski@amu.edu.pl (K.O.); beata.jasiewicz@amu.edu.pl (B.J.); 2 Department of Quantum Chemistry, Faculty of Chemistry, Adam Mickiewicz University, Uniwersytetu Poznańskiego 8, 61-614 Poznań, Poland; damian.nowak@amu.edu.pl; 3 Department of Natural Products, Faculty of Chemistry, Adam Mickiewicz University, Uniwersytetu Poznańskiego 8, 61-614 Poznań, Poland; wprukala@amu.edu.pl; 4 Department of Soil Science and Microbiology, Faculty of Agronomy, Horticulture, and Bioengineering, University of Life Science, Szydłowska 50, 60-656 Poznań, Poland; jstarzyk@up.poznan.pl; 5 Department of Cell Biology, Faculty of Biology, Adam Mickiewicz University, Uniwersytetu Poznańskiego 6, 61-614 Poznań, Poland

**Keywords:** uracil derivatives, gramine, caffeine, 1,2,3-triazole, click chemistry, human erythrocytes, antioxidant activity, oxidative stress, antifungal properties

## Abstract

A series of novel hybrid uracil derivatives incorporating the natural alkaloids caffeine or gramine, linked via 1,2,3-triazole ring, were synthetized using click chemistry. The structures of the obtained compounds were confirmed by spectroscopic methods, including ^1^H NMR, ^13^C NMR, FT-IR, and mass spectrometry. The biological activity of hybrids was evaluated in vitro, including assessments of hemolytic activity, antioxidant potential, antifungal efficacy, and antibacterial activity. Additionally, molecular docking studies were conducted in silico for the most active antioxidant candidate. The results revealed that the hemocompatibility of the derivatives was structure-dependent. While caffeine-containing hybrids exhibited moderate-to-low cytoprotective activity under oxidative stress conditions, those incorporating gramine showed significantly higher potency. A plausible molecular mechanism underlying their cytoprotective activity is proposed. Several compounds also inhibited the growth of the plant pathogens *Fusarium culmorum* and *Botrytis cinerea*. The promising antioxidant and antifungal properties of selected uracil–alkaloid hybrids highlight their potential as multifunctional bioactive compounds for managing oxidative stress and controlling plant pathogens. Furthermore, the finding demonstrates the effectiveness of click chemistry as a versatile tool for the synthesis of bioactive heterocyclic compounds.

## 1. Introduction

Nitrogen heterocycles serve as important frameworks in drug design and play a pivotal role in pharmaceutical development [1]. Among them, uracil and thiouracil derivatives are notable for their diverse biological activities, including antiviral and anticancer [2]. For example, 6-methyl-2-thiouracil exhibits bacteriostatic properties [3], while propylthiouracil is used to treat Graves’ disease [4]. Additionally, 5-fluorouracil is a well-established anticancer drug used in the treatment of colon and breast cancers [2]. The 2-thiouracil scaffold is a promising structural motif for antioxidant agents and may serve as a lead structure for further optimization [5].

Alkaloids constitute a large group of heterocyclic compounds and are an important source of bioactive compounds. Notable examples include caffeine and gramine, which exhibit diverse biological activities. The structural modification of caffeine and gramine may yield derivatives with antimicrobial, anticancer, and antiviral properties [6,7,8]. Moreover, our previous studies have demonstrated that selected caffeine and gramine derivatives exhibit significant antioxidant activity [9,10,11,12]. Antioxidants protect cells from damage induced by reactive oxygen species (ROS). An imbalance between ROS generation and antioxidant defenses leads to oxidative stress, a key factor in the pathogenesis of numerous diseases, including cancer, diabetes, neurodegenerative disorders, and atherosclerosis [13].

Human red blood cells (RBCs) are widely used to assess the cytotoxic and antioxidant effects of bioactive compounds [9,10,11,12]. Their simple structure, lacking organelles and consisting mainly of erythroplasm, makes RBCs ideal for studying compound-cell membrane interactions. Compounds that maintain RBC membrane integrity and do not increase its permeability are considered hemocompatible and suitable for blood-contacting applications [14]. Additionally, RBCs comprise about 70% of all human cells; they serve as a general model for the cell membrane [15]. Under oxidative stress, RBCs undergo cell membrane damage and hemolysis, leading to the release of hemoglobin (Hb) [9,10,11,12]. The oxidation of Hb (Fe^2+^) to methemoglobin (Fe^2+^), which cannot bind oxygen, serves as a marker of ROS-induced injury and impaired RBC function [16]. Antioxidants protect RBCs by limiting oxidative damage and preserving Hb activity, partly through iron chelation [17]. Antioxidant potential is evaluated using RBC-based and RBC-free in vitro assays combined with in silico molecular docking, providing insight into cytoprotective mechanisms at the molecular level [14,18].

In addition to the antioxidant properties, certain derivatives of caffeine and gramine exhibit significant antimicrobial and antifungal activities [10,11,12,19]. From an environmental perspective, the development of non-toxic agents with antibacterial activity and for controlling plant pathogens is increasingly contributing to reduced ecological impact and the preservation of ecosystems. In this context, the present study also investigates the antibacterial and antifungal potential of newly synthesized hybrid compounds. These hybrids, formed by combining at least two bioactive scaffolds, may yield molecules with enhanced or synergistic biological properties.

This study aimed to design, synthesize, and characterize a novel series of 2-thiouracil-based hybrids incorporating caffeine or gramine via a 1,2,3-triazole ring. Selected biological activities, focusing on the impact of structural features, specifically the uracil substituent and alkaloid type, were evaluated. The findings support the development of multifunctional, nature-inspired agents for managing oxidative stress and plant pathogens.

## 2. Results and Discussion

### 2.1. Synthesis and Spectroscopy

**Scheme 1:**
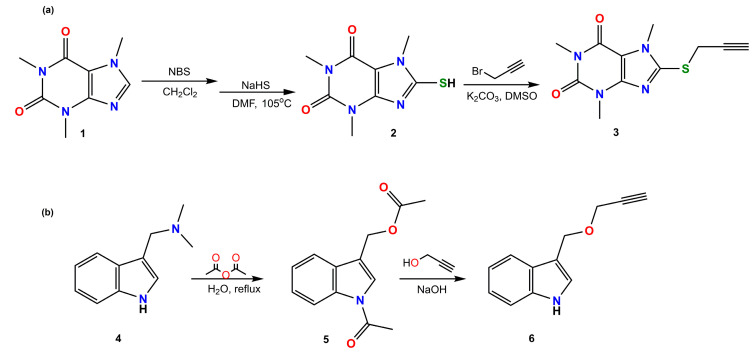
Synthesis of (**a**) compound **3** and (**b**) compound **6**.

The synthesis of 8-propargyl caffeine (compound **3**) involved three steps. First, caffeine (compound **1**) was converted to 8-bromocaffeine due to [20]. Then, it reacted with sodium hydrosulfide (NaHS) in dimethylformamide (DMF) to yield 8-thiocaffeine (compound **2**) due to [21]. Compound **2** was subsequently treated with propargyl bromide to produce compound **3** (see Scheme 1). Compound **6** was synthesized by reacting propargyl alcohol with *N,O*-diacetyl-indole-3-carbinol (compound **5**) [12]. Derivative **5** was synthesized by the reaction of gramine with acetic anhydride [12] (Scheme 1).

**Scheme 2:**
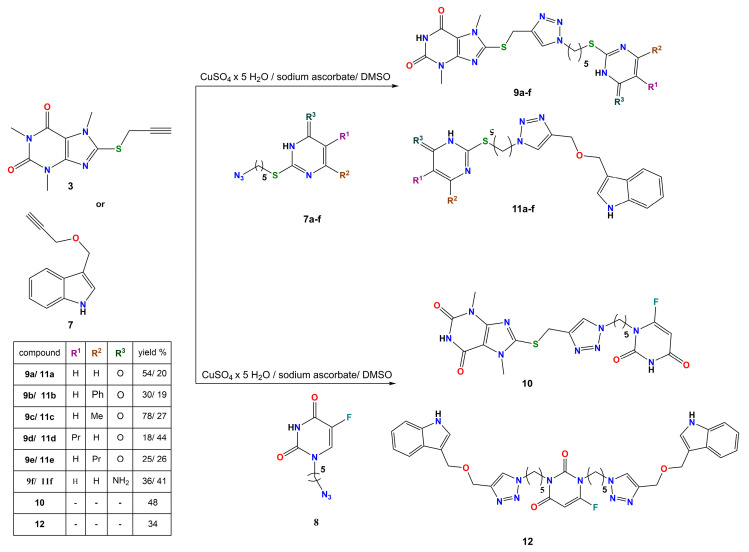
Synthesis of novel uracil–caffeine (ratio 1:1) and uracil–gramine (ratio 1:1) hybrid derivatives.

Compounds **7a–f** and **8** were synthesized in a two-step reaction. In the first step, the corresponding uracil derivatives were mixed with dibromopentane to form bromopentane–uracil derivatives. This was followed by a reaction with sodium azide to give azidopentane analogs. Derivatives **9a–f**, **10**, **11a–f**, and **12** were synthesized using Cu(I)-catalyzed alkyne/azide cycloadditions (Scheme 2). The yields of these reactions varied significantly, ranging from 18% to 78%, with an average yield of about 40%.

The reaction of fluorouracil azide with compound **6** produced compound **12**, which features two indole moieties and two triazole rings linked by an alkyl group. Conversely, the reaction of the same azide with compound **3**, regardless of the molar ratio of the substrates (1:1, 2:1, or 3:1), resulted in a derivative with one triazole ring and one purine group, compound **10**.

The newly obtained compounds were spectrally characterized by ^1^H NMR, ^13^C NMR, FT-IR, and ESI-MS spectrometry. Spectra of the investigated compounds (Figures S2a–S15d) and spectral descriptions (pages S3–S7) are provided in the Supplementary Materials.

### 2.2. Hemolytic Activity

Hemolysis induced by bioactive compounds is a process in which the membrane of red blood cells (RBCs) loses its integrity due to interaction between these compounds and cell membrane components, leading to the release of hemoglobin into the extracellular medium [22]. Therefore, the in vitro evaluation of hemolytic activity is a key step in assessing the hemocompatibility of novel compounds as potential blood-contacting applications in vivo [10]. Hemolysis typically occurs when bioactive molecules incorporate into the lipid bilayer of the RBCs’ membrane, increasing its permeability to ions and ultimately compromising membrane stability. In accordance with our previous studies [10], compounds that induced less than 10% hemolysis are classified as hemocompatible.

**Figure 1:**
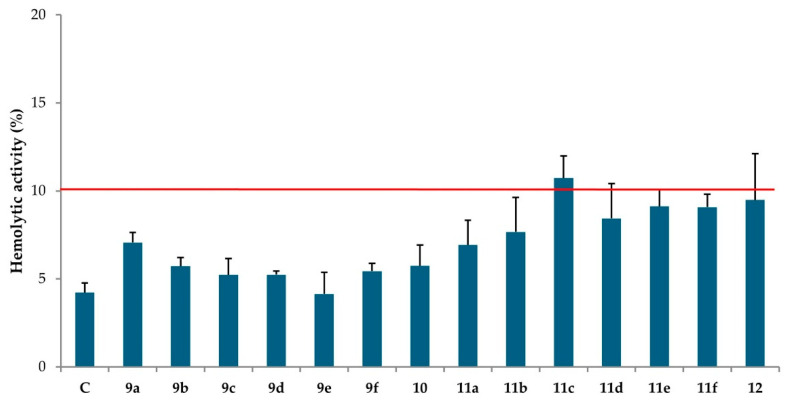
Hemolytic activity (%) of novel compounds at the concentration of 0.01 mg/mL (mean values ± standard deviation, n = 9) after 60 min at 37 °C. C: control, PBS buffer. **9a–10**: hybrid uracil-caffeine derivatives; **11a–12**: hybrid uracil–gramine derivatives. The red line indicates the hemolysis threshold of 10%.

As shown in Figure 1, caffeine derivatives (**9a–10**) exhibit lower hemolytic activity compared to gramine-based compounds (**11a–12**).

Among the caffeine derivatives, compound **9a**, containing an unsubstituted uracil ring, showed the highest hemolytic activity (7.06% ± 0.58). The introduction of substituents into the uracil ring resulted in a reduction in this hemolytic effect, with compound **9e**, containing a n-propyl substituent at the C-6 position of the pyrimidine ring, exhibiting the lowest activity (4.14% ± 1.23). These findings suggest that structural modification within the uracil ring affects the membrane-incorporating properties of the derivatives. In contrast, the presence of a substituent in the pyrimidine ring appears to enhance the hemolytic activity of gramine derivatives. The highest activity was observed for compound **11c** (10.72% ± 1.26), with a methyl group at the C-6 position of the uracil ring.

Conversely, the lowest hemolytic activity among gramine derivatives was recorded for compound **11a** (6.93% ± 1.40), lacking any substituents in the uracil ring. In our previous work, four gramine–uracil derivatives without a triazole ring were synthesized, exhibiting low hemolytic activity in the range of 4.13–4.89% [11]. In the present study, gramine–uracil derivatives containing a triazole ring (**11b**, **11d**, **11e**, and **12**) displayed higher hemolytic activity, ranging from 7.66% to 9.48%. Similarly, the hemolytic activity value of the gramine conjugate with 5-fluorouracil was 4.13% ± 1.13 [23]; however, the conjugate containing a triazole ring (compound **12**) exhibits approximately twice this value. These results indicate that the incorporation of a triazole ring into the molecule enhances the hemolytic potential of these derivatives.

Taken together, these findings demonstrate that the hemolytic activity of compounds is influenced not only by the nature and position of substituents but also by the type of alkaloid present in the molecule.

### 2.3. Physicochemical Data—ADME

ADME (absorption, distribution, metabolism, excretion) analysis is a fundamental tool in drug discovery, offering critical insight into the pharmacokinetic behavior of compounds. Such assessment helps estimate bioavailability, toxicity, and drug likeness, thereby supporting the identification of promising candidates for further development [14].

As shown in Table 1, the majority of gramine derivatives comply with Lipinski’s rule of five, indicating favorable drug likeness. An exception is compound **12**, which exceeds the recommended lipophilicity threshold. In contrast, all caffeine-based derivatives violate two Lipinski criteria—molecular weight and total polar surface area—suggesting reduced drug likeness and potentially limited oral bioavailability.

According to solubility prediction (Table S1, Supplementary Materials), all newly synthesized compounds, except for caffeine derivative **9f**, are classified as poorly water-soluble. As higher water solubility is generally associated with improved bioavailability, this parameter is particularly important in the design of orally administered agents [24].

Remarkably, ADME parameters and Lipinski’s rule compliance do not show a clear correlation with the hemolytic activity of the derivatives (Figure 1). Although all tested compounds exhibited relatively similar hemolytic activity, ranging from 4.22% to 10.72%, gramine-based derivatives generally demonstrated slightly higher activity compared to their caffeine-based counterparts. However, all derivatives are considered hemocompatible (hemolytic activity < 10%). Notably, derivatives with different predicted pharmacokinetic properties exhibit similar effects on RBCs’ membrane integrity. These data suggest that hemolytic activity is primarily determined by specific structural features, such as the type of alkaloid scaffold or the type of substituents within the uracil ring, rather than by general drug-like descriptors.

### 2.4. Chelating Activity of Ferrous Ions (Fe^2+^)

The ability to chelate ferrous ions (Fe^2+^) is one of the important features of an effective antioxidant, as it prevents iron from catalyzing hydroxyl radical generation via Haber–Weiss or Fenton-type reactions [17]. A previous study conducted by our group demonstrated that caffeine derivatives possess strong Fe^2+^-chelating properties [9]. A comparison of our earlier and current results suggests that an increased number of oxygen atoms and free amine groups in a molecule enhances its metal-chelating capacity. Furthermore, as shown in our earlier study, gramine exhibits excellent chelating activity, reaching 89% efficiency in Fe^2+^ binding [10]. Ethylenediaminetetraacetic acid (EDTA), widely used as a reference metal chelator, was also applied in this study due to its high complex stability constant.

**Figure 2:**
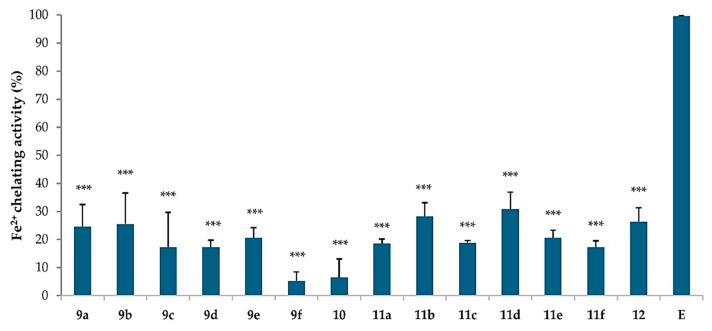
Chelating activity (%) of ferrous ions (Fe^2+^) for novel compounds and the standard chelator ethylenediaminetetraacetic acid (E) at the concentration of 0.01 mg/mL. **9a–10**: hybrid uracil–caffeine derivatives; **11a–12**: hybrid uracil–gramine derivatives. The results are presented as the mean value ± standard deviation (n = 9) in comparison with E activity (*** *p* < 0.001).

As shown in Figure 2, all newly synthesized derivatives exhibit lower chelating activity toward Fe^2+^ compared to the reference chelator EDTA. The highest activity was observed for gramine derivative **11d** (30.86% ± 6.03), while the lowest was recorded for caffeine derivative **9f** (5.28% ± 3.17).

In the case of caffeine derivatives, introducing a triazole ring into the structure results in only a modest increase in chelating activity compared to the parent caffeine molecule, which exhibits relatively low Fe^2+^ chelating capacity (11.25% ± 2.84) [25]. The chelating activity of the new caffeine derivatives ranges from 5.28% to 25.55%. Among them, compound **9b**, containing a phenyl group, shows the highest activity (25.55% ± 10.94). Notably, the most active caffeine derivative **9b** shows approximately 2.3 times greater chelating activity than the parent caffeine molecule. In contrast, the presence of an amino group at the C-4 position of the pyrimidine ring markedly reduces chelating efficiency. Similarly, for the gramine derivatives, the incorporation of a triazole ring and a uracil moiety tends to diminish chelating activity. Nonetheless, gramine-based derivatives generally demonstrate higher chelating activity than caffeine-based derivatives, although this activity is still relatively low overall. As in the caffeine series, the lowest activity among gramine derivatives was observed for compound **11f** (17.29% ± 2.21), which also contains an amino group.

In conclusion, although none of the tested derivatives demonstrated high chelating activity compared to EDTA, certain caffeine derivatives (**9a**, **9b**, and **9e**) demonstrated improved activity relative to the parent caffeine molecule, indicating that structural modifications can enhance Fe^2+^-chelating capacity within this series.

### 2.5. Cytoprotective Activity

ROS are continuously generated within cells and are typically maintained at a physiological level through a balance between pro-oxidant and antioxidant systems. However, various internal and external factors can disrupt this equilibrium, leading to oxidative stress and, in consequence, cellular damage [26]. To mitigate the harmful effects of ROS, new compounds with antioxidant properties are still designed and evaluated using in vitro cell-based assays that assess their ability to neutralize free radicals. Among available methods, RBCs-based assays offer key advantages, such as biological relevance and predictive value, making them a valuable tool in the early stage of drug discovery [27]. To generate in vitro an oxidative stress condition, 2,2′-azobis [2-methylpropionamidine] dihydrochloride (AAPH), a hydrophilic free radical generator, is widely used. Upon thermal decomposition (37 °C), AAPH generates peroxyl and alkoxyl radicals at a controlled and reproducible rate, making it well-suited for studies on oxidative stress and antioxidant activity [28]. Antioxidants act by scavenging or neutralizing highly reactive free radicals. Previous studies from our group have demonstrated that certain derivatives of both caffeine and gramine protect RBCs from free radical-induced hemolysis, supporting their potential as antioxidant agents [9,11,29].

**Figure 3:**
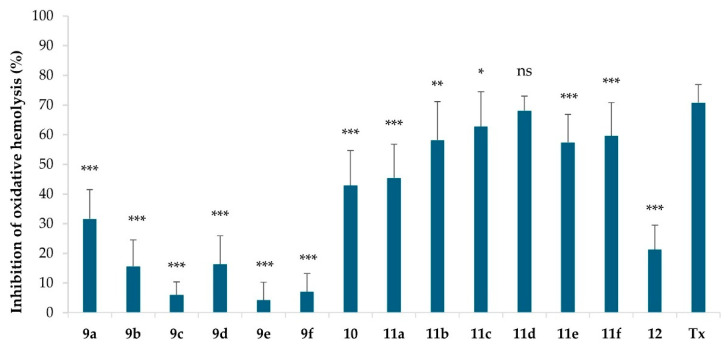
Cytoprotective activity of novel compounds and the standard antioxidant Trolox (Tx) at the concentration of 0.01 mg/mL under oxidative stress conditions (60 mM AAPH, 240 min, 37 °C). **9a–10**: hybrid uracil–caffeine derivatives, **11a–12**: hybrid uracil–gramine derivatives. The results are presented as the mean value ± standard deviation (n = 9) in comparison with Tx activity (* *p* < 0.05, ** *p* < 0.01, *** *p* < 0.001). Non-statistically significant difference is indicated as ns.

As shown in Figure 3, all tested compounds demonstrated the ability to protect human RBCs from oxidative hemolysis induced by free radicals generated by AAPH. Among the evaluated compounds, the most potent cytoprotective activity was observed for gramine-based compounds **11d** (68.07% ± 4.89), **11c** (62.75% ± 11.74), and **11e** (57.40% ± 9.42). Compound **11d**, bearing an n-propyl substituent at the C-5 position, was the most effective, with an activity level statistically comparable to that of Trolox, a reference standard antioxidant (*p* > 0.05). Notably, compound **11d** also exhibited the strongest Fe^2+^ chelating activity (30.86% ± 6.03) as shown in Figure 2, suggesting that its pronounced cytoprotective effect may, at least in part, result from its chelating properties. The lowest cytoprotective activity was observed for compound **12** (21.28% ± 8.24), the only molecule in which both nitrogen atoms in the uracil moiety are substituted. This finding indicates that the presence of a secondary amine group in the pyrimidine ring plays a role in cytoprotective activity. Additionally, electrostatic interactions with cell membrane lipids and/or incorporation of gramine derivatives into the cell membrane may enhance membrane structure stability under oxidative stress conditions [12]. Additionally, gramine and uracil bioconjugates without the triazole ring exhibited lower cytoprotective activity (e.g., 28.20%, 32.16%, 37.51%, and 17.81% in [23]) compared to the gramine–uracil bioconjugates containing a triazole ring, compounds **11b**, **11d**, **11e**, and **12**, which showed activities of 58.14% ± 13.03, 68.07% ± 4.89, 57.40% ± 9.42, and 21.28% ± 8.24, respectively. This suggests that the triazole linker contributes positively to cytoprotective efficacy under oxidative stress.

Among the caffeine-based derivatives, the most active compounds were **10** (42.88% ± 11.77), bearing a fluorine atom at the C-5 position, and **9a** (31.61% ± 9.81), which contains an unsubstituted uracil moiety. In contrast, derivatives substituted at the C-6 position exhibited markedly lower cytoprotective activity, with compound **9e** (4.26% ± 5.97), bearing a n-propyl group at the C-6 position.

Caffeine primarily exhibits antioxidant activity against hydroxyl radicals (^●^OH) through a radical adduct formation (RAF) mechanism [30]. The antioxidant mechanisms of indole compounds, as well as uracil and its derivatives, depend mainly on single-electron transfer (SET) [31] and hydrogen atom transfer (HAT) [32]. It has been demonstrated that the aromatic nature of the indole moiety containing an NH group is crucial for the antiradical activity of indole derivatives [33]. Compound **12**, which contains two indole groups, exhibits very low cytoprotective activity (6.36% ± 4.79). This suggests that the nature of the substituent at the C-3 position of the indole, rather than the presence of the NH group, plays an important role in their cytoprotective activity. The presence of a triazole ring, a purine or indole system, and a pyrimidine moiety in the tested compounds suggests the involvement of both the RAF mechanism, facilitating stable radical adduct formation [9], and the SET mechanism, which stabilizes radicals via electron transfer within the aromatic system [10]. The antioxidant properties of the newly synthesized compounds are also influenced by substituents present in the uracil ring that act as electron-donating (EDG) or electron-withdrawing (EWG), with EWG generally enhancing and EDG reducing antioxidant activity [34].

Among the compounds tested, the highest antioxidant activity was observed for compounds **10** (a caffeine hybrid) and **11d** (a gramine hybrid), both of which contain a fluorine atom and an n-propyl group, respectively, at the C-5 position of the uracil ring.

In the caffeine series, the substitution of fluorine at the C-5 position proved beneficial, since fluorine acts as a weak EDG [34]. In contrast, substitution at the C-6 position of the uracil ring reduced the cytoprotective activity compared to the unsubstituted uracil moiety. This trend may be explained by the fact that substitution at the C-5 position allows for more effective radical stabilization during proton transfer, owing to its proximity to the carbonyl group.

### 2.6. Spectral Scans of Hemoglobin

Hemoglobin (Hb) is the main protein of RBCs responsible for the binding and transport of oxygen to all tissues. Under both physiological and oxidative stress conditions, Hb in its ferrous form (Fe^2+^) is continuously oxidized to methemoglobin (MetHb), the ferric form (Fe^3+^), which is incapable of binding oxygen [16]. Despite this, the concentration of MetHb in the circulation remains stable (typically between 1 and 3%) due to the enzymatic antioxidant system of RBCs and the presence of endogenous and plasma-derived antioxidant molecules.

**Figure 4:**
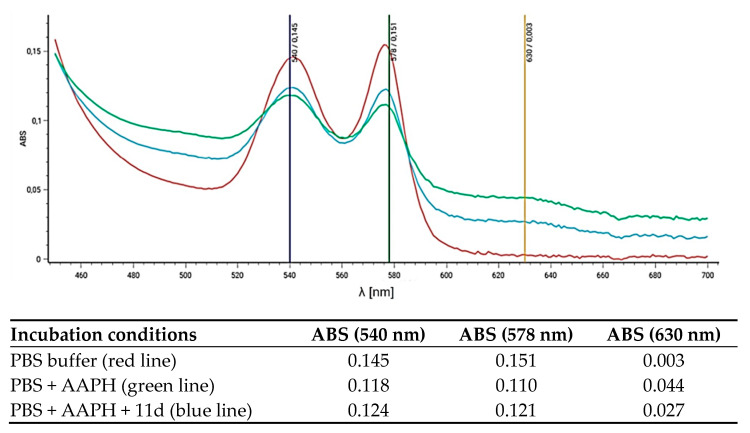
Spectral scans (450–700 nm) of hemoglobin after 4 h of incubation of RBCs in the following: PBS (red line); in PBS with 60 mM of AAPH (green line); PBS with 60 mM of AAPH and derivative **11d** (0.01 mg/mL). Absorbance values (ABS) at 540 nm and 578 nm (oxy-Hb peaks) and 630 nm (MetHb peak) are provided for each scan in the accompanying table. Representative data from a series of experiments are shown.

As demonstrated in our previous study, selected caffeine derivatives protect Hb from oxidation to MetHb [9]. To further evaluate the antioxidant potential of selected derivatives that exhibited the highest cytoprotective activity in RBCs, the spectral scans of Hb in the 450–700 nm range were recorded. As shown in Figure 4, oxyhemoglobin (oxy-Hb) is characterized by two distinct peaks at 540 and 570 nm. Upon the formation of MetHb, these peaks diminish, and a new absorbance peak appears at 630 nm, characteristic of MetHb (Figure 4, green and blue lines). In the presence of derivative **11d**, a decrease in the MetHb absorbance peak at 630 nm was observed (Figure 4, blue line), with the absorbance value dropping from 0.044 to 0.027 (see values in accompanying table in Figure 4). These results indicate that compound **11d** protects Hb inside RBCs from AAPH-induced oxidative conversion to MetHb, further confirming its role as a potent antioxidant under oxidative stress conditions.

### 2.7. Molecular Docking

Molecular docking is a structure-based drug design method that simulates molecular interactions, predicting both the binding mode and affinity between ligands and their target receptors [35]. Compound **11d**, a gramine derivative, was selected for docking studies due to its highest cytoprotective properties (see Figure 3).

As shown in Table 2, compound **11d** exhibits a notable affinity for the analyzed protein domains. The results indicate that its binding affinity is comparable to, or even exceeds, that of the reference ligands: melatonin (1DNU), febuxostat (1N5X), and indomethacin (4COX). Notably, for the 1N5X protein domain, compound **11d** demonstrates a higher binding affinity than febuxostat, the reference ligand. Similarly, in the case of the 4COX protein domain, compound **11d** demonstrates a slightly stronger affinity than the native ligand indomethacin.

These results suggest that the newly synthesized gramine derivative may effectively bind to the investigated protein targets, outperforming established reference ligands.

**Figure 5:**
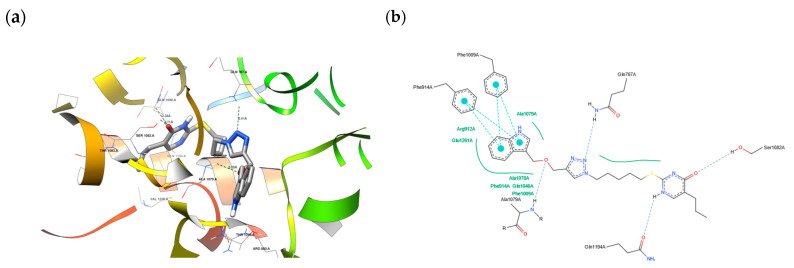
(**a**) The depiction of possible hydrogen bond formation between **11d** and 1N5X protein domain. (**b**) The 2D depiction of interactions between **11d** and the 1N5X protein domain. Blue dashed lines—hydrogen bonds, cyan lines with big dots—pi–pi interactions, and green solid lines—hydrophobic contacts.

Figure 5a,b present the interactions between compound **11d** and the 1N5X protein domain (PDB ID). Importantly, the docking protocol successfully reproduces the native ligand’s initial pose (TEI) with high accuracy, achieving a Root Mean Square Deviation (RMSD) of 1.199 Å and a binding energy of −8.3 kcal/mol [36]. These visualizations offer valuable insights into the molecular interactions that stabilize the binding of compound **11d** to the protein domain, supporting its potential as a promising candidate for further pharmacological investigation.

### 2.8. Fungicidal and Antibacterial Activity

The antifungal activity of derivatives was evaluated in vitro using the well diffusion method against *Fusarium culmorum*, *Fusarium graminearum*, *Trichoderma atroviridae*, *Trichoderma harzianum*, *Alternaria*, and *Botrytis cinerea*.

As shown in Table 3, compounds **9e** (a caffeine derivative) and **11d** (a gramine derivative) exhibited the highest inhibitory activity against *B. cinerea*. Compounds **9c** and **11a** demonstrated moderate antifungal activity against *A. alternata*, as indicated by the growth inhibition zones. Furthermore, compounds **9a**, **9f**, and **12** showed the strongest antagonistic effect toward *F. culmorum*, which is a significant wheat pathogen responsible for seedling blight, foot rot, and *Fusarium* head blight (FHB), and it is predominantly found in cooler regions such as Northern, Central, and Western Europe [37]. In the case of *T. atroviridae*, all tested compounds exhibited low activity, with the exception of compound **11e**. Compound **12** displayed the highest inhibitory activity against *F. graminearum.*

Schreiber et al. reported that gramine can effectively reduce the severity of *Fusarium graminearum* infection in wheat [38] Our previous studies showed that gramine alone has no activity against *T. harzianum* and *F. culmorum* but exhibited moderate activity against *B. cinerea*, *T. prome*, and *A. alternata* [14]. Based on the data in Table 3, it can be concluded that the incorporation of a triazole and uracil ring enhances the antifungal activity of gramine against most of the tested fungal species, except *F. graminearum*. To the best of our knowledge, the antifungal activity of caffeine and its derivatives against the aforementioned fungi has not been previously investigated.

None of the tested compounds exhibited significant bactericidal activity (Table S2, Supplementary Material).

## 3. Materials and Methods

### 3.1. Chemistry

^1^H NMR and ^13^C NMR spectra were recorded on a Varian 400 spectrometer with CDCl_3_ or DMSO-*d*_6_ as the solvent and TMS as the internal standard. The chemical shifts are reported in δ (parts per million) values. ESI mass spectra were measured with the ZQ Waters apparatus and EI mass spectra with the GC Bruker apparatus. FT-IR spectra were recorded on a Nicolet iS 5 (Thermo Scientific, Walthmam, MA, USA) (KBr pellets). TLC analysis was performed using silica gel 60 plates (Sigma-Aldrich, Burlington, MA, USA) with a fluorescent indicator (254 nm) and visualized under UV.

6-Methyl-2-thiouracil was obtained according to [39], 2-thiocytosine according to [40], and 5-propyl-2-thiouracil according to [41]. 5-Fluorouracil, 6-phenyl-2-thiouracil, 6-propyl-2-thiouracil, and thiouracil were commercially available. 8-Mercaptocaffeine, compound **2,** was synthesized according to the procedure from [21]. 3-Propargyloxy-indole-3-carbinol, compound **6**, was synthesized from gramine, compound **4**, according to [12].

Synthesis of compound **3**

1,3,7-trimethyl-8-(prop-2-yn-1-ylthio)-3,7-dihydro-1H-purine-2,6-dione

K_2_CO_3_ (2 mmol) was added to 8-thiocaffeine (1 mmol) dissolved in dimethylformamide (6 mL). The mixture was stirred for 30 min at room temperature. Propargyl bromide (1.5 mmol) was then added dropwise. The reaction mixture was stirred for 1 h at room temperature. The reaction was monitored by TLC (PE: EtOAc 3:7). After the reaction was complete, water (18 mL) was added, and the crude product was precipitated as a brown solid and then purified by column chromatography (CHCl_3_: EtOAc 10:1).

General procedure for preparing bromopentane thiouracil derivatives (compounds **7a–f**)

Newly synthesized bioconjugate structures contain a five-carbon linker. The length of a carbon linker affects the steric effect, the molecule’s mobility, the binding to active sites, for example, in enzymes, and the solubility in water. The carbon chain cannot be too short or too long [42]. Therefore, a 5-carbon linker was chosen.

1,5 Dibromopentane (3 mmol) was added to the appropriate thiouracil derivative (1 mmol) dissolved in a 0.1 M methanolic solution of NaOH (25 mL). The colorless mixture was stirred for four days at room temperature. The reaction progress was monitored by TLC (CHCl_3_: EtOAc, 1:1). After the reaction was completed, the mixture was acidified with 1 M HCl to a pH of approximately 3, then concentrated under reduced pressure, and stored overnight in the refrigerator. After this time, the crude product was precipitated, filtered off, and purified by column chromatography (CHCl_3_: EtOAc 10:1).

Synthesis of bromopentane fluorouracil derivative (compound **8**)

K_2_CO_3_ (2 mmol) was added to 5-fluorouracil (1 mmol) dissolved in dimethylformamide (8 mL). The mixture was stirred for 30 min at room temperature. 1,5 Dibromopentane (1 mmol) was then added. The reaction mixture was stirred for 96 h at room temperature. The reaction progress was monitored by TLC (CHCl_3_: MeOH 15:1). After the reaction was completed, the mixture was acidified with 1M HCl to a pH of approximately 3, and the crude product was precipitated, filtered, and purified by column chromatography (CH_2_Cl_2_: MeOH 25:1).

Standard procedure for synthesizing azidopentane derivatives

Appropriate bromopentane-uracils (1 mmol) were dissolved in DMF (2 mL). Sodium azide (5 mmol) was dissolved in distilled water. Both solutions were mixed. The reaction mixture was stirred for 36 h on a magnetic stirrer and heated for 8 h in a water bath. The reaction progress was monitored by TLC (CHCl_3_: EtOAc 1:1). After this time, distilled water (5 mL) was added. The reaction mixture was extracted with Et_2_O (3 × 40 mL). The organic layer was washed with distilled water (3 × 40 mL) and brine (100 mL). The solution was dried over anhydrous Na_2_SO_4_ and concentrated under reduced pressure. A white precipitate was obtained.

General procedure for preparing compounds **9a–f**, **10**, **11a–f**, **12**

Propargyl derivatives of caffeine or gramine, compounds **3** or **6** (1 mmol), were dissolved in DMSO (10 mL). A suitable azide (1 mmol) was then added. CuSO_4_•5H_2_O (5 mg) and sodium ascorbate (13 mg) were added in 0.5 mL of distilled water. The mixture was stirred at room temperature for one hour. The reaction progress was monitored by TLC (PE: EtOAc 3:7, for caffeine and PhMe: EtOAc 1:1, for gramine). After the reaction was completed, 30 mL of water was added. The resulting mixture was extracted with EtOAc (3 × 40 mL) and washed with water (3 × 40 mL) and brine (100 mL). The solvent was evaporated under reduced pressure to obtain the crude product and purified by column chromatography (CH_2_Cl_2_: MeOH 25:1 for caffeine and CHCl_3_: MeOH 10:1 for gramine).

### 3.2. Biological Activity

#### 3.2.1. Human Erythrocyte

All methods were carried out following relevant guidelines and regulations, and the Bioethics Committee approved all experimental protocols for Scientific Research at the Medical University of Poznań (agreement no. ZP/2867/D/21). Human red blood cell (RBC) concentrates (65% hematocrit) were purchased from the Blood Bank in Poznań without any contact with blood donors.

RBCs were washed three times (3000 rpm, 10 min, +4 °C) in 7.4 pH phosphate-buffered saline (PBS: 137 mM of NaCl, 2.7 mM of KCl, 10 mM of Na_2_HPO_4_, and 1.76 mM of KH_2_PO_4_) supplemented with 10 mM of glucose. After washing, RBCs were suspended in the PBS buffer at 1.65 × 10^9^ cells/mL (15% hematocrit), stored at +4 °C, and used within 5 h.

#### 3.2.2. Hemolytic Activity

Hemolytic activity of newly synthesized compounds was tested according to the protocol described in [43]. RBCs (1.65 × 10^8^ cells/mL, 1.5% hematocrit) were incubated with the tested compounds at a concentration of 0.01 mg/mL for 60 min at 37 °C under shaking. RBCs incubated in PBS buffer and ice-cold water were negative and positive controls, respectively. Each sample was prepared in triplicate, and the experiment was carried out three times. After incubation, the samples were immediately placed on ice to stop the hemolytic process. Subsequently, an appropriate volume of PBS buffer was added to each sample to obtain a sufficient volume of supernatant for absorbance (Ab) measurement. Following centrifugation (4000 rpm, 5 min, +4 °C), the absorbance of the supernatants was measured at 540 nm using a spectrophotometer. The hemolytic activity of each compound was calculated using the formula:

Hemolytic activity (%) = Ab_tested sample_/Ab_positive control_ × 100

where positive control is Ab of the supernatant of RBCs in ice-cold H_2_O (100% hemolysis). The results were presented as the mean value ± standard deviation (n = 9).

#### 3.2.3. Chelating Activity on Ferrous Ions (Fe^2+^)

The chelating activity was evaluated by inhibition of the formation of the Fe^2+^-ferrozine complex after incubation of the compounds tested with Fe^2+^. The experiment was carried out two times. The Fe^2+^-chelating ability was determined by the absorbance of the ferrous ion-ferrozine complex at 562 nm. In brief, 0.1 mg/mL concentration of the compounds tested in 0.2 mL of ethyl alcohol was added to a solution of 0.6 mM of FeCl_2_ (0.02 mL). EDTA was used as the standard chelating agent. The reaction started by adding 5 mM of ferrozine (0.04 mL) in ethyl alcohol, followed by immediate vigorously shaking. The samples were incubated at room temperature for 10 min. Following incubation, the absorbance (Ab) of the solutions was measured at 562 nm in a spectrophotometer. The percentage of inhibition of ferrozine–Fe^2+^ complex formation was calculated using the equation:

Fe^2+^ chelating activity (%) = [1 − (Ab_1_ − Ab_0_)] × 100

where Ab_0_ is the absorbance of the sample without the tested compound, and Ab_1_ is the absorbance in the presence of the compound tested or EDTA. The results were presented as the mean value ± standard deviation (n = 9).

#### 3.2.4. Cytoprotective Activity

RBCs (1.65 × 10^8^ cells/mL, 1.5% hematocrit) were preincubated (20 min at 37 °C with shaking) in PBS buffer (pH 7.4) supplemented with 10 mM of glucose and the tested compounds at a concentration of 0.01 mg/mL. Trolox (a water-soluble analog of vitamin E) was used as a reference antioxidant at the same concentration (0.01 mg/mL). After pre-incubation, 1 M 2,2’-azobis[2-methylpropionamidine] dihydrochloride (AAPH), a hydrophilic free radical generator, was added to achieve a final concentration of 60 mM. Each sample was prepared in triplicate, and the experiment was carried out three times. The samples were incubated for an additional 240 min at 37 °C with shaking. RBCs incubated in PBS alone and in the presence of AAPH served as the negative and the positive controls, respectively. Following incubation, the samples were immediately placed on ice to stop oxidative stress-induced hemolysis. Subsequently, an appropriate volume of PBS buffer was added to each sample to obtain a sufficient volume of supernatants for absorbance (Ab) measurement. After centrifugation (4000 rpm, 5 min, +4 °C), the absorbance of the supernatants was measured at 540 nm using a spectrophotometer. The inhibition of AAPH-induced hemolysis by the tested compounds and Trolox was calculated using the following equation:

Inhibition of oxidative hemolysis (%) = 100 − [(Ab_sample+AAPH_/Ab_AAPH_) × 100]

where Ab_sample_ is the absorbance value obtained from samples incubated with the tested compounds or Trolox, and AAPH, Ab_AAPH_ is the absorbance value obtained from samples incubated with AAPH only. The results were presented as the mean value ± standard deviation (n = 9).

#### 3.2.5. Fungicidal and Antibacterial Activity

The antifungal activity of the compounds was determined against *Alternaria alternata*, *Fusarium culmorum*, *Trichoderma harzianum*, *Trichoderma harzianum*, and *Botrytis cinerea.* The antibacterial properties of the compounds were determined against selected bacteria: *Micrococcus luteus*, *Bacillus subtilis*, *Escherichia coli*, and *Pseudomonas fluorescens.* All the cultures of microorganisms were obtained from the pure culture collection of the Microbiology Department of the Faculty of Soil Science and Microbiology of the Poznan University of Life Sciences.

The well diffusion method was used to evaluate the antimicrobial properties of the compounds. A broth medium was used for bacterial tests, while potato dextrose agar (PDA) was used for mold cultivation. Next, 6 mL of each liquidized medium was poured into sterile Petri dishes and allowed to solidify. After this, two 0.5 cm-diameter sterile glass rings were placed on the surface of each plate. Then, 20 mL of each liquid medium containing suspensions of the tested microorganisms was added. The final bacterial suspension had a density of 107 cells/cm^3^, obtained from 48 h cultures on broth slants, and the fungal suspension had a density of 108 spores/cm^3^, obtained from 5-day cultures on PDA slants. After the medium solidified, the glass rings were removed with a pencil, leaving two wells on each plate. Then, 0.1 mL of the compound dissolved in pure dimethyl sulfoxide was added to one well, and 0.1 mL of pure dimethyl sulfoxide was added to the other well, which served as a control. Each compound was tested in four replicates. Plates were incubated in a thermostat at 27 °C for *M. luteus*, *B. subtilis*, and *P. fluorescens* cultures, as well as at 37 °C for the *E. coli* culture for 48 h. All fungal cultures were incubated at 24 °C for five days in a thermostat. At the end of the incubation, the growth inhibition diameters of the tested strains were measured using calipers.

### 3.3. Physicochemical Data

The physicochemical properties of the newly synthesized molecules, such as lipophilicity, water solubility, and gastrointestinal (GI) absorption, were analyzed using the SwissADME server (www.swissadme.ch) (accessed on 6 May 2024). The molecular polar surface area (PSA) was determined through the topological polar surface area (TPSA) method, which accounts for phosphorus and sulfur atoms as polar. Lipophilicity was expressed as the partition coefficient (log P) between n-octanol and the aqueous phase.

### 3.4. Molecular Docking

The molecular docking process began by converting the SMILES representation of the given chemical structures into 3D structures. This conversion was achieved using the OpenBabel Python library (pybel, version 3.1.0) [44] and biopandas (version 0.5.1) [45]. Next, the protein domains were prepared corresponding to PDB [46] IDs 1DNU [47], 1N5X [48], and 4COX [49] following the standard AutoDock 1.5.7 scheme [50]. Subsequently, molecular dockings were performed using AutoDock Vina [51]. The specific parameters for each docking search were outlined in Table 4 and determined based on the native ligand coordinates: NAG C 620, TEI A, and IMN A for the 1DNU, 1N5X, and 4COX protein domains, respectively.

The selection of protein domains was guided by their biological functions within the physiological system. Among the selected proteins, myeloperoxidase (MPO), xanthine dehydrogenase, and cyclooxygenase-2 (COX-2) play critical roles in cellular processes. Targeting these proteins can significantly impact modulating oxidative stress. They generate reactive oxygen species (ROS) as part of the body’s defense mechanism against pathogens or as a by-product of their enzymatic activity. Inhibiting their function can reduce ROS production and mitigate oxidative stress [52,53,54,55,56,57].

### 3.5. Statistical Analysis

For the antioxidant and cytoprotective properties, data were plotted as the mean value ± standard deviation (SD) of the results of three independent experiments, with every sample taken in triplicate (n = 9). A paired *t*-Student test was used to compare the derivatives’ activity with the activity of the standards, Trolox, or EDTA, respectively. Statistical significance was defined as *p* < 0.05. Non-statistically significant difference is indicated as n.s.

## 4. Conclusions

Fourteen novel compounds were synthesized using click chemistry, characterized, and evaluated for selected biological activities. The results demonstrate that the type of alkaloid moiety plays a crucial role in modulating biological activity—gramine derivatives generally exhibited higher activity compared to caffeine-based analogs. Although the nature of the substituent on the uracil ring appears to be less critical, its position proved to be significant. Notably, compounds **10** (a caffeine hybrid) and **11d** (a gramine hybrid), bearing fluorine and propyl substituents at the C-5 position of the uracil ring, respectively, displayed the highest biological activity within their series. The incorporation of a triazole ring enhanced cytoprotective properties under oxidative stress conditions. In particular, compound **11d** most effectively protected the erythrocyte membrane and hemoglobin from oxidative damage. As erythrocytes serve as a model for studying the cell membrane-targeted bioactive compounds, these findings suggest that compound **11d** may also exert cytoprotective effects in other human cells. Molecular docking studies further supported the antioxidant potential of compound **11d**, revealing strong binding affinity to relevant protein domains compared to reference ligands. Most synthesized compounds demonstrated moderate fungicidal activity. Compounds **9a**, **9f**, and **12** showed the strongest inhibition of *Fusarium culmorum*, a major pathogen of cereal crops. These results highlight the potential of the new hybrids as environmentally friendly fungicidal agents created on natural scaffolds. No antibacterial activity was observed among the tested compounds. In summary, this study provides insight into the structure–activity relationships of alkaloid–uracil hybrids and supports their further development as biologically active compounds with biomedical, agricultural, and ecological relevance.

## Figures and Tables

**Table 1:** Predicted physico-chemical properties of the synthesized compounds (http://www.swissadme.ch) (accessed on 6 May 2024).

Compound	MW [g/mL]	logP	HBD	HBA	TPSA [Å^2^]	GI ABS	BBB Perm	Lipinski’s Rule
**9a**	503.6	1.47	1	7	188.88	low	no	No
**9b**	579.7	2.67	1	7	188.88	low	no	No
**9c**	517.6	1.81	1	7	188.88	low	no	No
**9d**	545.7	2.45	1	7	188.88	low	no	no
**9e**	545.7	2.51	1	7	188.88	low	no	no
**9f**	502.7	0.9	2	7	193.54	low	no	no
**10**	505.5	1.06	1	8	172.69	low	no	no
**11a**	424.52	2.82	2	5	126.78	high	no	yes
**11b**	500.62	4.17	2	5	126.78	low	no	yes
**11c**	438.55	3.07	2	5	126.78	high	no	yes
**11d**	466.6	3.77	2	5	126.78	low	no	yes
**11e**	466.6	3.82	2	5	126.78	low	no	yes
**11f**	425.55	2.51	3	5	131.44	high	no	yes
**12**	722.81	4.92	2	9	155.46	low	no	no

**Table 2:** The results of molecular docking to the 1DNU, 1N5X, and 4COX protein domains of the analyzed compound **11d**. Melatonin, febuxostat, and indomethacin are used as reference molecules. The standard of binding energy is calculated based on the nine best poses.

PDB ID	Compound	Binding Energy [kcal/mol]	Standard Deviation of Binding Energy [kcal/mol]
1DNU	Melatonin	−5.6	0.19
	**11d**	−6.3	0.17
1N5X	Febuxostat	−7.4	0.50
	**11d**	−10.6	0.54
4COX	Indomethacin	−8.6	0.45
	**11d**	−9.4	0.29

**Table 3:** Antifungal activity of all new compounds. Growth inhibition zones: < 9 mm—low active compounds; 10–15 mm—medium active compounds; and >15 mm—active compounds.

Compound	*Fusarium culmorum*	*Fusarium graminearum*	*Trichoderma atroviridae*	*Trichoderma harzianum*	*Alternaria alternata*	*Botrytis cinerea*
**9a**	16	0	8	7	6.5	5.5
**9b**	1	2.5	0	0	7	12
**9c**	0	0	9	6	10	0
**9d**	1	4	0	0	7	13.4
**9e**	1.2	6.4	4.5	0	8	17
**9f**	16	0	8.5	6.5	4	5.5
**10**	10.5	0	4	8	8	0
**11a**	1.5	10	4.5	8.4	11.2	11.8
**11b**	0	0	8	10	7	0
**11c**	0	0	7.5	7	0	6.5
**11d**	1	3	9	0	9	16
**11e**	0	0	12	8.5	8.5	6
**11f**	3	0	7	6.5	8.5	6
**12**	15	12	6	8	6.5	5

**Table 4:** The search spaces of the analyzed binding sites of the protein domains.

PDB ID	Search Space Center (x, y, z)	Size of the Search Space (x, y, z)
1DNU	40, −38, −5	22, 19, 20
1N5X	97, 55, 39	35, 37, 43
4COX	25, 22, 15	38, 37, 40

## Data Availability

The original contributions presented in this study are included in the article. Further inquiries can be directed to the corresponding author.

## Supplementary Materials

The following supporting information can be downloaded at https://www.mdpi.com/article/10.3390/molecules30132714/s1. Figures S1a–S15d: Spectroscopic description of new compounds; Table S1: Solubility in water of all new compounds; Table S2: Antibacterial activity of all new compounds.
