# Supplementary material for: Hybrid Uracil Derivatives with Caffeine and Gramine Obtained via Click Chemistry as Potential Antioxidants and Inhibitors of Plant Pathogens
Source: Molecules. 2025 Jun 24;30(13):2714. doi: 10.3390/molecules30132714 (PMC12251463; doi:10.3390/molecules30132714)
Supplement: Supplementary file 1 [file molecules-30-02714-s001.zip › molecules-3689348-supplementary.pdf]

Supplementary Material

Hybrid Uracil Derivatives with Caffeine and Gramine Obtained via Click Chemistry as Potential Antioxidants and Inhibitors of Plant Pathogens

Milda Szlaużys [1,†], Kamil Ostrowski [1,†], Damian Nowak [2], Wiesław Prukała [3], Justyna Starzyk [4], Beata Jasiewicz [1] and Lucyna Mrówczyńska [5,*]

[1] Department of Bioactive Products, Faculty of Chemistry, Adam Mickiewicz University, Uniwersytetu Poznańskiego 8, 61-614 Poznań, Poland; milda.szlauzys@amu.edu.pl (M.S.); kamil.ostrowski@amu.edu.pl (K.O.); beata.jasiewicz@amu.edu.pl (B.J.)
[2] Department of Quantum Chemistry, Faculty of Chemistry, Adam Mickiewicz University, Uniwersytetu Poznańskiego 8, 61-614 Poznań, Poland; damian.nowak@amu.edu.pl
[3] Department of Natural Products, Faculty of Chemistry, Adam Mickiewicz University, Uniwersytetu Poznańskiego 8, 61-614 Poznań, Poland; wprukala@amu.edu.pl
[4] Department of Soil Science and Microbiology, Faculty of Agronomy, Horticulture, and Bioengineering, University of Life Science, Szydłowska 50, 60-656 Poznań, Poland; jstarzyk@up.poznan.pl
[5] Department of Cell Biology, Faculty of Biology, Adam Mickiewicz University, Uniwersytetu Poznańskiego 6, 61-614 Poznań, Poland
* Correspondence: lucyna.mrowczynska@amu.edu.pl
† These authors contributed equally to this work.

TABLE OF CONTENTS

Spectroscopic description of new compounds *S3*
Figure S1a ^{1}H and **Figure S1b** ^{13}C NMR spectra of compound **3** *S8*
Figure S1c EI-MS and **Figure S1d** FT-IR spectra of compound **3** *S9*
Figure S2a ^{1}H and **Figure S2b** ^{13}C NMR spectra of compound **9a** *S10*
Figure S2c EI-MS and **Figure S2d** FT-IR spectra of compound **9a** *S11*
Figure S3a ^{1}H and **Figure S3b** ^{13}C NMR spectra of compound **9b** *S12*
Figure S3c EI-MS and **Figure S3d** FT-IR spectra of compound **9b** *S13*
Figure S4a ^{1}H and **Figure S4b** ^{13}C NMR spectra of compound **9c** *S14*
Figure S4c EI-MS and **Figure S4d** FT-IR of spectra of compound **9c** *S15*
Figure S5a ^{1}H and **Figure S5b** ^{13}C NMR of spectra of compound **9d** *S16*
Figure S5c EI-MS and **Figure S5d** FT-IR of spectra of compound **9d** *S17*
Figure S6a ^{1}H and **Figure S6b** ^{13}C NMR of spectra of compound **9e** *S18*
Figure S6c EI-MS and **Figure S6d** FT-IR of spectra of compound **9e** *S19*
Figure S7a ^{1}H and **Figure S7b** ^{13}C NMR of spectra of compound **9f** *S20*
Figure S7c EI-MS and **Figure S7d** FT-IR of spectra of compound **9f** *S21*
Figure S8a ^{1}H and **Figure S8b** ^{13}C NMR of spectra of compound **10** *S22*
Figure S8c EI-MS and **Figure S8d** FT-IR of spectra of compound **10** *S23*
Figure S9a ^{1}H and **Figure S9b** ^{13}C NMR of spectra of compound **11a** *S24*
Figure S9c EI-MS and **Figure S9d** FT-IR of spectra of compound **11a** *S25*
Figure S10a ^{1}H and **Figure S10b** ^{13}C NMR of spectra of compound **11b** *S26*
Figure S10c EI-MS and **Figure S10d** FT-IR of spectra of compound **11b** *S27*

Figure S11a ^{1}H and **Figure S11b** ^{13}C NMR of spectra of compound **11c** *S28*
Figure S11c EI-MS and **Figure S11d** FT-IR of spectra of compound **11c** *S29*
Figure S12a ^{1}H and **Figure S12b** ^{13}C NMR of spectra of compound **11d** *S30*
Figure S12c EI-MS and **Figure S12d** FT-IR of spectra of compound **11d** *S31*
Figure S13a ^{1}H and **Figure S13b** ^{13}C NMR of spectra of compound **11e** *S32*
Figure S13c EI-MS and **Figure S13d** FT-IR of spectra of compound **11e** *S33*
Figure S14a ^{1}H and **Figure S14b** ^{13}C NMR of spectra of compound **11f** *S34*
Figure S14c EI-MS and **Figure S14d** FT-IR of spectra of compound **11f** *S35*
Figure S15a ^{1}H and **Figure S15b** ^{13}C NMR of spectra of compound **12** *S36*
Figure S15c EI-MS and **Figure S15d** FT-IR of spectra of compound **12** *S37*
Table S1 Solubility in water of all new compounds *S38*
Table S2 Antibacterial activity of all new compounds *S39*

Compound 3

1,3,7-trimethyl-8-(prop-2-yn-1-ylthio)-3,7-dihydro-1H-purine-2,6-dione

Brownish solid (222 mg, 87%); Mp. 176-177°C; ^{1}H NMR (400 MHz, $CDCl_3$) δ 4.00 (d, *J* = 2.6 Hz, 2H), 3.91 (s, 3H), 3.57 (s, 3H), 3.39 (s, 3H), 2.28 (t, *J* = 2.6 Hz, 1H); ^{13}C NMR (100 MHz, $CDCl_3$) δ 154.6, 151.4, 148.4, 148.3, 109.0, 78.3, 72.4, 32.5, 29.7, 27.9, 21.7; FT-IR: (KBr, cm^{-1}) ν_{max}: 3446.58, 3277.04, 2932.66, 1701.28, 1670.26, 1538.51, 1364.42, 756.85; EI-MS (*m/z*, int%) 263, 10%.

Compound 9a

1,3,7-trimethyl-8-(((1-(5-((6-oxo-1,6-dihydropyrimidin-2-yl)thio)pentyl)-1H-1,2,3-triazol-4-yl)methyl)thio)-3,7-dihydro-1H-purine-2,6-dione

White solid (272 mg, 54%); Mp. 265-266°C; ^{1}H NMR (400 MHz, DMSO-d_6) δ 12.66 (s, 1H), 7.99 (s, 1H), 5.76 (s, 1H), 4.52 (s, 2H), 4.32 (t, *J* = 7.0 Hz, 2H), 3.70 (s, 3H), 3.45 (s, 3H), 3.34 (s, 3H), 3.04 (t, *J* = 7.4 Hz, 2H), 1.79 (q, *J* = 7.1 Hz, 2H), 1.62 (q, *J* = 7.6 Hz, 2H), 1.27 (q, *J* = 7.5 Hz, 2H); ^{13}C NMR (100 MHz, DMSO-d_6) δ 153.7, 150.7, 148.9, 147.7, 142.4, 123.6, 108.0, 54.9, 49.1, 38.9, 32.0, 29.5, 29.3, 29.2, 28.1, 27.5, 27.2, 24.9; FT-IR: (KBr, cm^{-1}) ν_{max}: 3428.77, 2923.31, 2852.56, 1704.07, 1655.29, 1537.96, 1456.21, 1365.96, 1271.40, 1217.41, 1170.79, 980.75, 745.93; ESI-MS (MeOH) (*m/z*, % int.) 504, 100% (M + H^+).

Compound 9b

1,3,7-trimethyl-8-(((1-(5-((6-oxo-4-phenyl-1,6-dihydropyrimidin-2-yl)thio)pentyl)-1H-1,2,3-triazol-4-yl)methyl)thio)-3,7-dihydro-1H-purine-2,6-dione

White solid (174 mg, 30%); Mp. 263-264°C; ^{1}H NMR (400 MHz, DMSO-d_6) δ 12.71 (s, 1H), 7.99 (d, *J* = 6.2 Hz, 3H), 7.45 (dt, *J* = 14.2, 6.9 Hz, 3H), 6.65 (s, 1H), 4.51 (s, 2H), 4.32 (t, *J* = 6.9 Hz, 2H), 3.63 (s, 3H), 3.42 (s, 3H), 3.17 (s, 3H), 1.82 (t, *J* = 7.6 Hz, 2H), 1.75 – 1.67 (m, 2H), 1.34 (q, *J* = 7.4 Hz, 2H); ^{13}C NMR (100 MHz, DMSO-d_6) δ 155.6, 153.7, 150.7, 148.8, 147.7, 142.5, 135.9, 130.5, 128.7, 126.6, 123.7, 108.0, 49.2, 32.0, 29.5, 29.3, 28.3, 27.4, 27.1, 25.1; FT-IR: (KBr, cm^{-1}) ν_{max}: 3445.46, 3133.81, 3056.74, 2934.66, 2855.39, 1701.32, 1660.49, 1538.31, 1462.86, 1365.00, 1215.46, 990.72, 747.91, ESI-MS (MeOH) (*m/z*, % int.) 602, 10% (M + Na^+).

Compound 9c

1,3,7-trimethyl-8-(((1-(5-((4-methyl-6-oxo-1,6-dihydropyrimidin-2-yl)thio)pentyl)-1H-1,2,3-triazol-4-yl)methyl)thio)-3,7-dihydro-1H-purine-2,6-dione

White solid (414 mg, 78%); Mp. 268-269°C; ^{1}H NMR (400 MHz, DMSO-d_6) δ 12.48 (s, 1H), 7.99 (s, 1H), 5.76 (s, 1H), 4.52 (s, 2H), 4.32 (t, *J* = 7.0 Hz, 2H), 3.69 (s, 3H), 3.44 (s, 3H), 3.19 (s, 3H), 3.03 (t, *J* = 7.2 Hz, 2H), 2.13 (s, 3H), 1.79 (q, *J* = 7.1 Hz, 2H), 1.62 (q, *J* = 7.4 Hz, 2H), 1.26 (q, *J* = 7.5, 7.0 Hz, 2H); ^{13}C NMR (100 MHz, DMSO-d_6) δ 153.7, 150.7, 148.9, 147.8, 142.4, 123.6, 123.6, 108.0, 54.9, 49.1, 32.0, 29.5, 29.1, 28.0, 27.5, 27.3, 27.2, 27.2, 24.8, 23.4; FT-IR: (KBr, cm^{-1}) ν_{max}: 3435.12, 3145.48, 2938.29, 2860.79, 2738.65, 1698.32, 1660.59, 1537.85, 1464.37, 1365.29, 1218.27, 1178.17, 953.36, 752.68; ESI-MS (MeOH) (*m/z*, % int.) 518, 100% (M + H+), 540, 80% (M + Na^+).

Compound 9d

1,3,7-trimethyl-8-(((1-(5-((6-oxo-5-propyl-1,6-dihydropyrimidin-2-yl)thio)pentyl)-1H-1,2,3-triazol-4-yl)methyl)thio)-3,7-dihydro-1H-purine-2,6-dione

White solid (98 mg, 18%); Mp. 264-265°C; ^{1}H NMR (400 MHz, DMSO-d$_6$) δ 12.62 (s, 1H), 7.99 (s, 1H), 5.76 (s, 1H), 4.52 (s, 2H), 4.31 (t, *J* = 7.0 Hz, 2H), 3.69 (s, 3H), 3.44 (s, 3H), 3.20 (s, 3H), 3.02 (t, *J* = 7.2 Hz, 2H), 2.23 (s, 2H), 1.81–1.72 (m, 2H), 1.64–1.57 (m, 2H), 1.47 (q, *J* = 7.7 Hz, 2H), 1.25 (d, *J* = 11.6 Hz, 2H), 0.85 (d, *J* = 7.3 Hz, 4H); ^{13}C NMR (100 MHz, DMSO-d$_6$) δ 153.7, 150.7, 150.0, 148.8, 147.8, 142.5, 123.7, 108.0, 54.9, 49.1, 32.0, 29.5, 29.1, 28.1, 27.5, 27.2, 24.9, 21.1, 13.7; FT-IR: (KBr, cm^{-1}) ν_{max}: 3445.71, 3147.65, 2941.36, 2865.06, 1700.11, 1664.16, 1537.20, 1462.94, 1365.68, 1214.00, 1170.03, 992.35, 754.87; ESI-MS (MeOH) (*m*/*z*, % int.) 568, 40% (M + Na$^+$).

Compound 9e

1,3,7-trimethyl-8-(((1-(5-((6-oxo-6-propyl-1,6-dihydropyrimidin-2-yl)thio)pentyl)-1H-1,2,3-triazol-4-yl)methyl)thio)-3,7-dihydro-1H-purine-2,6-dione

White solid (137 mg, 25%); Mp. 259-260°C; ^{1}H NMR (400 MHz, DMSO-d$_6$) δ 7.98 (s, 1H), 5.76 (s, 1H), 4.52 (s, 2H), 4.31 (t, *J* = 7.0 Hz, 2H), 3.70 (s, 3H), 3.45 (s, 3H), 3.20 (s, 3H), 3.04 (t, *J* = 7.2 Hz, 2H), 1.78 (q, *J* = 7.1 Hz, 2H), 1.68–1.52 (m, 5H), 1.33–1.21 (m, 3H), 0.85 (t, *J* = 7.4 Hz, 4H); ^{13}C NMR (100 MHz, DMSO-d$_6$) δ 153.8, 150.7, 148.8, 147.8, 142.4, 123.6, 108.1, 54.9, 49.1, 38.5, 32.1, 29.5, 29.2, 28.2, 27.5, 27.2, 24.9, 20.5, 13.4; FT-IR: (KBr, cm^{-1}) ν_{max}: 3444.54, 2929.63, 2868.77, 1703.31, 1662.81, 1538.51, 1366.36, 1220.35, 971.70, 746.75; ESI-MS (MeOH) (*m*/*z*, % int.) 568, 100% (M + Na$^+$).

Compound 9f

8-(((1-(5-((6-amino-1,6-dihydropyrimidin-2-yl)thio)pentyl)-1H-1,2,3-triazol-4-yl)methyl)thio)-1,3,7-trimethyl-3,7-dihydro-1H-purine-2,6-dione

White solid (180 mg, 36%); Mp. 266-267°C; ^{1}H NMR (400 MHz, DMSO-d$_6$) δ 7.99 (s, 1H), 7.87 (d, *J* = 5.8 Hz, 1H), 6.87 (s, 1H), 6.11 (d, *J* = 5.8 Hz, 1H), 5.76 (s, 1H), 4.52 (d, *J* = 3.8 Hz, 2H), 4.31 (t, *J* = 7.1 Hz, 2H), 3.69 (s, 3H), 3.44 (s, 3H), 3.19 (s, 3H), 2.93 (t, *J* = 7.3 Hz, 2H), 1.77 (q, *J* = 10.6, 9.0 Hz, 3H), 1.59 (q, J = 7.5 Hz, 2H), 1.36–1.21 (m, 2H); ^{13}C NMR (100 MHz, DMSO-d$_6$) δ 169.7, 163.0, 154.8, 153.7, 153.7, 150.7, 150.7, 148.9, 148.9, 147.7, 147.7, 142.4, 142.3, 123.6, 123.6, 108.0, 108.0, 101.1, 54.9, 49.2, 32.0, 29.5, 29.3, 28.5, 27.5, 27.2, 25.1, 22.6; FT-IR: (KBr, cm^{-1}) ν_{max}: 3419.36, 3337.56, 3195.83, 2931.89, 2857.46,1701.66, 1652.91, 1579.64, 1540.22, 1463.43, 1366.64, 1246.83, 1215.62, 974.49, 747.69; ESI-MS (MeOH) (*m*/*z*, % int.) 525, 60% (M + Na$^+$).

Compound 10

8-(((1-(5-(5-fluoro-2,4-dioxo-3,4-dihydropyrimidin-1(2H)-yl)pentyl)-1H-1,2,3-triazol-4-yl)methyl)thio)-1,3,7-trimethyl-3,7-dihydro-1H-purine-2,6-dione

White solid (251 mg, 48%); Mp. 248-249°C; ^{1}H NMR (400 MHz, DMSO-d$_6$) δ 11.95 (s, 1H), 8.14-8.11 (t, 1H), 7.95 (s, 1H), 4.50 (s, 2H), 4.30-4.25 (t, 2H), 3.78-3.74 (t, 2H), 3.68 (s, 3H), 3.43 (t, 3H), 3.32 (s, 3H), 1.77-1.73 (m, 2H), 1.53-1.48 (m, 2H), 1.30-1.25 (m, 2H); ^{13}C NMR (100 MHz, DMSO-d$_6$) δ 156.7, 156.5, 153.7, 150.6, 149.3, 148.9, 147.7, 142.4, 142.4, 140.1, 137.8, 129.3, 128.8, 123.5, 108.0,

49.1, 32.0, 29.4, 29.3, 29.2, 27.4, 27.1, 26.2, 23.0, 22.5; FT-IR: (KBr, cm^{-1}) ν_{max}: 3463.68, 2948.1, 1713.68, 1660.94, 1550.12, 1371.15, 1034.54, 756.03; ESI-MS (MeOH) (*m/z*, % int.) 523, 100% (M + Na^+).

Compound 11a

2-((5-(4-(((1H-indol-3-yl)methoxy)methyl)-1H-1,2,3-triazol-1-yl)pentyl)thio)pyrimidin-4(3H)-one

Brownish oil (84 mg, 20%). ^{1}H NMR 400 MHz, $CDCl_3$) δ 8.69 (s, 1H), 7.81 (d, *J* = 6.6 Hz, 1H), 7.68 (d, *J* = 7.4 Hz, 1H), 7.44 (s, 1H), 7.35 (d, *J* = 8.1 Hz, 1H), 7.20–7.15 (m, 2H), 7.11 (ddd, *J* = 8.1, 7.1, 1.1 Hz, 1H), 6.18 (d, *J* = 6.6 Hz, 1H), 4.80 (s, 2H), 4.69 (s, 2H), 4.29 (t, *J* = 7.1 Hz, 2H), 3.12 (t, *J* = 7.2 Hz, 2H), 1.93–1.82 (m, 2H), 1.76–1.68 (m, 2H), 1.43–1.34 (m, 2H), 1.26 (s, 2H); ^{13}C NMR (100 MHz, $CDCl_3$) δ 164.5, 162.0, 154.8, 145.6, 136.4, 127.1, 124.4, 122.4, 122.2, 119.7, 119.1, 111.3, 110.9, 64.4, 62.9, 49.9, 40.8, 30.1, 29.6, 28.3, 25.3; FT-IR: (KBr, cm^{-1}) ν_{max}: 3443.90, 2923.56, 2854.92, 1651.64, 1538.36, 1538.36, 1468.92, 982.02, 819.70; ESI-MS (MeOH) (*m/z*, % int.) 447, 100% (M + Na^+).

Compound 11b

2-((5-(4-(((1H-indol-3-yl)methoxy)methyl)-1H-1,2,3-triazol-1-yl)pentyl)thio)-6-phenylpyrimidin-4(3H)-one

Brownish oil (95 mg, 19%); ^{1}H NMR (400 MHz, DMSO-d_6) δ 11.04 (s, 1H), 8.07 (s, 1H), 8.05–8.02 (m, 2H), 7.54 (d, *J* = 7.9 Hz, 1H), 7.46 (d, *J* = 5.8 Hz, 3H), 7.37 (d, *J* = 4.8 Hz, 2H), 7.08 (ddd, *J* = 8.2, 7.1, 1.2 Hz, 1H), 7.01–6.96 (m, 1H), 6.66 (s, 1H), 4.67 (s, 2H), 4.51 (s, 2H), 4.35 (t, *J* = 7.0 Hz, 2H), 3.22 (t, *J* = 7.2 Hz, 2H), 1.87 (q, *J* = 7.2 Hz, 2H), 1.75 (q, *J* = 7.5 Hz, 2H), 1.38 (q, *J* = 7.4 Hz, 2H); ^{13}C NMR (100 MHz, DMSO-d_6) δ 144.2, 136.3, 136.0, 130.6, 128.7, 127.0, 126.7, 125.2, 123.7, 122.6, 121.2, 118.7, 118.7, 111.5, 111.3, 63.4, 62.0, 55.1, 49.1, 49.1, 29.6, 29.4, 29.3, 28.3, 25.2; FT-IR: (KBr, cm^{-1}) ν_{max}: 3271.80, 3052.31, 2932.42, 2854.12, 1675.13, 1564.98, 1470.70, 1383.69, 1215.14, 1058.39, 1026.24, 991.07, 928.78, 831.32, 776.20, 686.94, 559.44; ESI-MS (MeOH) (*m/z*, % int.) 523, 100% (M + Na^+).

Compound 11c

2-((5-(4-(((1H-indol-3-yl)methoxy)methyl)-1H-1,2,3-triazol-1-yl)pentyl)thio)-6-methylpyrimidin-4(3H)-one

Brownish oil (130 mg, 27%); ^{1}H NMR (400 MHz, $CDCl_3$) δ 8.40 (s, 1H), 7.69 (d, *J* = 7.9 Hz, 1H), 7.51 (s, 1H), 7.46 (s, 1H), 7.36 (s, 1H), 7.23 (d, *J* = 2.6 Hz, 1H), 7.13 (t, *J* = 1.0 Hz, 1H), 6.02 (s, 1H), 4.82 (s, 2H), 4.70 (s, 2H), 4.32 (d, *J* = 7.1 Hz, 2H), 3.15 (s, 3H), 1.92 (d, *J* = 7.3 Hz, 2H), 1.76–1.72 (m, 7H); ^{13}C NMR (100 MHz, $CDCl_3$) δ 165.8, 164.7, 160.2, 145.7, 136.4, 129.7, 127.1, 124.2, 122.3, 119.8, 119.2, 111.2, 108.2, 64.4, 62.9, 56.5, 49.9, 40.9, 30.1, 29.6, 28.3, 24.2; FT-IR: (KBr, cm^{-1}) ν_{max}: 3396.35, 3250.95, 3055. 39, 2926.85, 2858.28, 1722.54, 1659.47, 1576.37, 1537. 37, 1456.85, 1279. 12, 1229.05, 1177.03, 1022.28, 953.15, 839.33, 746.48; ESI-MS (MeOH) (*m/z*, % int.) 461, 100% (M + Na^+).

Compound 11d

2-((5-(4-(((1H-indol-3-yl)methoxy)methyl)-1H-1,2,3-triazol-1-yl)pentyl)thio)-5-propylpyrimidin-4(3H)-one

Orange oil (205 mg, 44%); ^{1}H NMR (400 MHz, DMSO-d$_6$) δ 9.6 (s, 1H) 7.72 – 7.60 (m, 2H), 7.55 – 7.43 (m, 2H), 7.36 (dd, *J* = 8.0, 3.1 Hz, 1H), 7.24 – 7.08 (m, 3H), 4.80 (s, 2H), 4.67 (s, 2H), 4.29 (q, *J* = 6.9 Hz, 2H), 3.14 – 3.03 (m, 2H), 2.41 – 2.32 (m, 2H), 1.88 (t, *J* = 7.8 Hz, 2H), 1.72 (h, *J* = 7.5 Hz, 4H), 1.61 – 1.52 (m, 2H), 0.93 (t, *J* = 7.3 Hz, 3H). ^{13}C NMR (100 MHz, DMSO-d$_6$) δ 163.7, 158.9, 136.4, 129.6, 127.0, 124.5, 122.4, 121.9, 120.1, 119.4, 118.9, 111.3, 64.4, 62.7, 56.0, 49.8, 29.9, 28.2, 25.2, 21.4, 20.7, 13.7; FT-IR: (KBr, cm^{-1}) ν_{max}: 3421.84, 2925.46, 1668.15, 1447.62, 1039.24, 960.07; ESI-MS (MeOH) (*m*/*z*, % int.) 489, 50% (M + Na$^+$).

Compound 11e

2-((5-(4-(((1H-indol-3-yl)methoxy)methyl)-1H-1,2,3-triazol-1-yl)pentyl)thio)-6-propylpyrimidin-4(3H)-one

Dark red oil (121 mg, 26%); ^{1}H NMR (400 MHz, CDCl$_3$) δ 8.60 (s, 1H), 7.49 (s, 1H), 7.44 (s, 1H), 7.27 (s, 1H), 7.20 (s, 2H), 7.12 (t, *J* = 8.0 Hz, 2H), 6.02 (s, 1H), 4.81 (s, 2H), 4.68 (s, 2H), 4.29 (t, *J* = 7.1 Hz, 2H), 3.13 (d, *J* = 6.8 Hz, 2H), 2.44 (s, 2H), 1.90 – 1.84 (m, 2H), 1.65 (dt, *J* = 7.5, 3.7 Hz, 4H), 1.43 – 1.39 (m, 2H), 0.94 – 0.91 (m, 3H). ^{13}C NMR (100 MHz, CDCl$_3$) δ 169.2, 165.2, 160.2, 145.7, 136.4, 127.1, 124.3, 122.2, 119.7, 119.1, 111.3, 107.7, 77.2, 64.4, 62.8, 50.0, 40.8, 39.5, 30.1, 29.7, 28.2, 25.4, 25.2, 20.9, 13.6; FT-IR: (KBr, cm^{-1}) ν_{max}: 3400.41, 2930.55, 2863.41, 1655.75, 1572.22, 1534.48, 1456.71, 1394.26, 1223.72, 1170.071048.68, 989.37, 744.82; ESI-MS (MeOH) (*m*/*z*, % int.) 489, 70% (M + Na$^+$).

Compound 11f

2-((5-(4-(((1H-indol-3-yl)methoxy)methyl)-1H-1,2,3-triazol-1-yl)pentyl)thio)-3,4-dihydropyrimidin-4-amine

Orange oil (173 mg, 41%); ^{1}H NMR (400 MHz, DMSO-d$_6$) δ 11.04 (s, 1H), 8.09 (s, 1H), 7.87 (d, *J* = 5.8 Hz, 1H), 7.54 (d, *J* = 8.0 Hz, 1H), 7.40–7.33 (m, 2H), 7.12–7.06 (m, 1H), 7.01 (d, J = 7.1 Hz, 1H), 6.89 (s, 2H), 6.11 (d, *J* = 5.8 Hz, 1H), 4.66 (s, 2H), 4.51 (s, 2H), 4.34 (t, J = 7.3 Hz, 2H), 2.97 (t, *J* = 7.3 Hz, 2H), 1.83 (q, *J* = 7.2 Hz, 2H), 1.63 (t, *J* = 7.2 Hz, 2H), 1.32 (q, *J* = 7.6 Hz, 2H); ^{13}C NMR (100 MHz, DMSO-d$_6$) δ 169.7, 163.0, 154.8, 144.2, 136.3, 127.0, 125.2, 123.7, 121.2, 118.7, 118.7, 111.5, 101.1, 63.4, 62.0, 49.1, 40.4, 29.3, 28.5, 25.1; FT-IR: (KBr, cm^{-1}) ν_{max}: 3426.01, 2924.25, 2853.79, 1738.86, 1633.69, 1586.09, 1455.86, 1340.05, 1251.06, 1023.79, 954.44, 725.09; ESI-MS (MeOH) (*m*/*z*, % int.) 443, 100% (M + Na$^+$).

Compound 12

1,3-bis(5-(4-(((1H-indol-3-yl)methoxy)methyl)-1H-1,2,3-triazol-1-yl)pentyl)-5-fluoropyrimidine-2,4(1H,3H)-dione

Yellowish oil (245 mg, 34%); ^{1}H NMR (400 MHz, DMSO-d$_6$) δ 8.48 (s, 1H), 8.44 (s, 1H), 7.69 (d, *J* = 7.8 Hz, 2H), 7.45 (s, 1H), 7.41 (s, 1H), 7.37 (d, *J* = 1.1 Hz, 1H), 7.34 (q, *J* = 1.0 Hz, 1H), 7.32 (t, *J* = 1.0 Hz, 1H), 7.20–7.19 (m, 1H), 7.16 (t, *J* = 1.7 Hz, 1H), 7.15–7.14 (m, 1H), 7.13–7.12 (m, 1H), 7.11 (d, *J* = 0.6 Hz, 1H), 7.02 (s, 1H), 7.01 (s, 1H), 4.83 (d, *J* = 0.7 Hz, 2H), 4.80 (s, 2H), 4.70 (s, 2H), 4.68 (d, *J* = 0.6 Hz, 2H), 4.29 (d, *J* = 7.1 Hz, 3H), 3.92 (t, *J* = 7.3 Hz, 2H), 3.54 (t, *J* = 7.4 Hz, 2H), 1.87 (t, *J* = 7.6

Hz, 5H), 1.64–1.57 (m, 4H), 1.26 (d, *J* = 4.0 Hz, 4H); ^{13}C NMR (100 MHz, DMSO-d_6) δ 157.3, 157.1, 149.7, 145.9, 145.6, 138.7, 136.4, 129.7, 127.1, 126.8, 126.4, 124.3, 122.4, 122.3, 122.2, 119.8, 119.2, 119.1, 112.7, 112.6, 111.3, 111.3, 64.6, 63.0, 49.8, 49.7, 49.5, 41.3, 41.0, 30.0, 29.5, 28.1, 28.1, 26.5, 23.3, 23.1, 22.8; FT-IR: (KBr, cm^{-1}) ν_{max}: 3319.35, 2937.41, 2867.01, 1652.2, 1467.8, 1259.6, 1278.09, 744.66; ESI-MS (MeOH) (*m*/*z*, % int.) 745, 60% (M + Na$^+$).

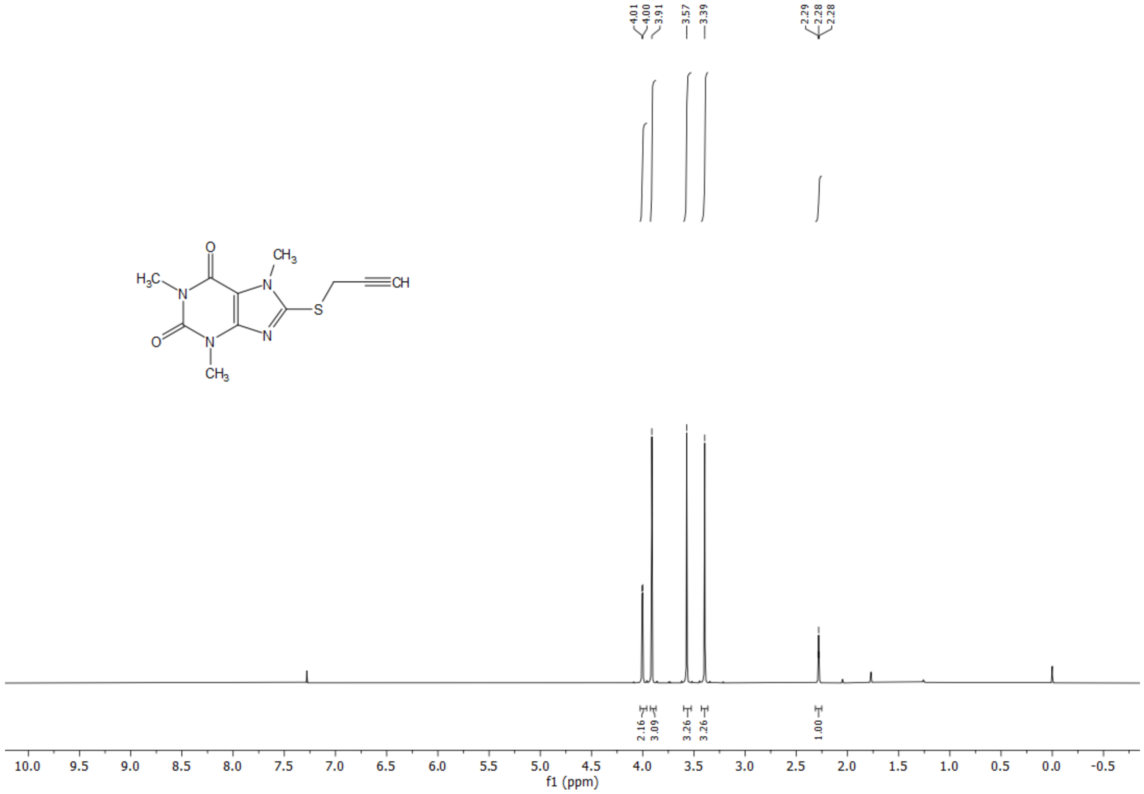

Fig. S1a ^{1}HNMR spectrum of compound **3**

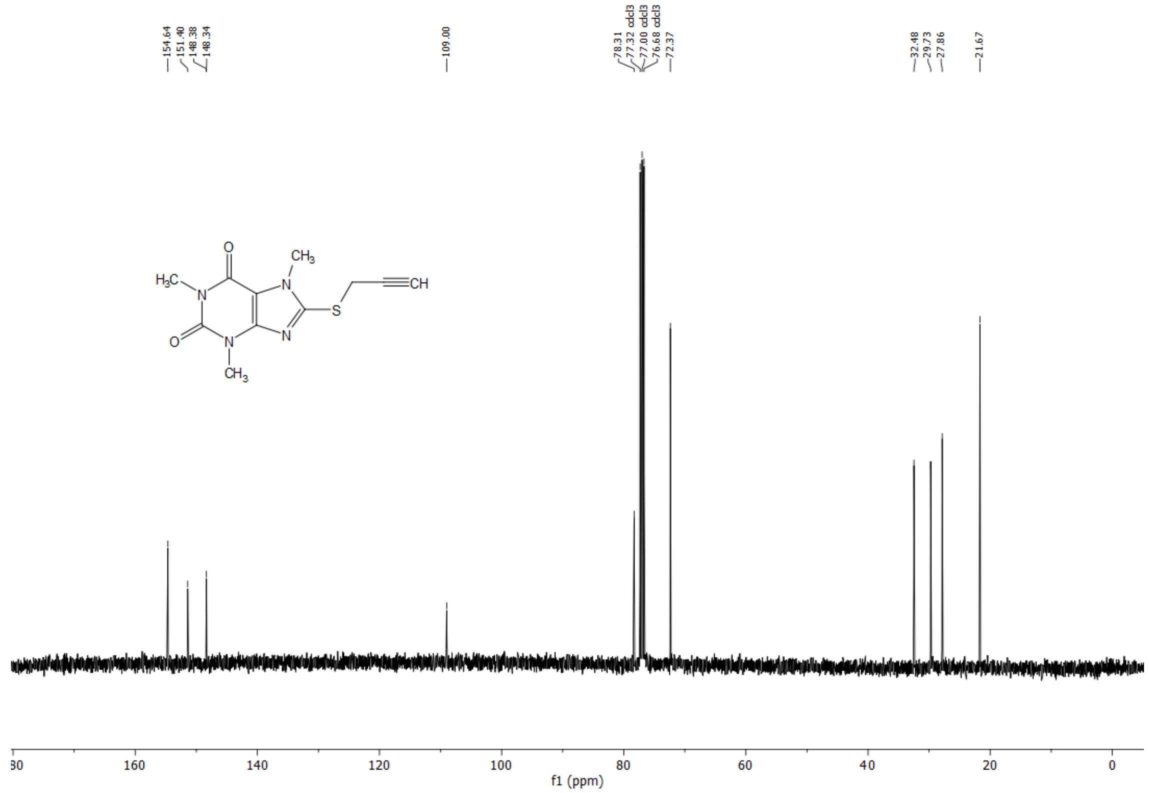

Fig. S1b ^{13}CNMR spectrum of compound **3**

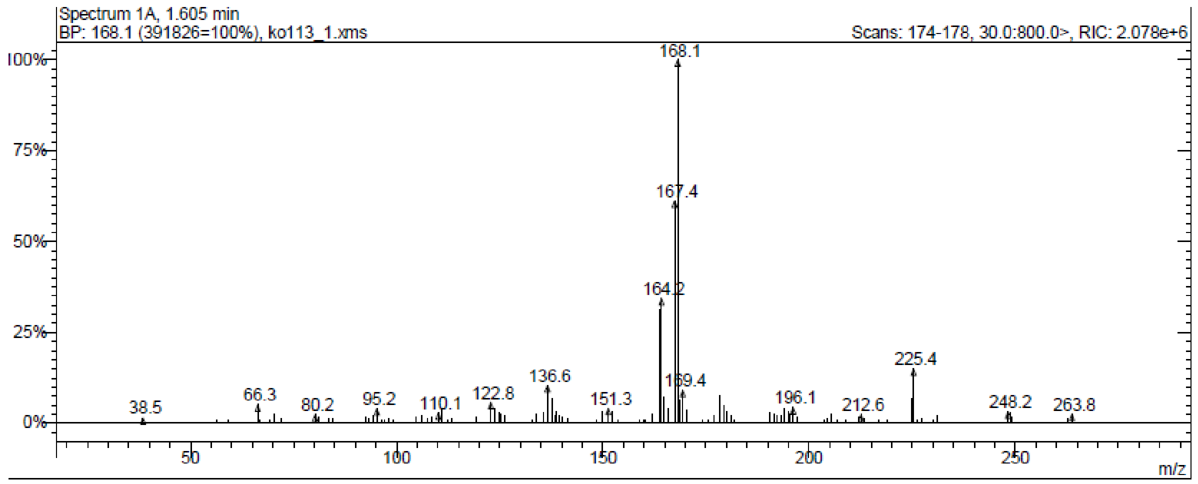

Fig. S1c EI-MS spectrum of compound **3**

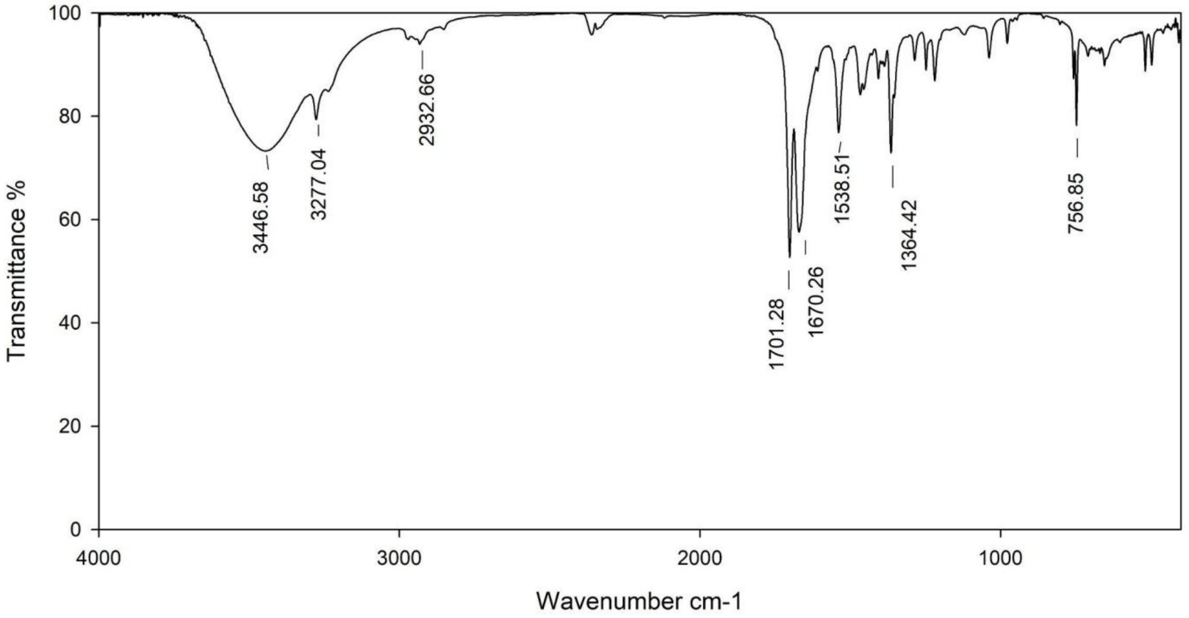

Fig.S1d FT-IR spectrum of compound **3**

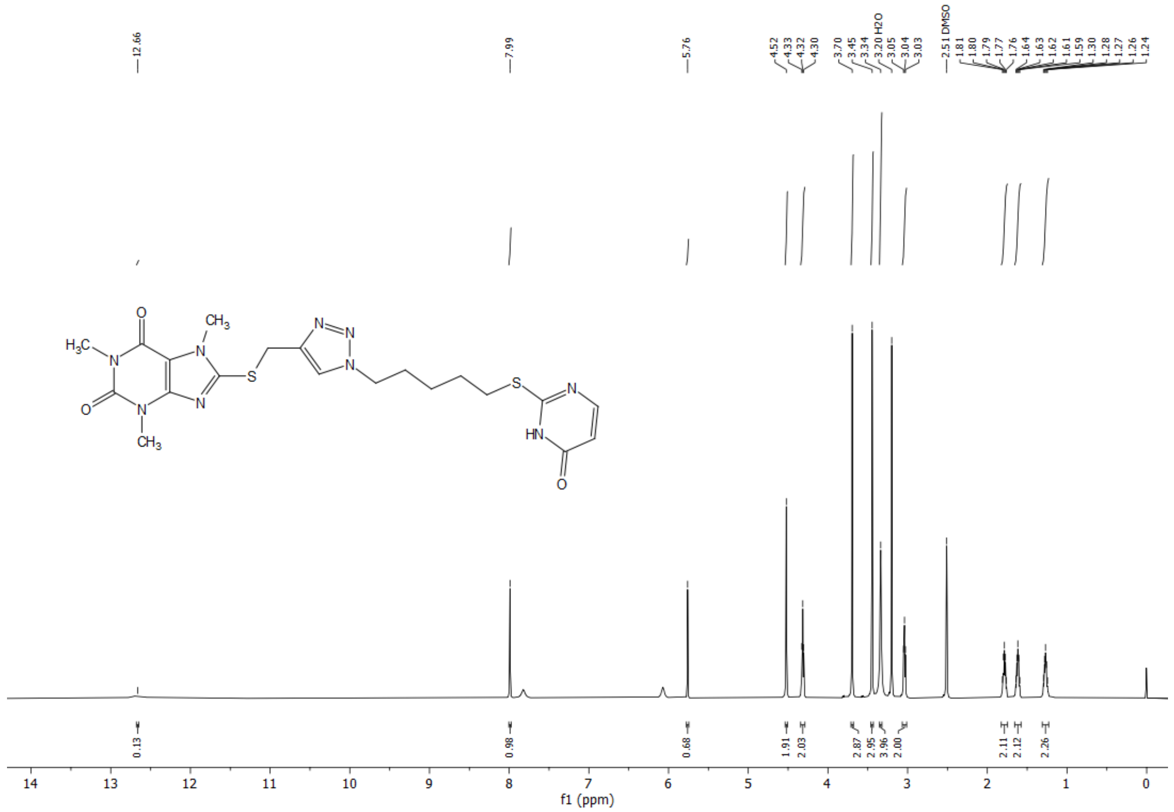

Fig. S2a ^{1}HNMR spectrum of compound **9a**

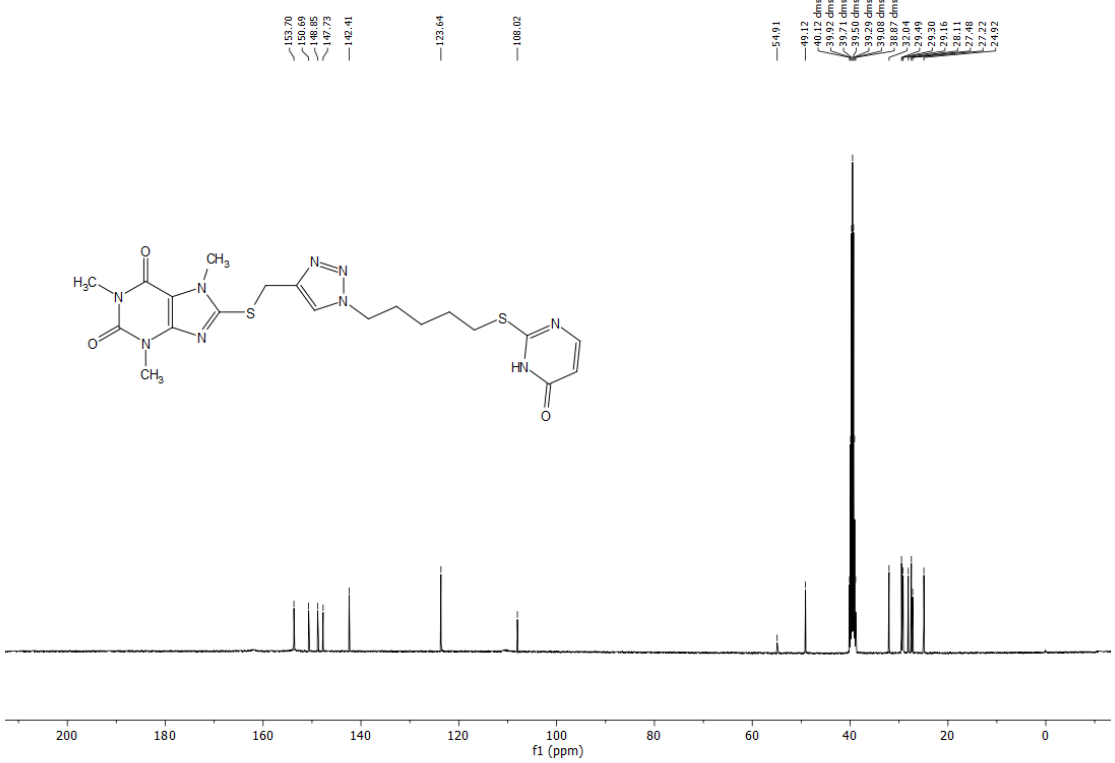

Fig. S2b ^{13}CNMR spectrum of compound **9a**

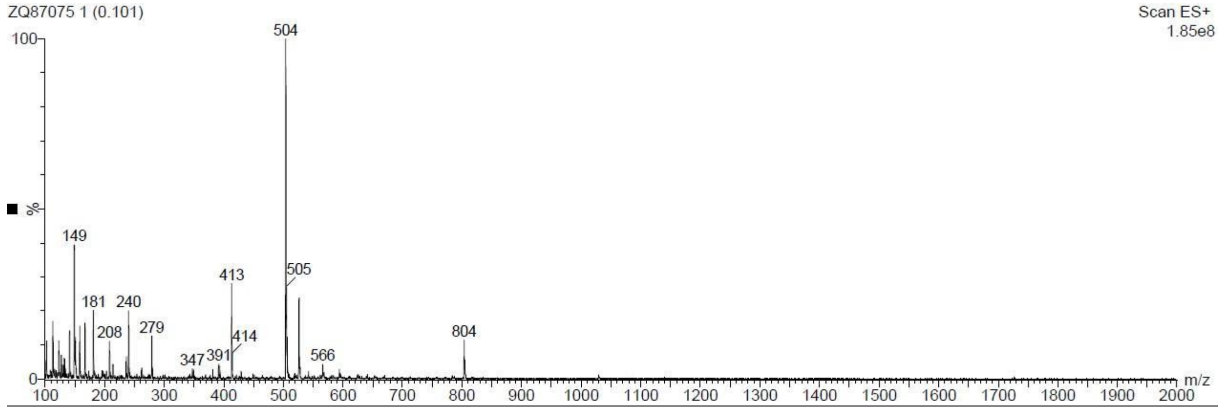

Fig. S2c ESI-MS spectrum of compound **9a**

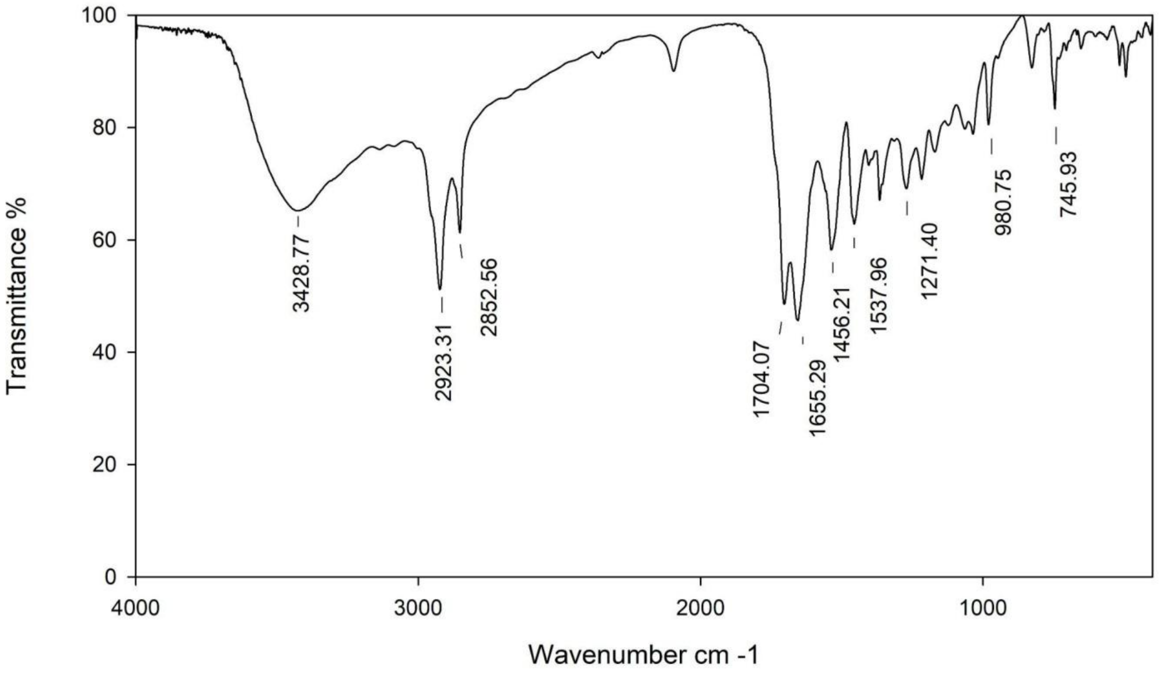

Fig. S2d FT-IR spectrum of compound **9a**

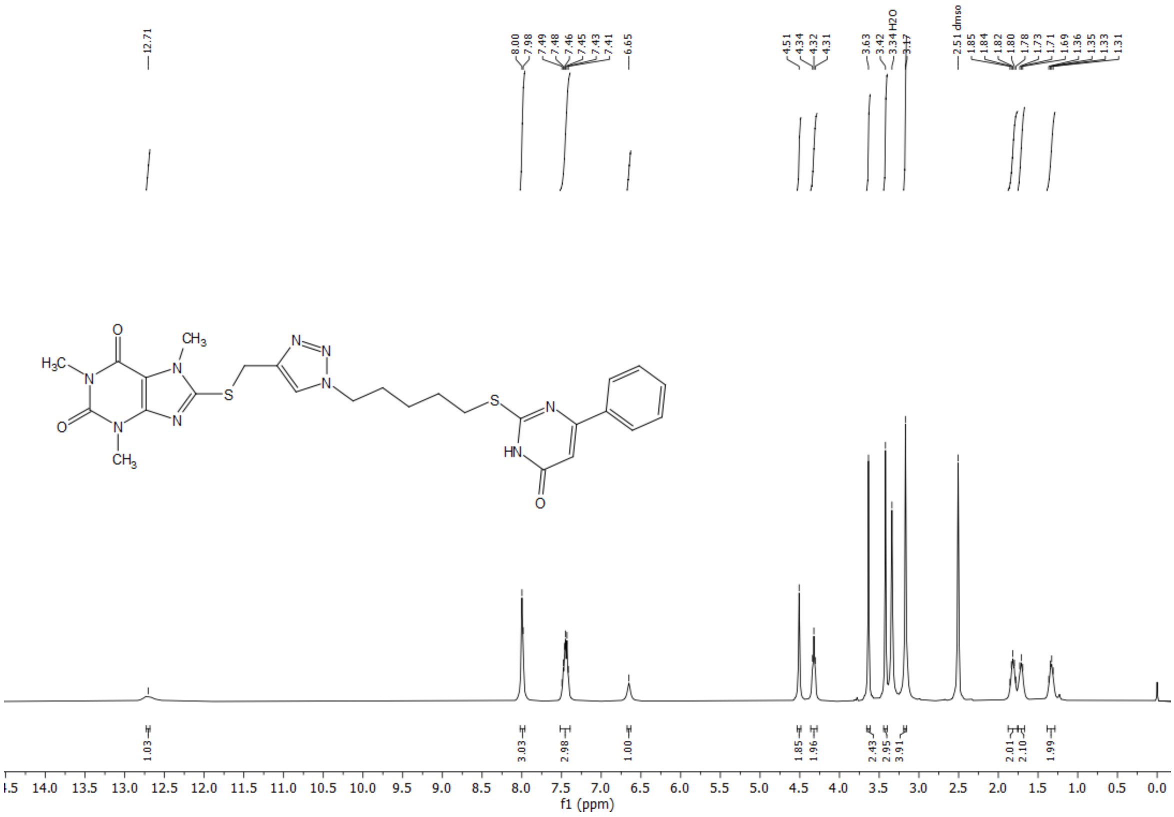

Fig. S3a ^{1}HNMR spectrum of compound **9b**

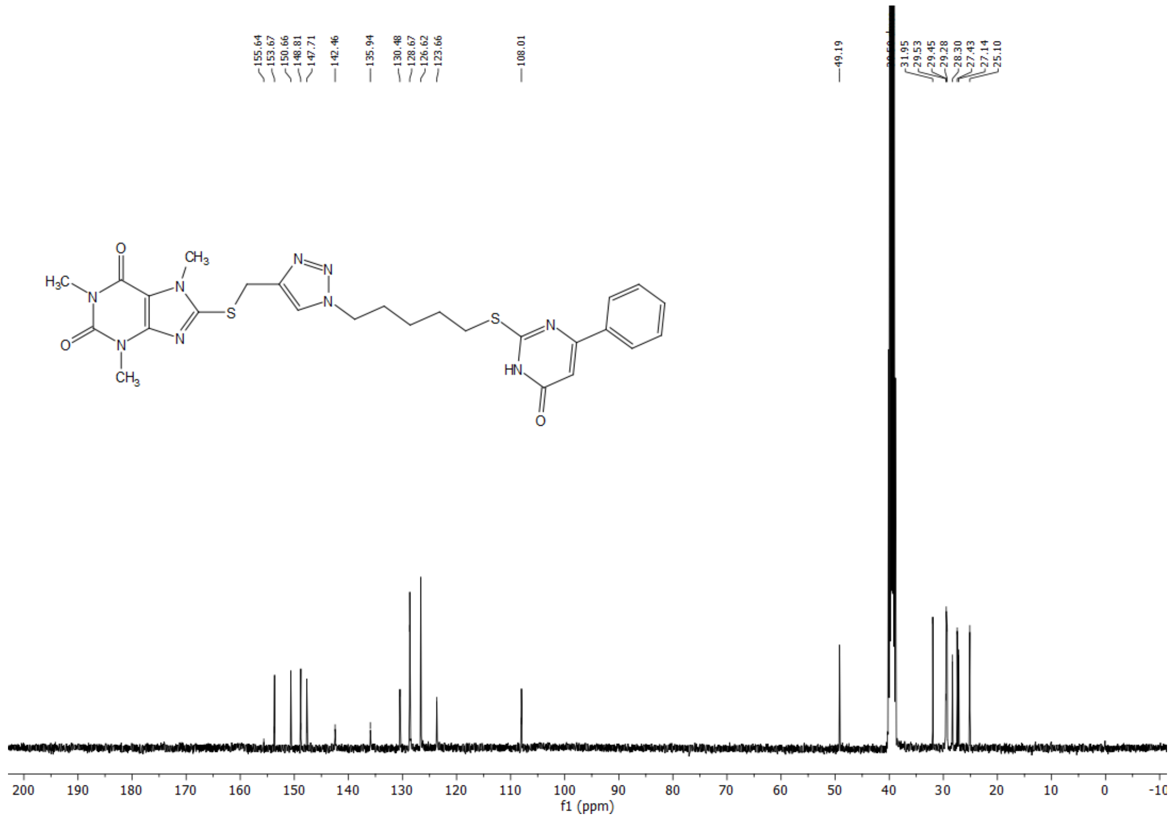

Fig. S3b ^{13}CNMR spectrum of compound **9b**

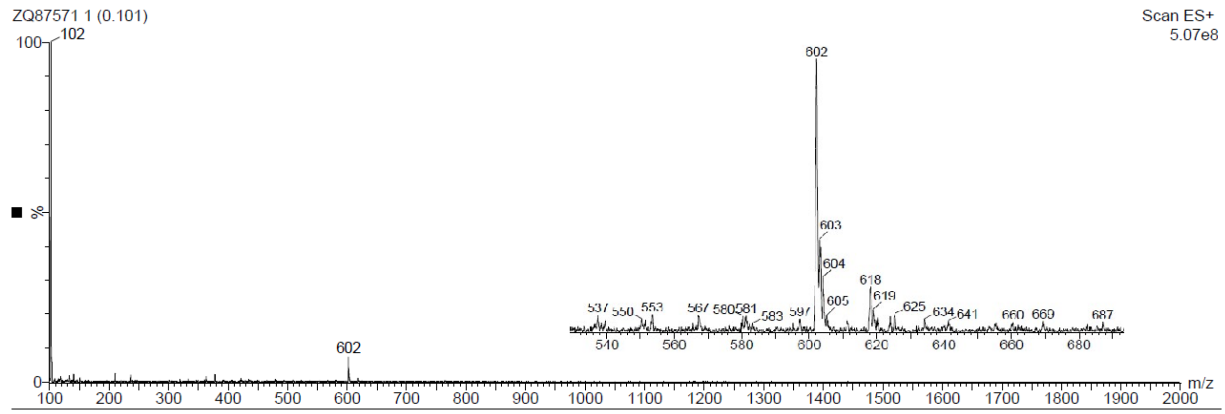

Fig. S3c ESI-MS spectrum of compound **9b**

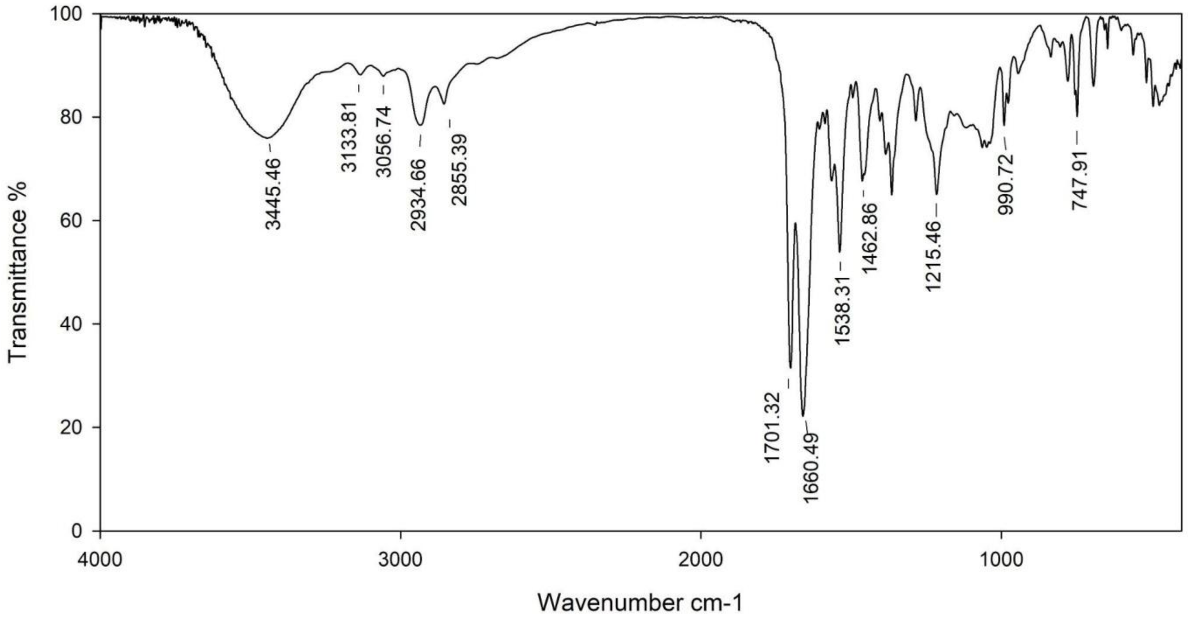

Fig. S3d FT-IR spectrum of compound **9b**

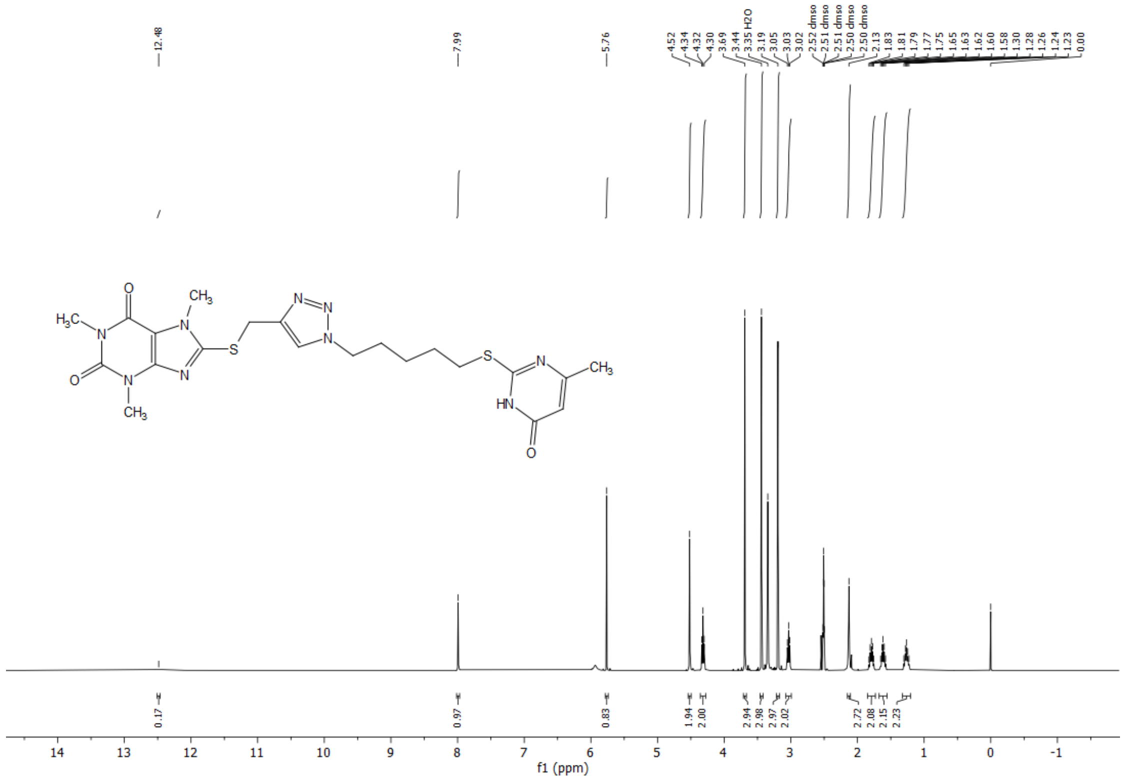

Fig. S4a ^{1}HNMR spectrum of compound **9c**

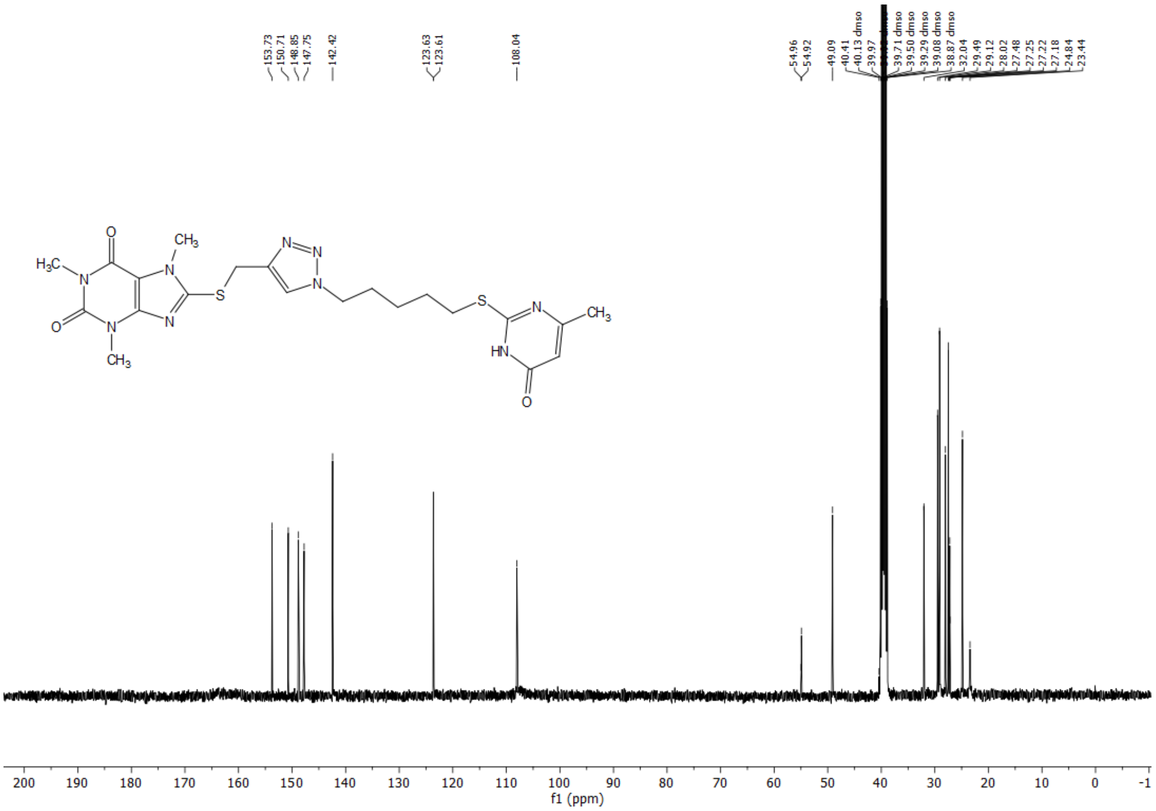

Fig. S4b ^{13}CNMR spectrum of compound **9c**

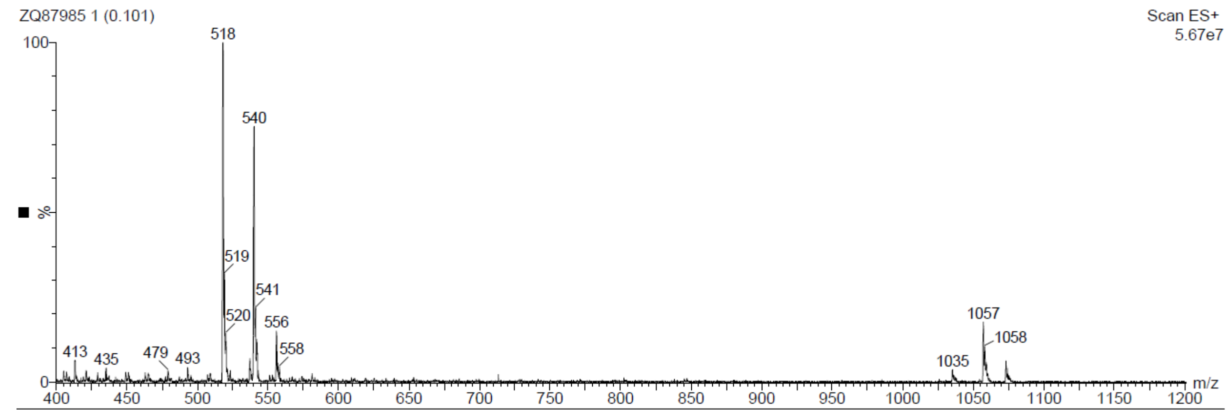

Fig. S4c ESI-MS spectrum of compound **9c**

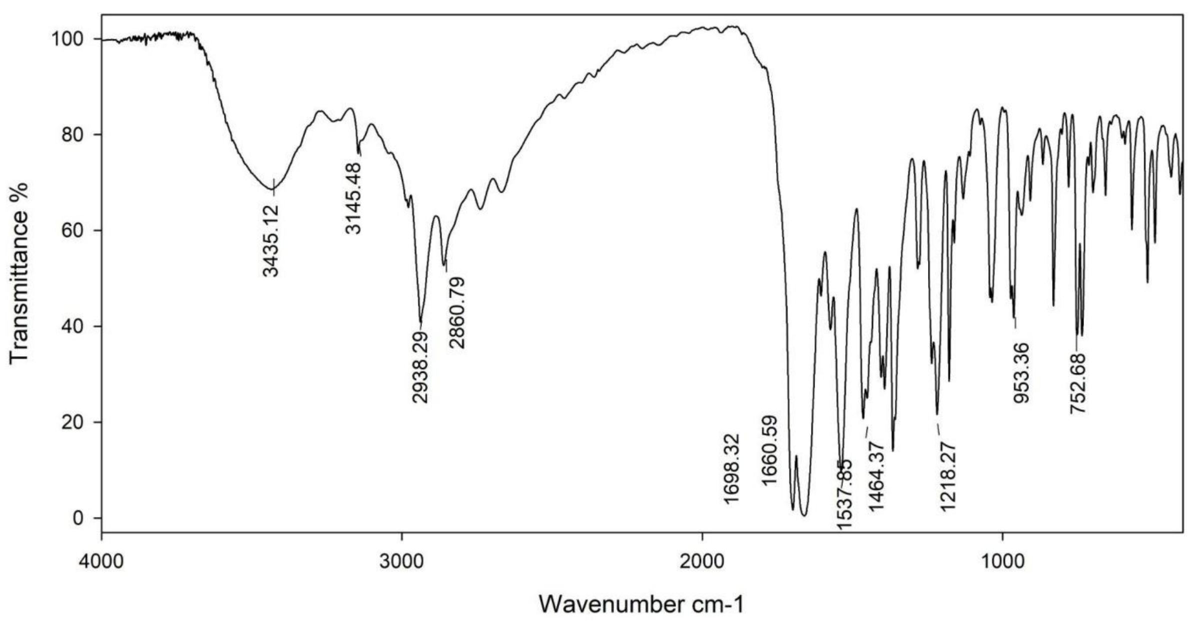

Fig. S4d FT-IR spectrum of compound **9c**

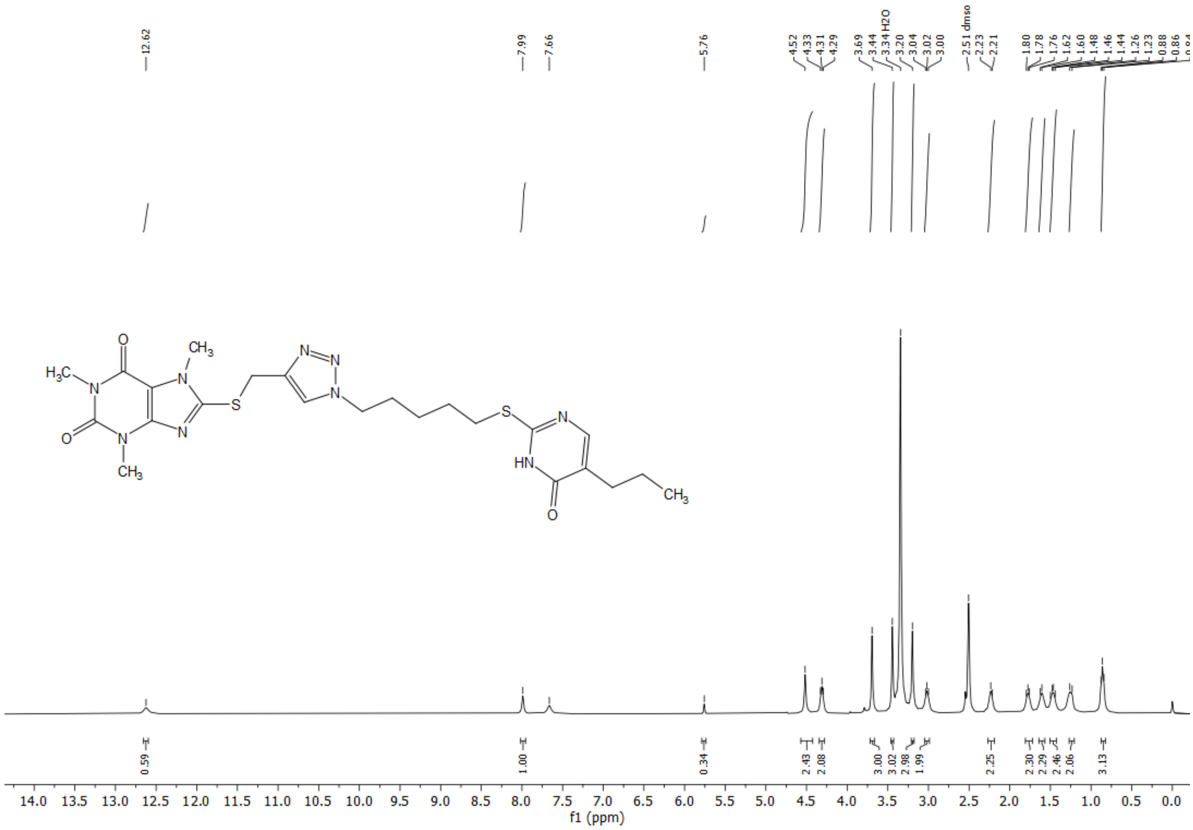

Fig. S5a ¹HNMR spectrum of compound **9d**

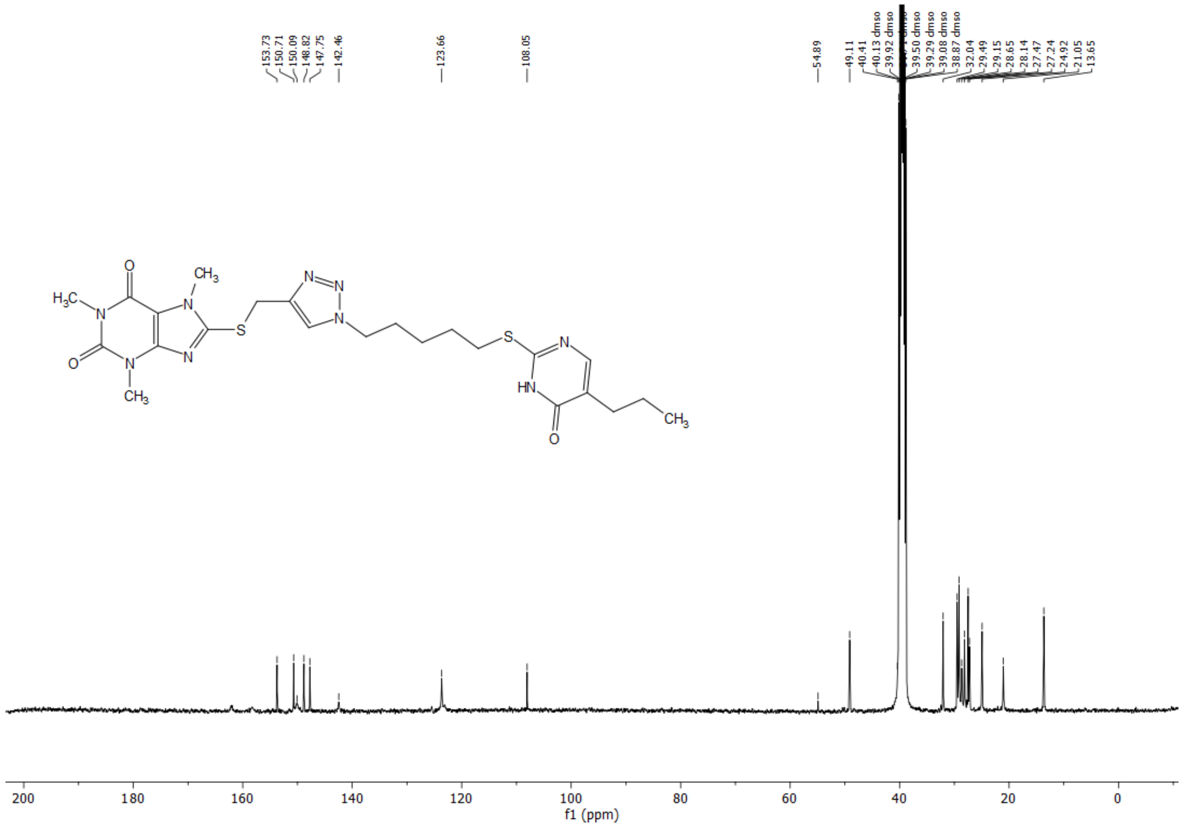

Fig. S5b ¹³CNMR spectrum of compound **9d**

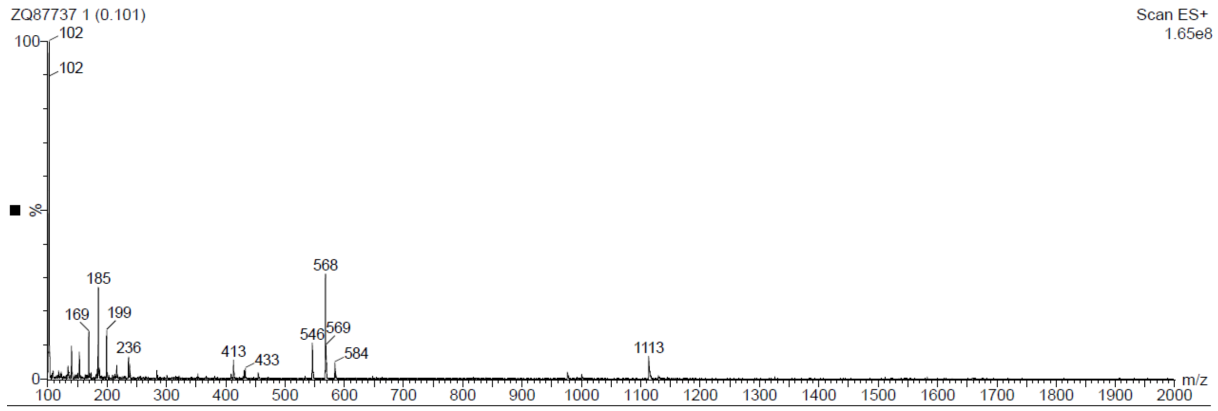

Fig. S5c ESI-MS spectrum of compound **9d**

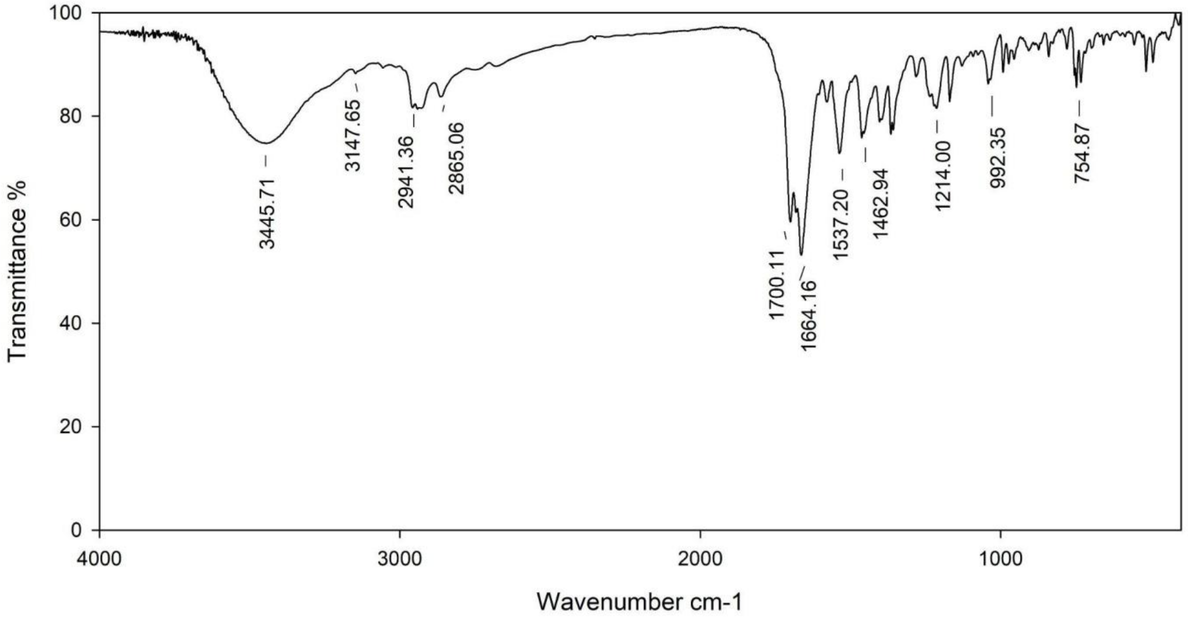

Fig. S5d FT-IR spectrum of compound **9d**

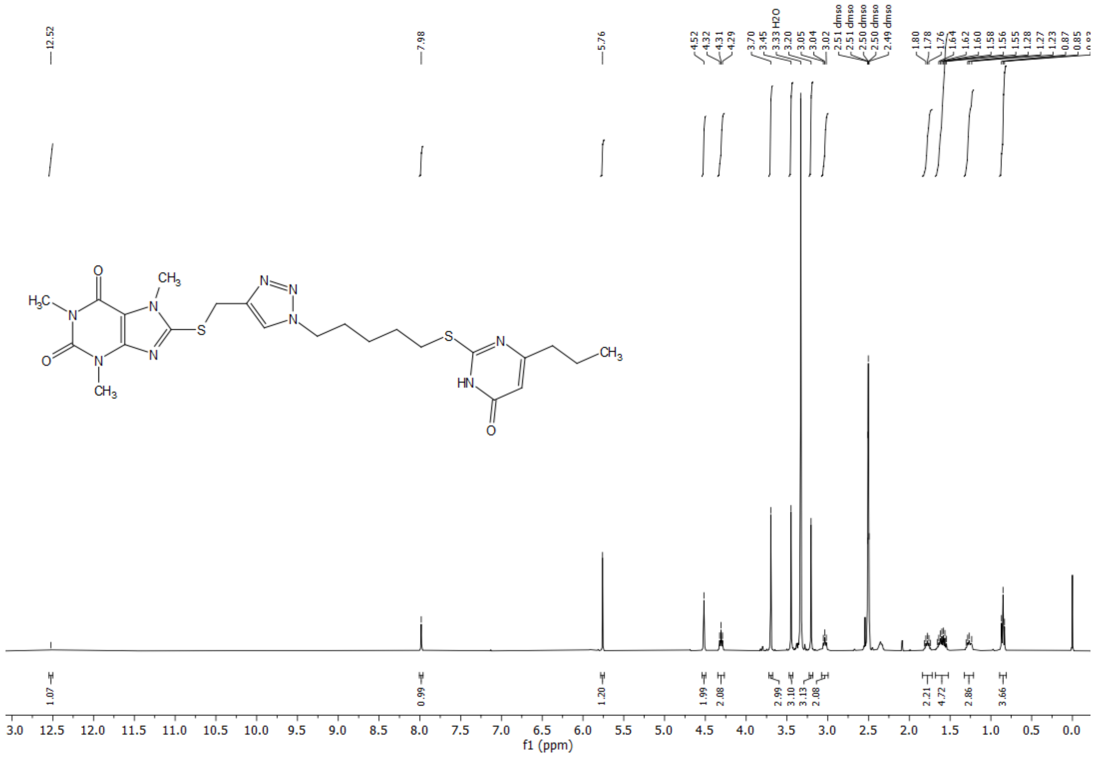

Fig. S6a ^{1}HNMR spectrum of compound **9e**

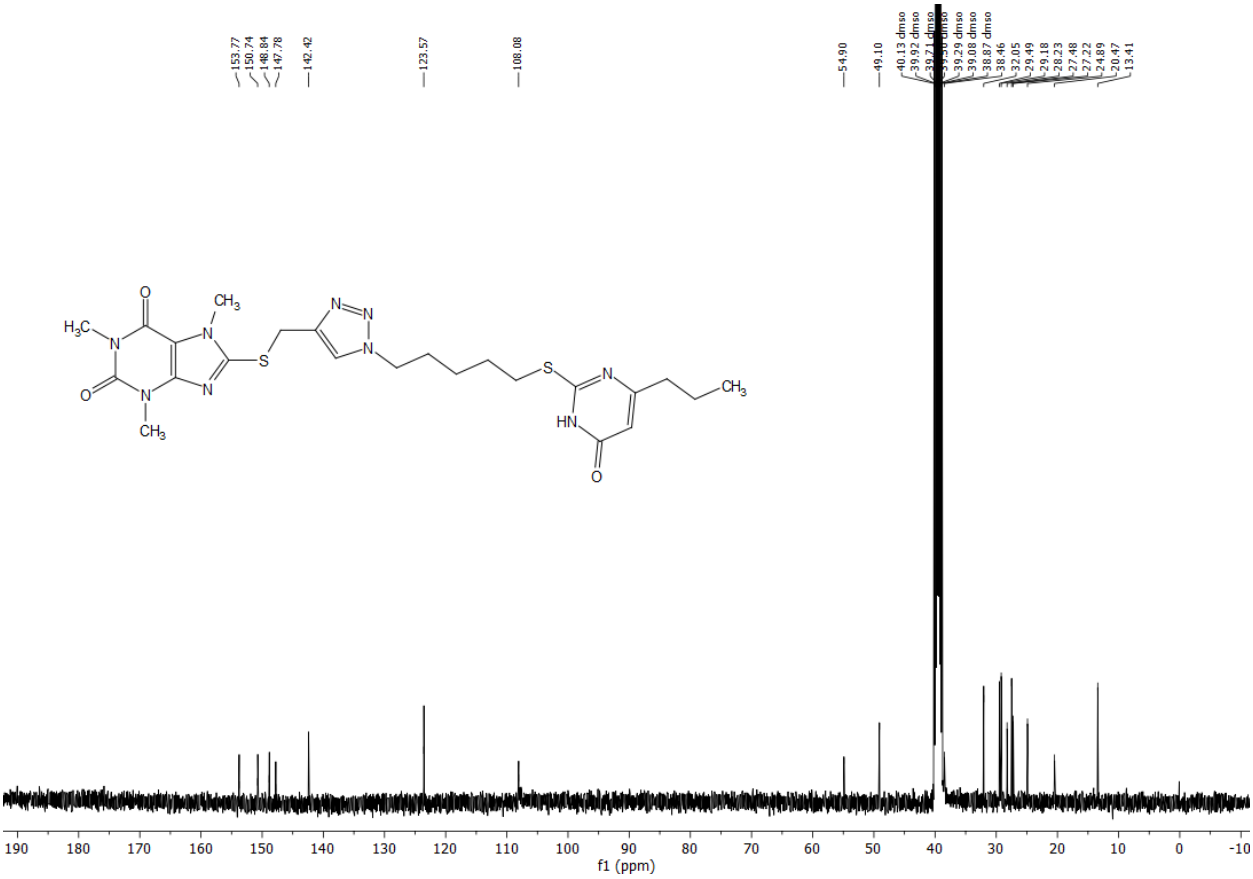

Fig. S6b ^{13}CNMR spectrum of compound **9e**

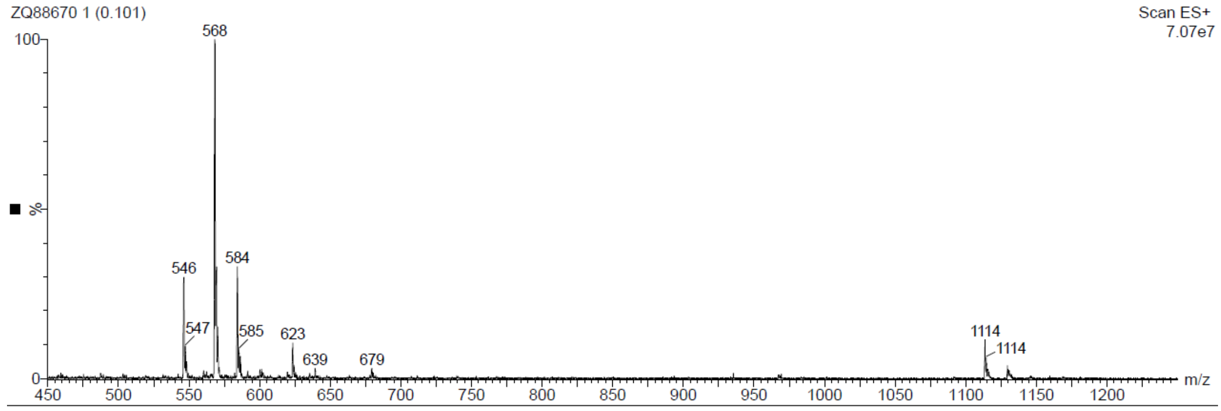

Fig. S6c ESI-MS spectrum of compound **9e**

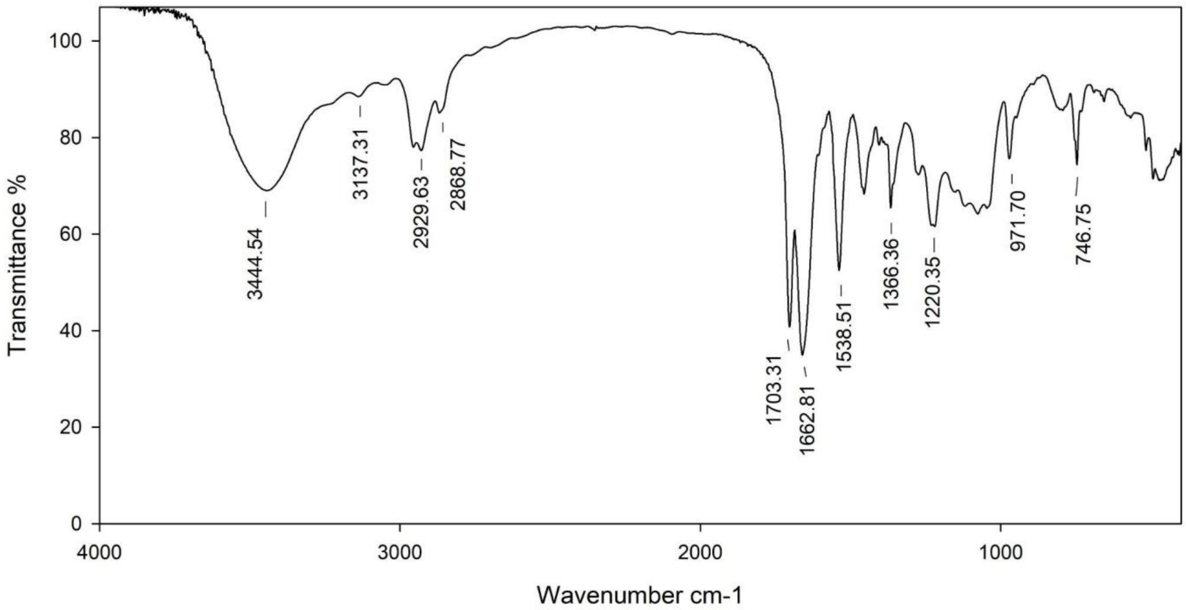

Fig. S6d FT-IR spectrum of compound **9e**

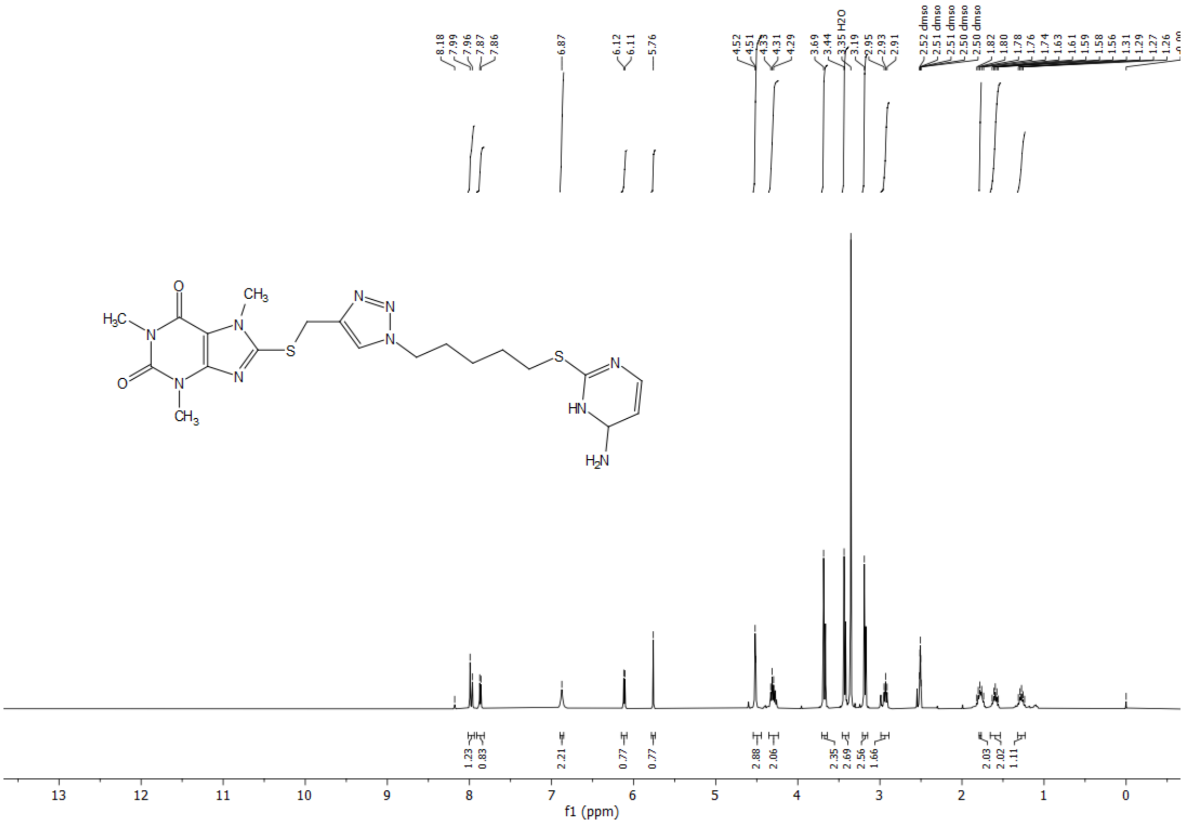

Fig. S7a ^{1}HNMR spectrum of compound **9f**

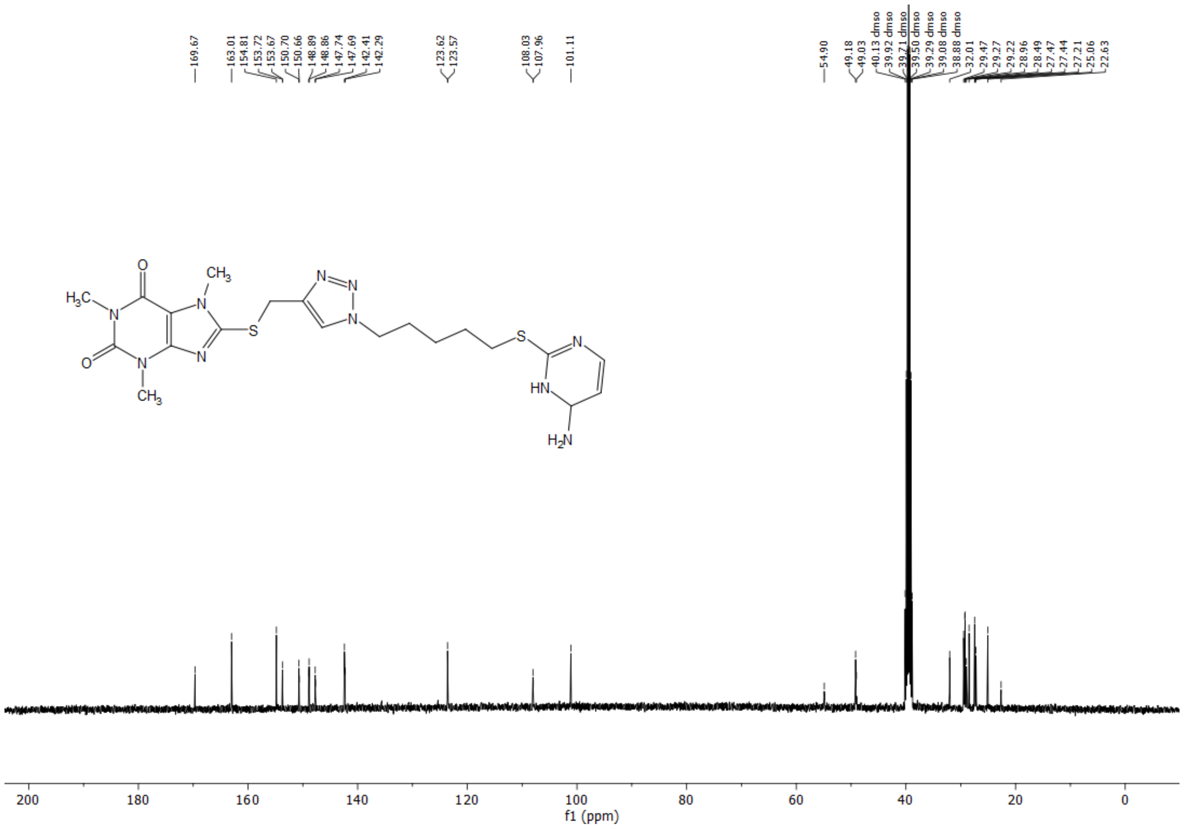

Fig. S7b ^{13}CNMR spectrum of compound **9f**

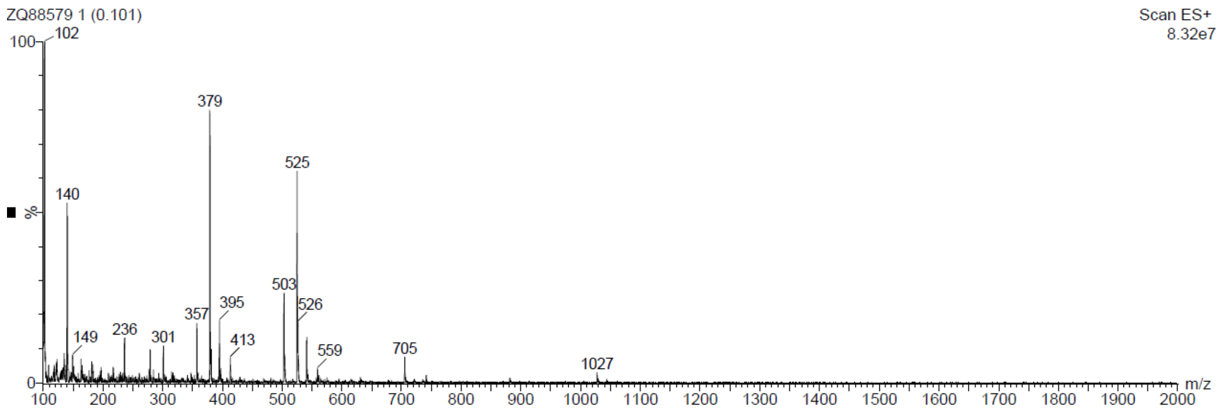

Fig. S7c ESI-MS spectrum of compound **9f**

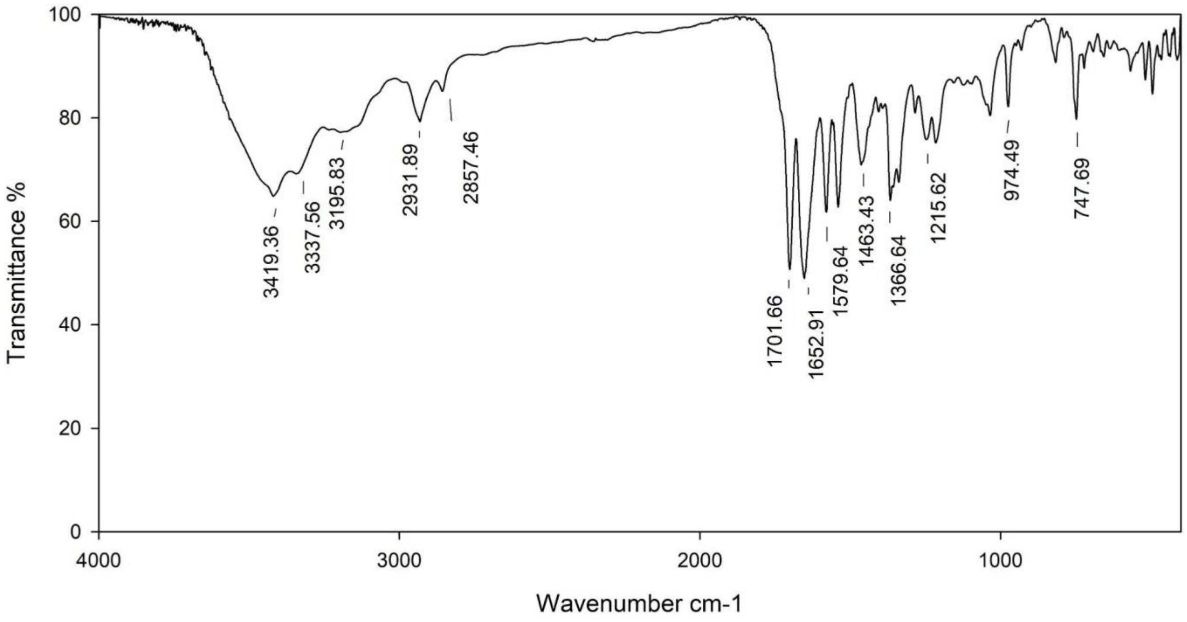

Fig. S7d FT-IR spectrum of compound **9f**

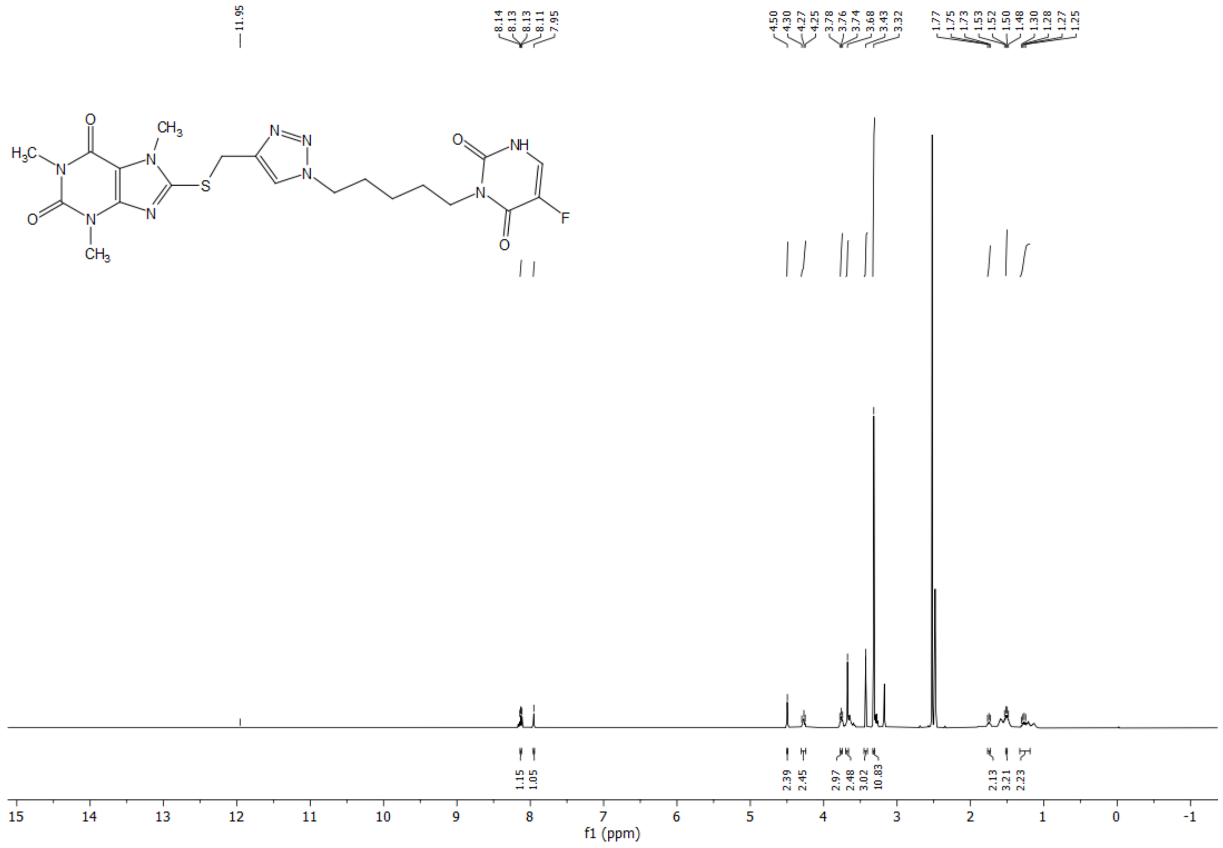

Fig. S8a ^{1}HNMR spectrum of compound **10**

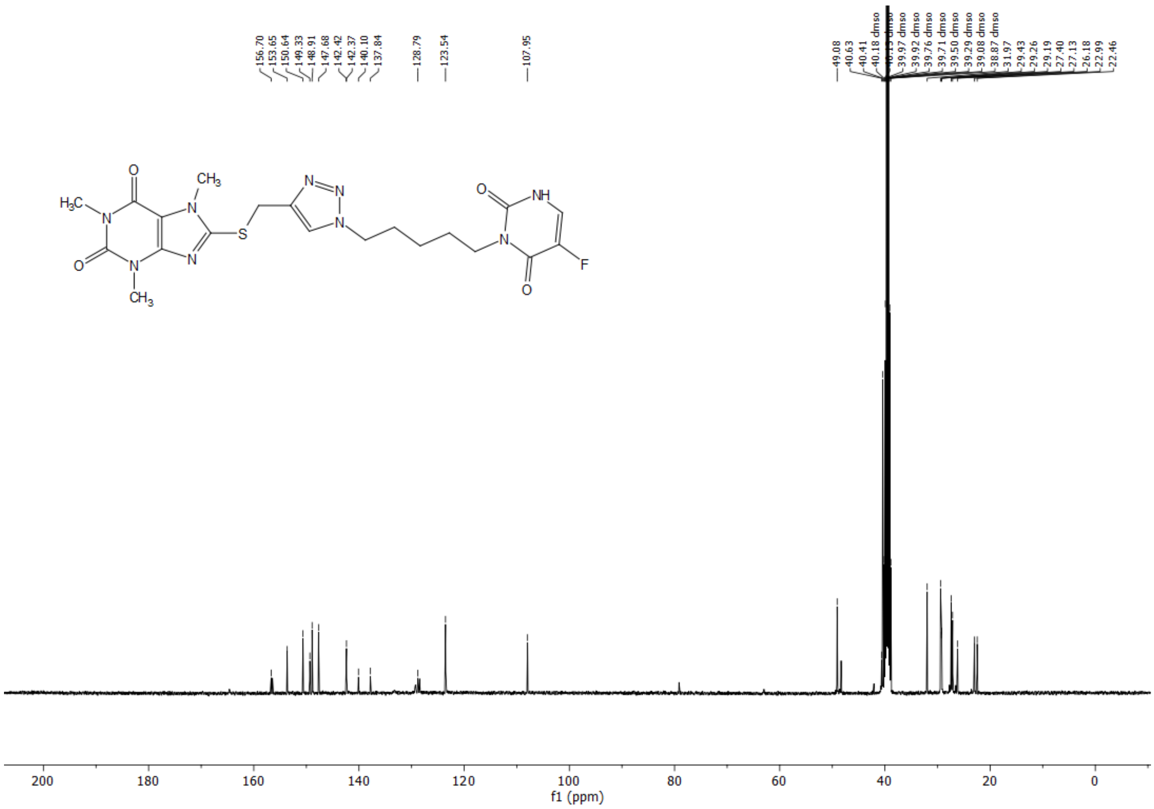

Fig. S8b ^{13}CNMR spectrum of compound **10**

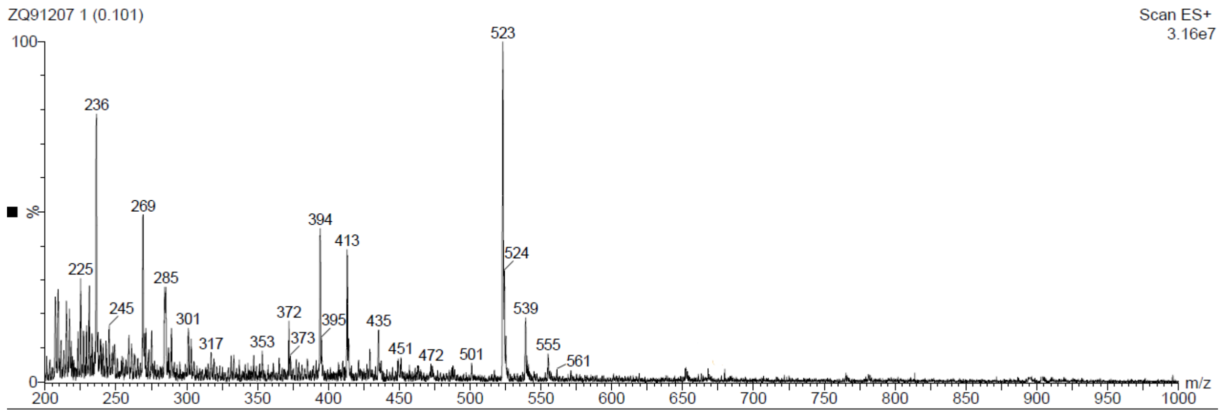

Fig. S8c ESI-MS spectrum of compound **10**

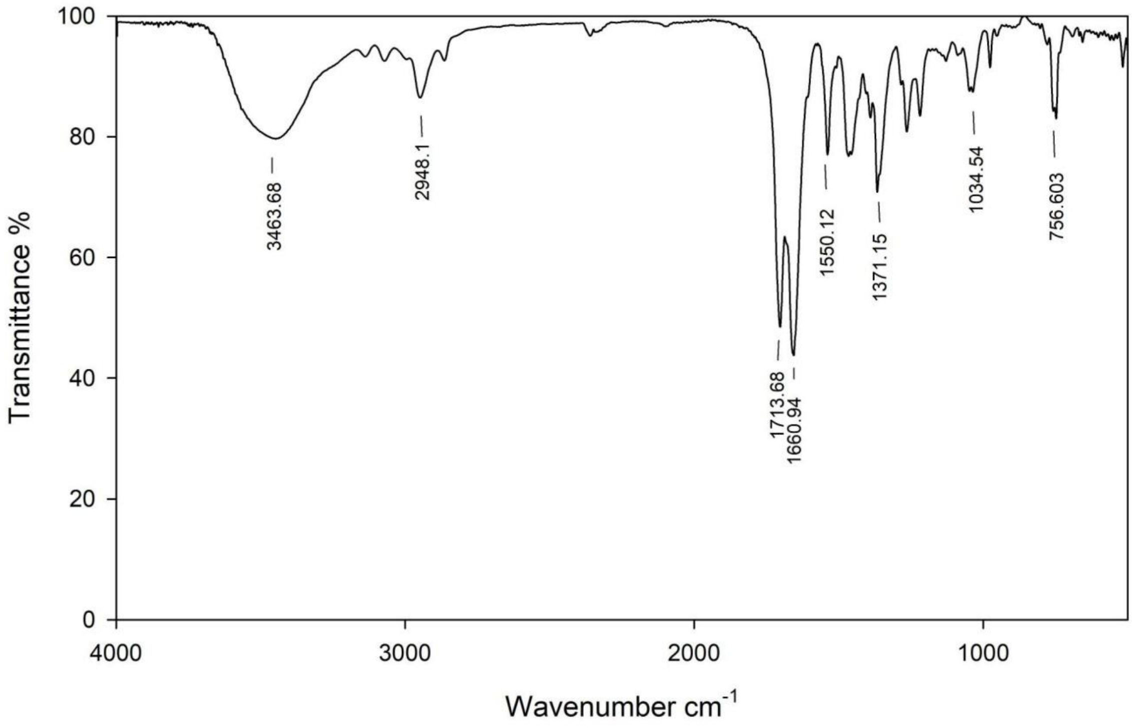

Fig. S8d FT-IR spectrum of compound **10**

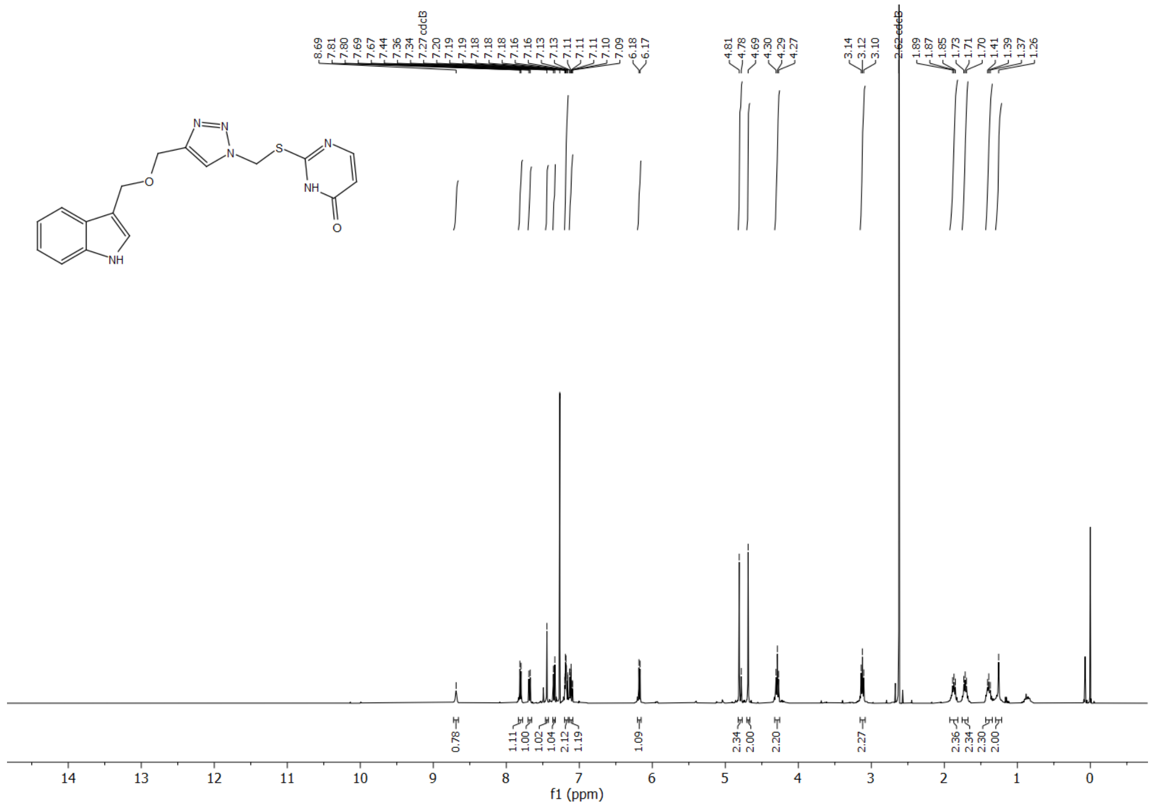

Fig. S9a ¹HNMR spectrum of compound **11a**

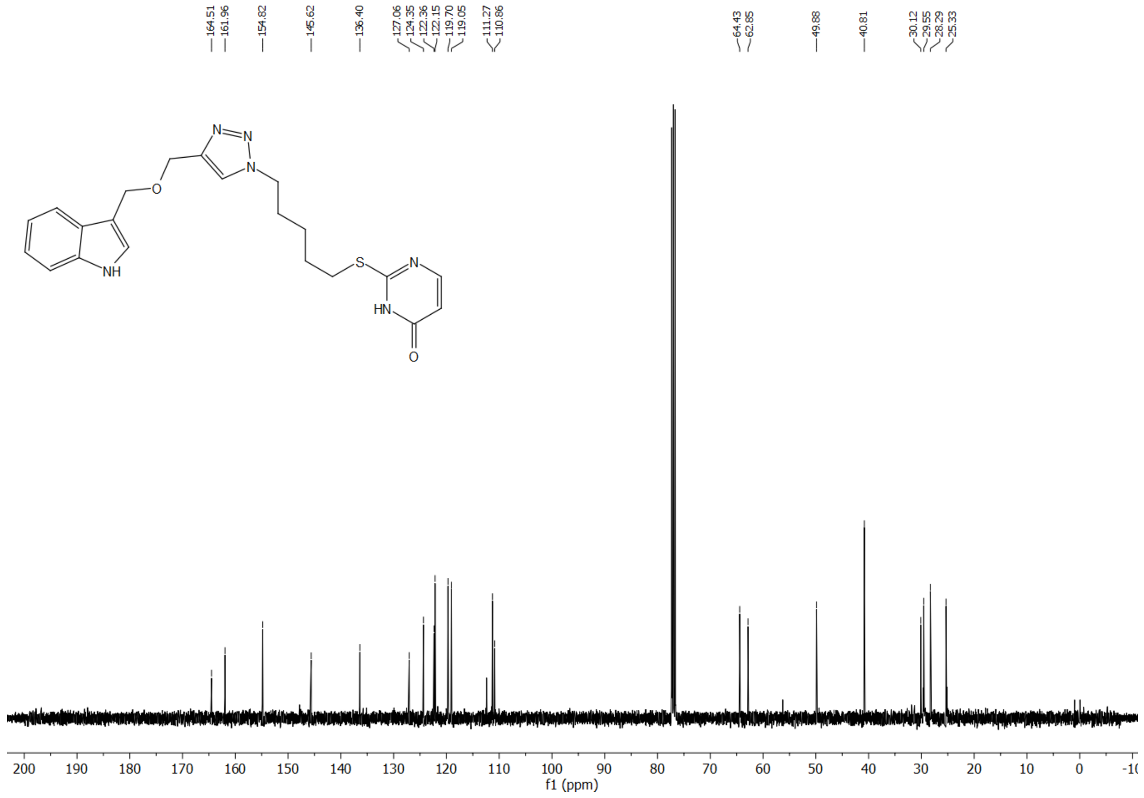

Fig. S9b ¹³CNMR spectrum of compound **11a**

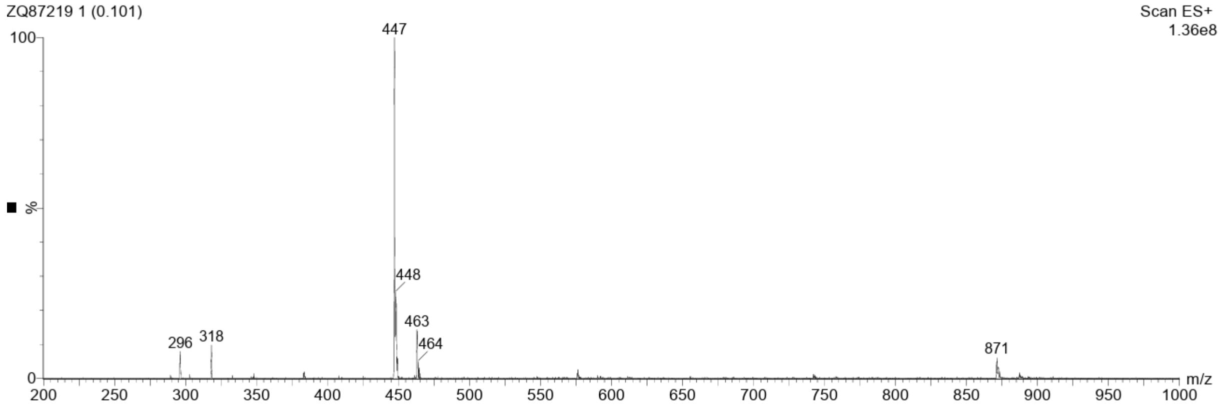

Fig. S9c ESI-MS spectrum of compound **11a**

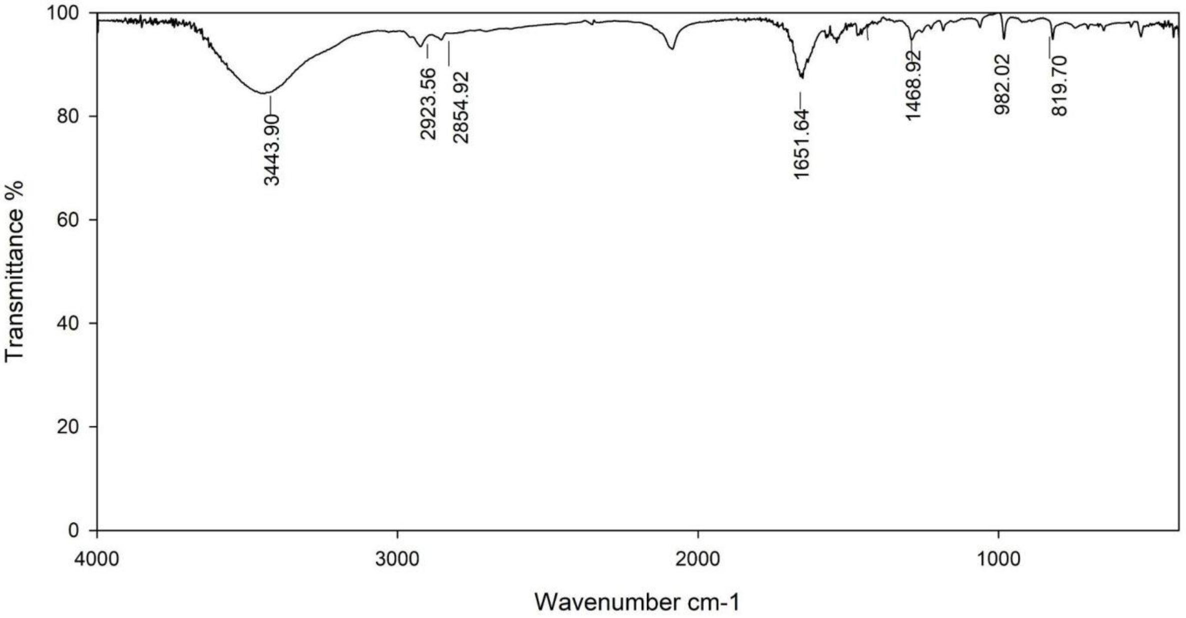

Fig. S9d FT-IR spectrum of compound **11a**

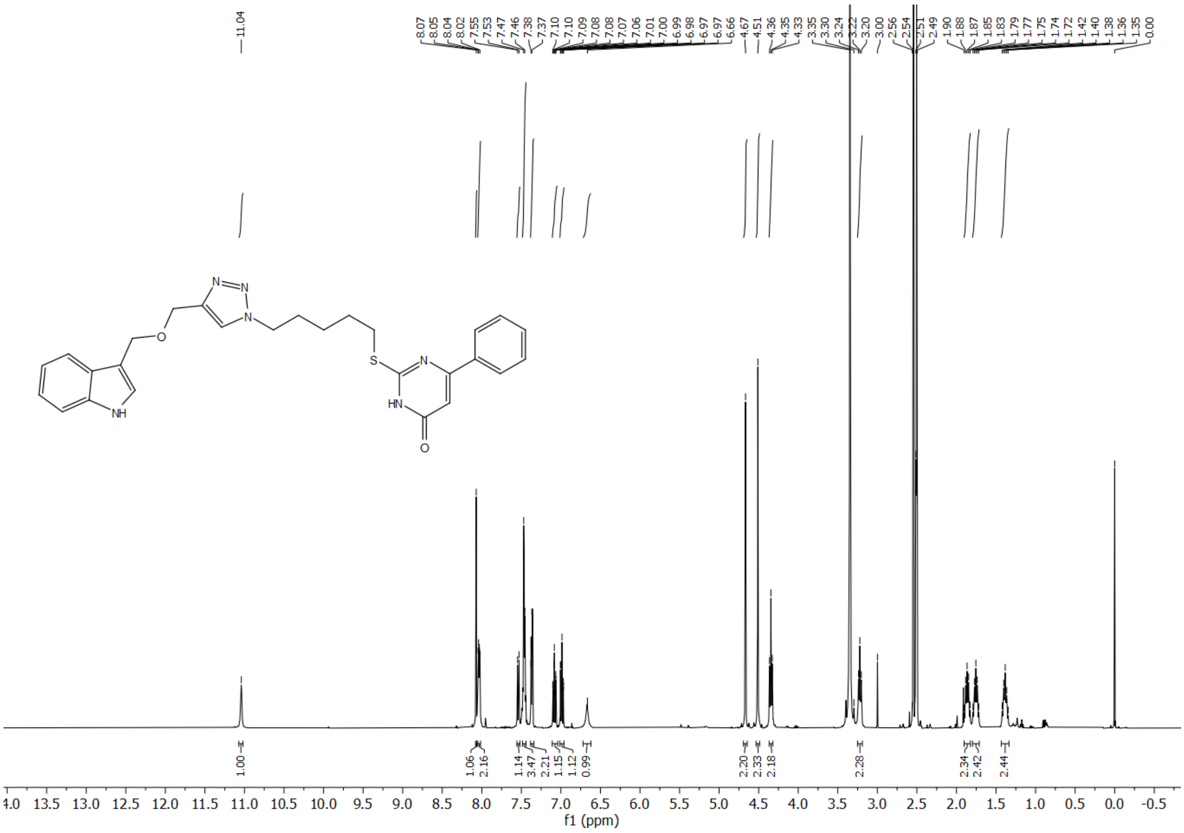

Fig. S10a ^{1}HNMR spectrum of compound **11b**

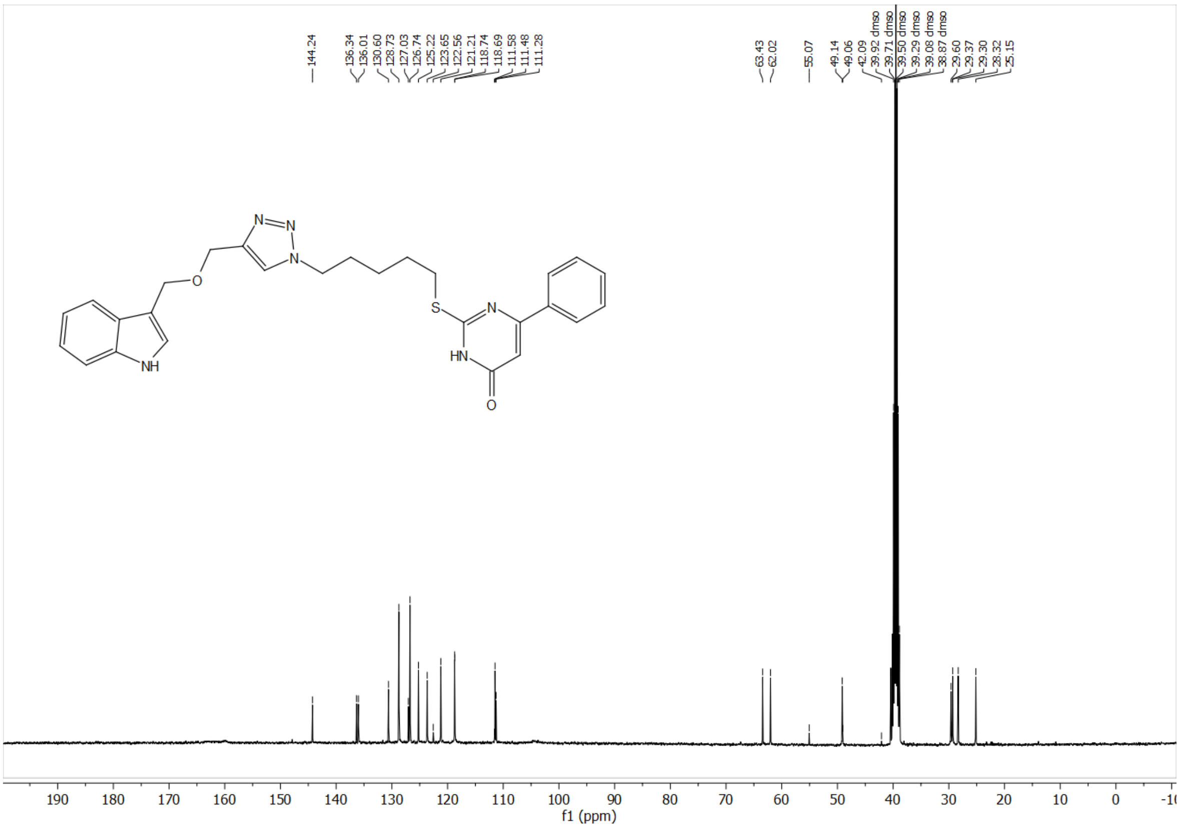

Fig. S10b ^{13}CNMR spectrum of compound **11b**

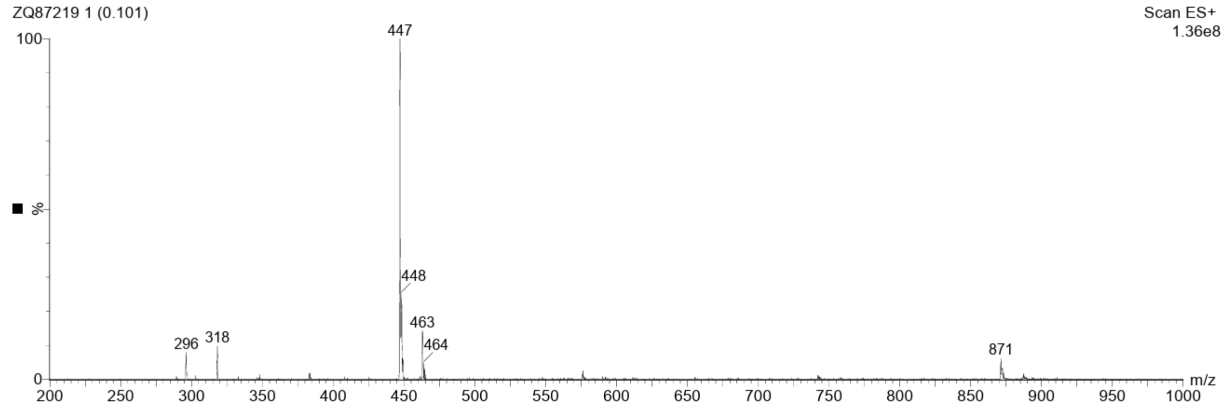

Fig. S10c ESI-MS spectrum of compound **11b**

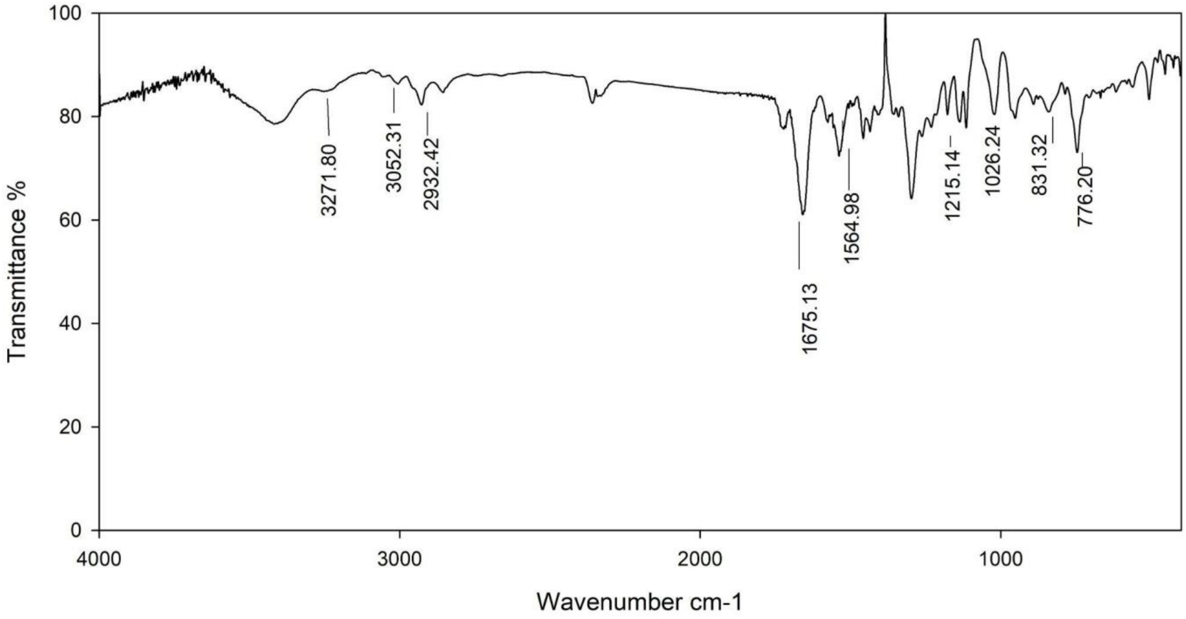

Fig. S10d FT-IR spectrum of compound **11b**

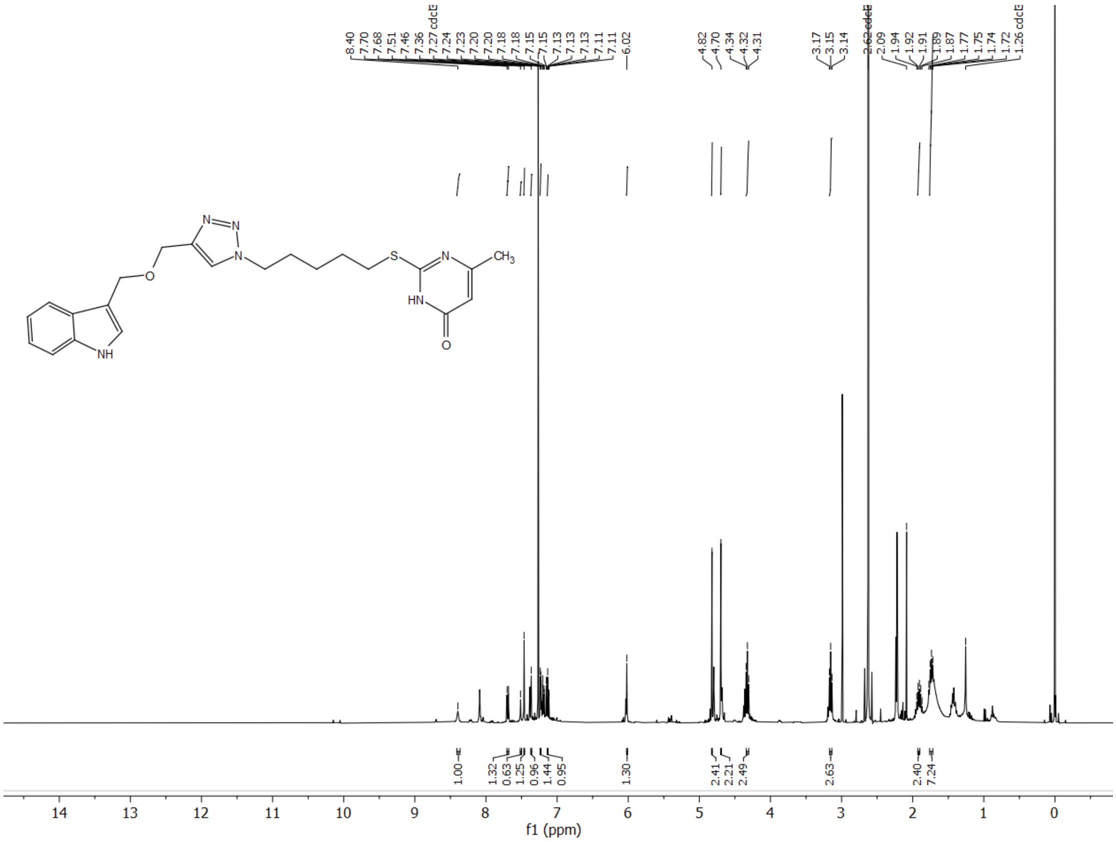

Fig. S11a ^{1}HNMR spectrum of compound **11c**

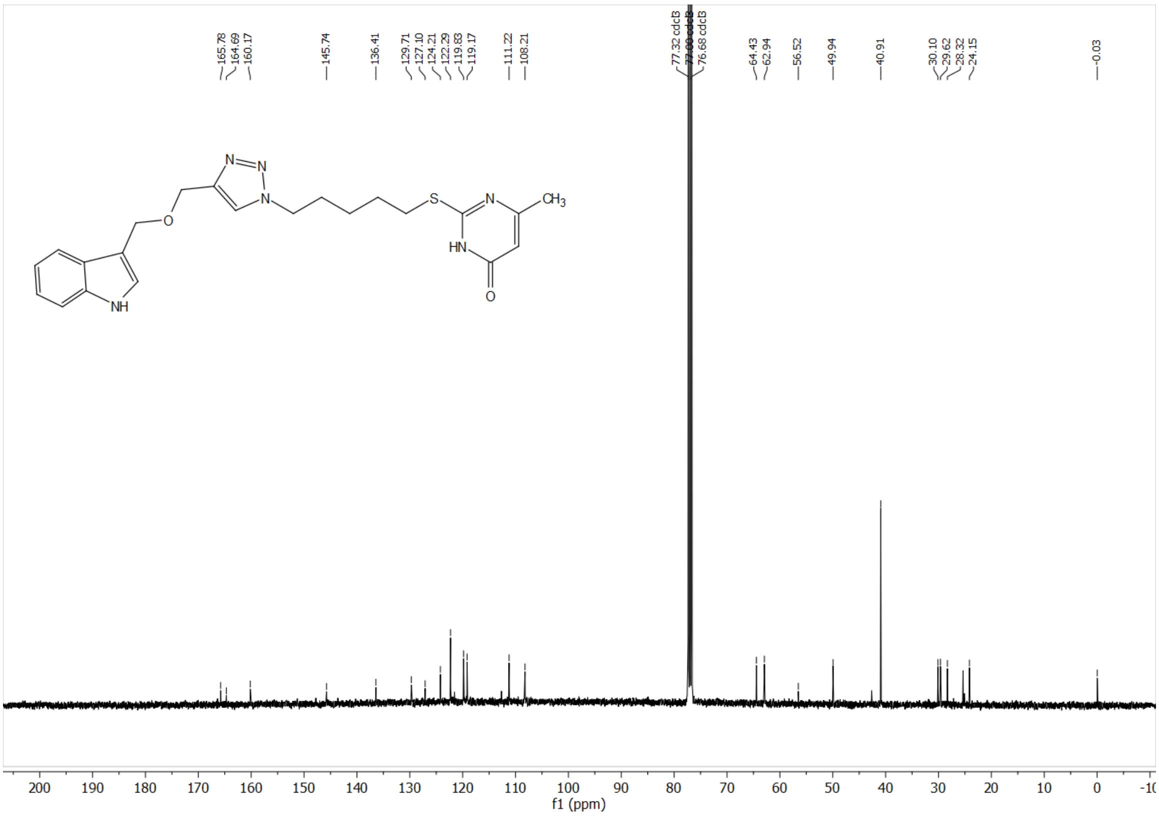

Fig. S11b ^{13}CNMR spectrum of compound **11c**

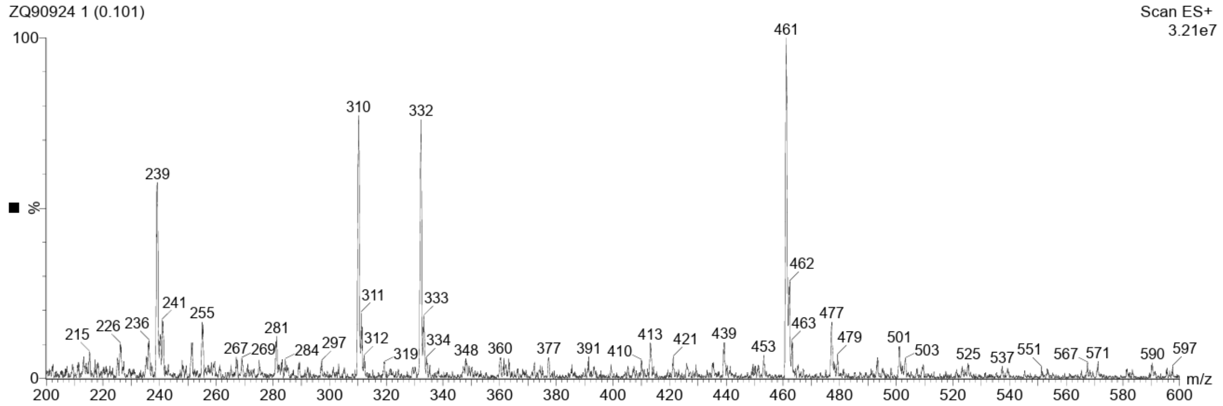

Fig. S11c ESI-MS spectrum of compound **11c**

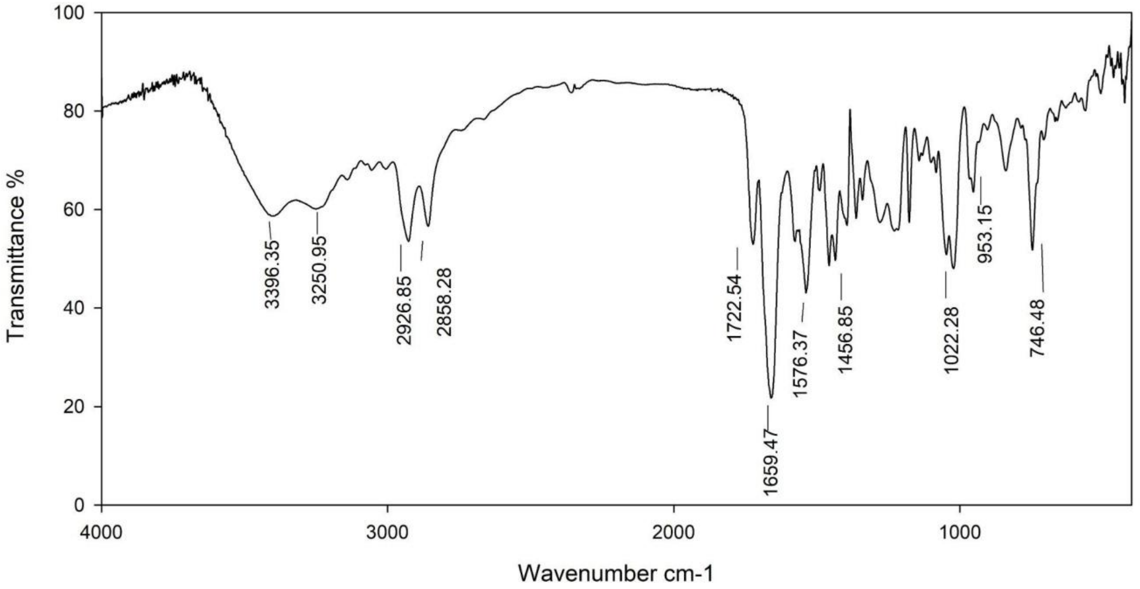

Fig. S11d FT-IR spectrum of compound **11c**

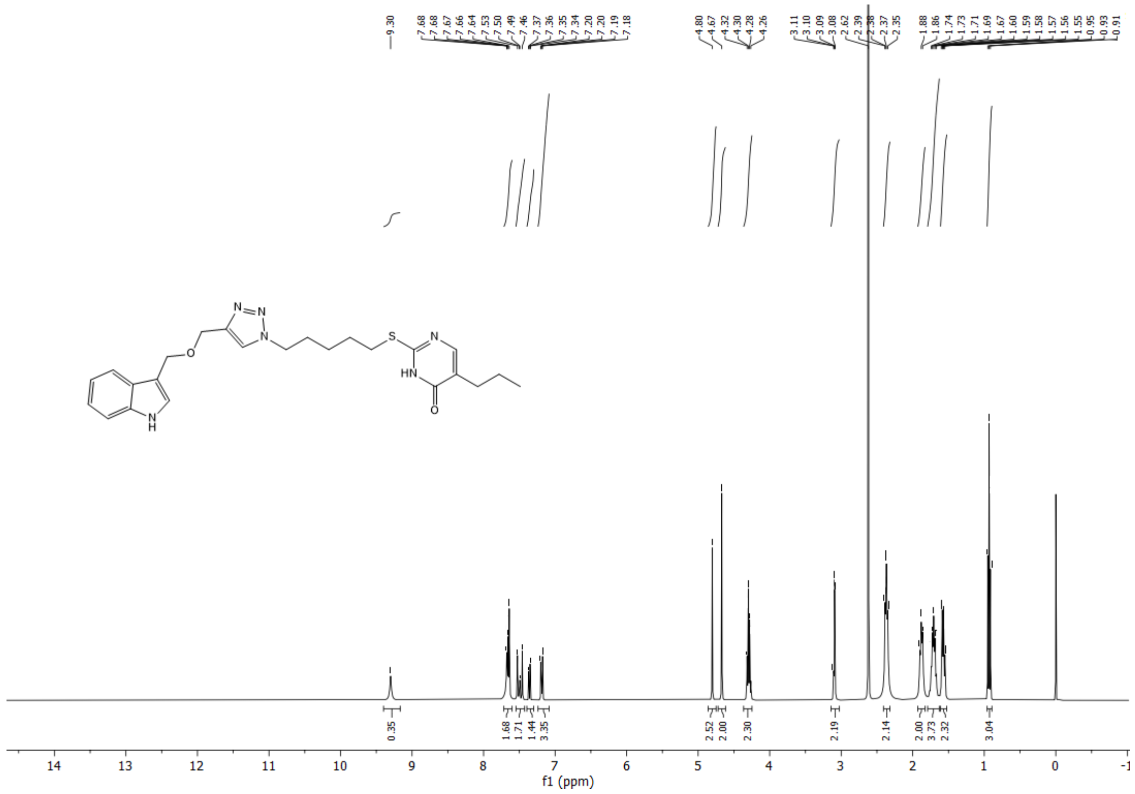

Fig. S12a ^{1}HNMR spectrum of compound **11d**

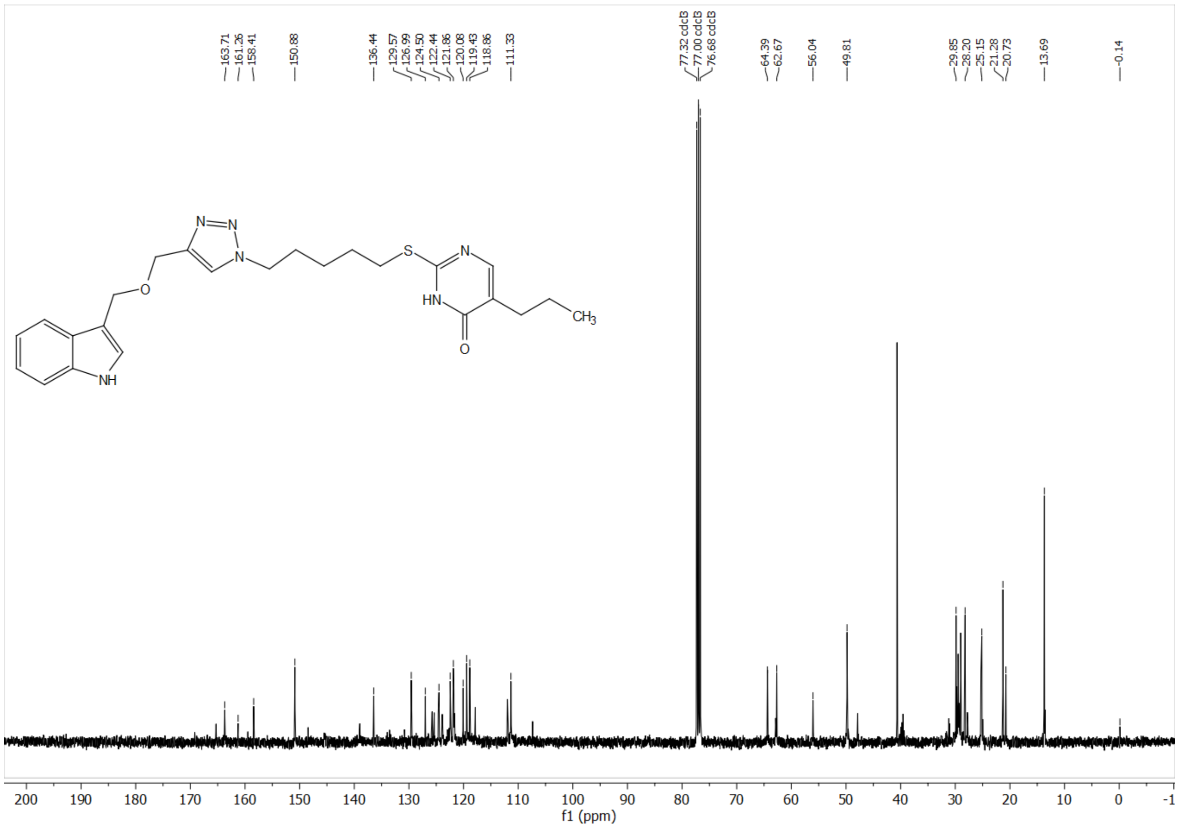

Fig. S12b ^{13}CNMR spectrum of compound **11d**

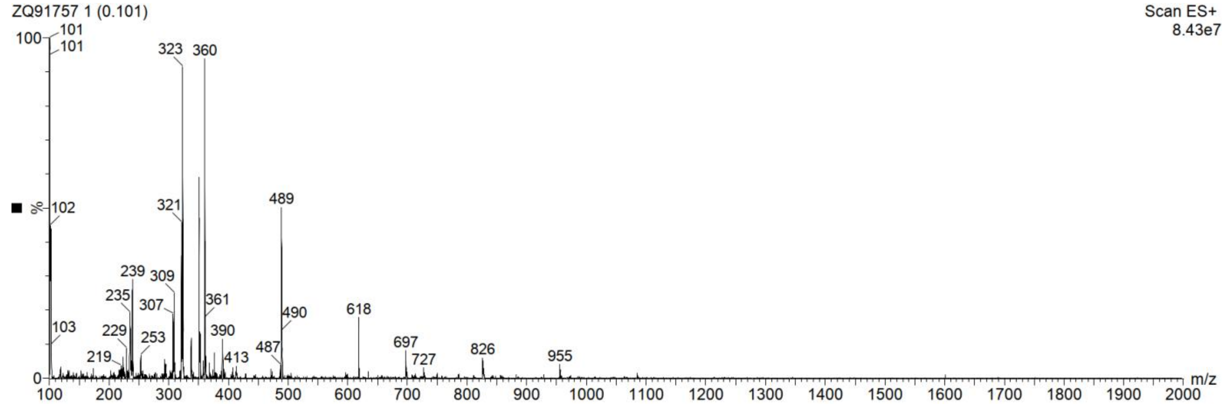

Fig. S12c ESI-MS spectrum of compound **11d**

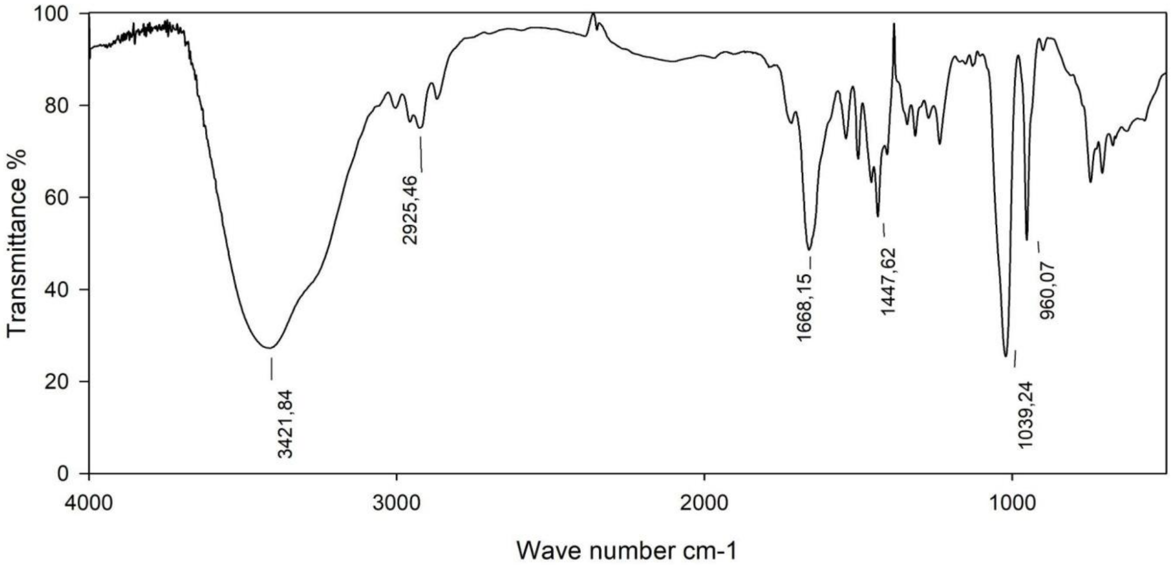

Fig. S12d FT-IR spectrum of compound **11d**

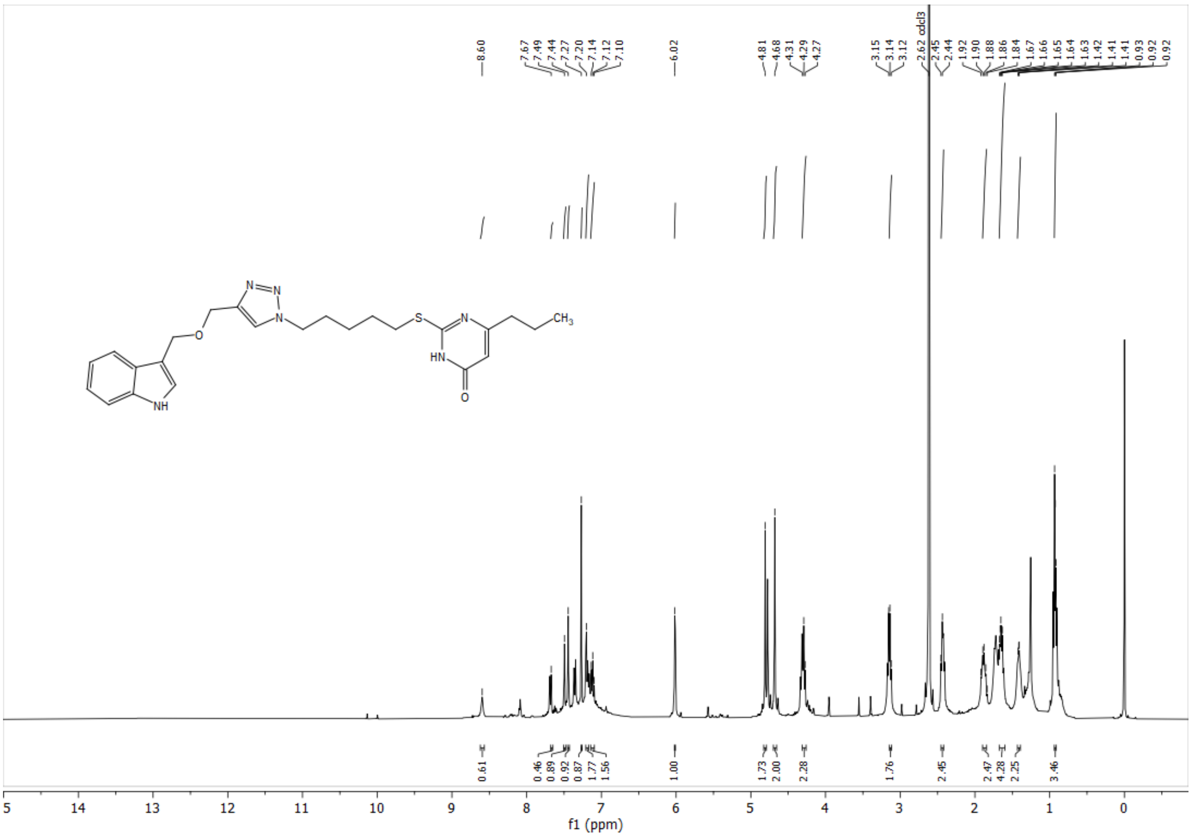

Fig. S13a ^{1}HNMR spectrum of compound **11e**

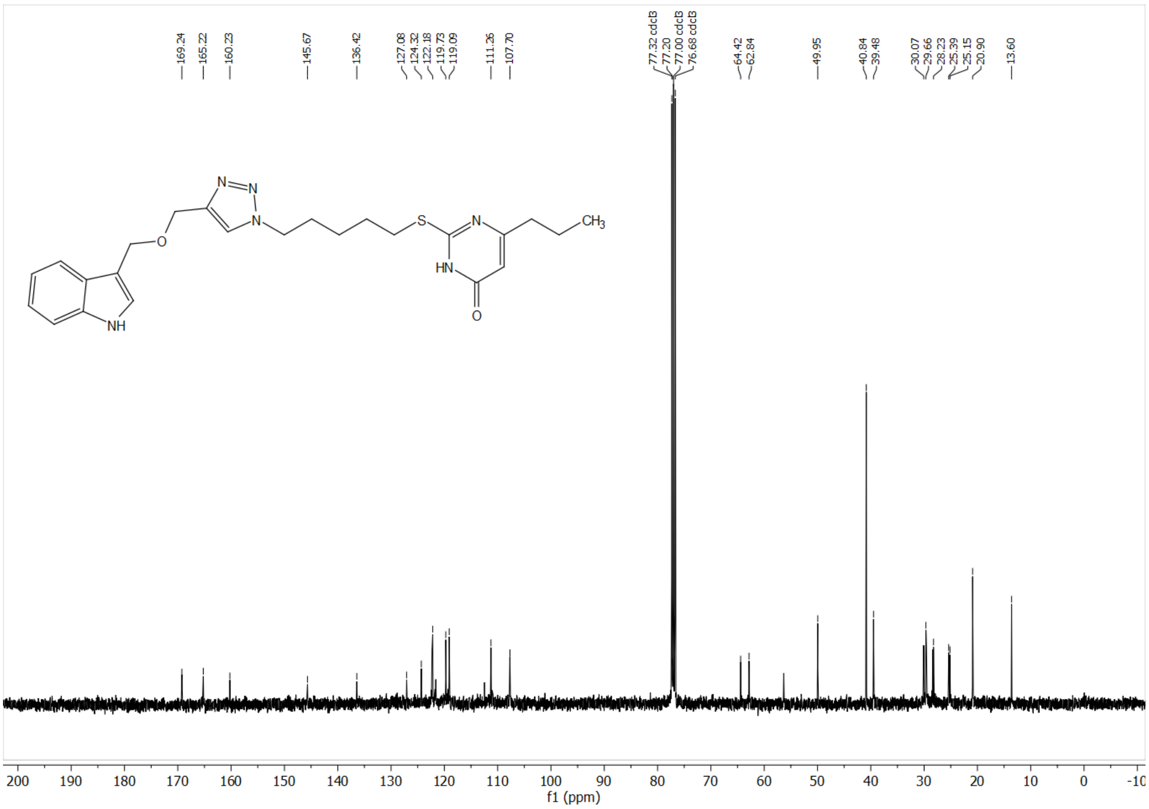

Fig. S13b ^{13}CNMR spectrum of compound **11e**

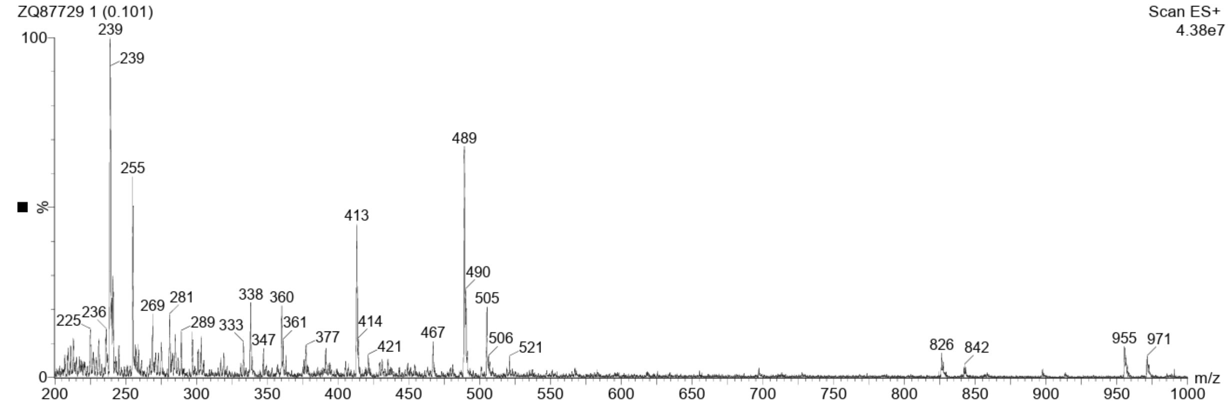

Fig. S13c ESI-MS spectrum of compound **11e**

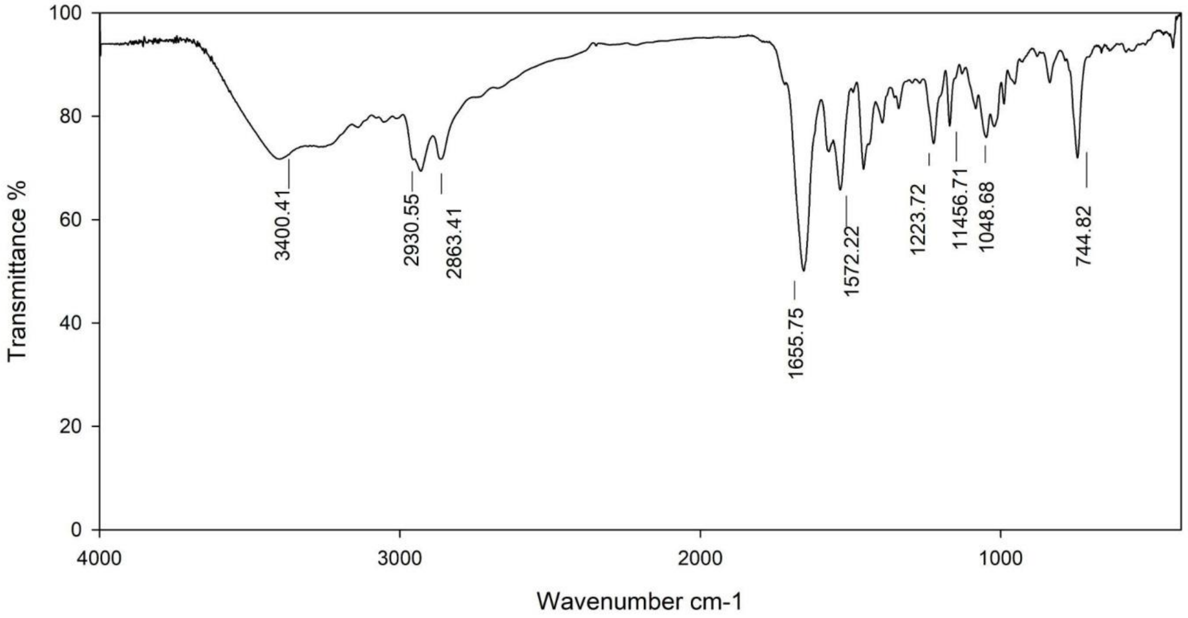

Fig. S13d FT-IR spectrum of compound **11e**

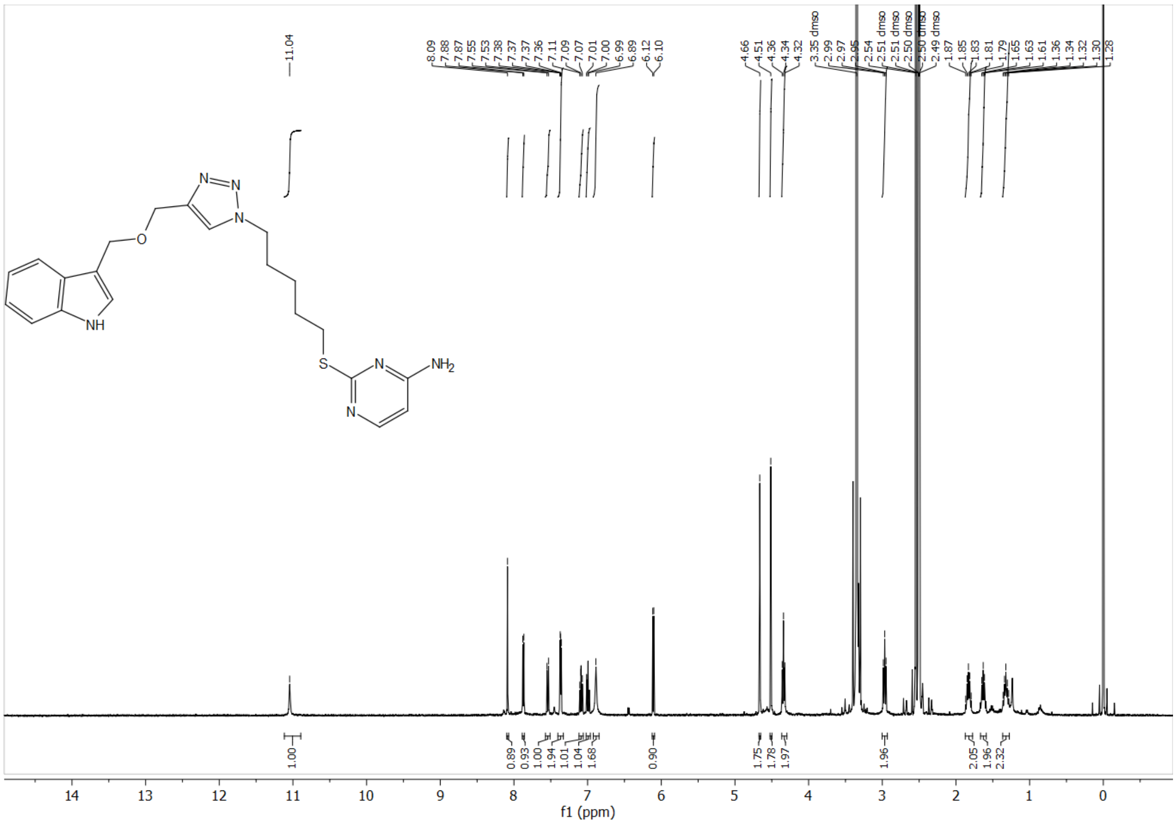

Fig. S14a ^{1}HNMR spectrum of compound **11f**

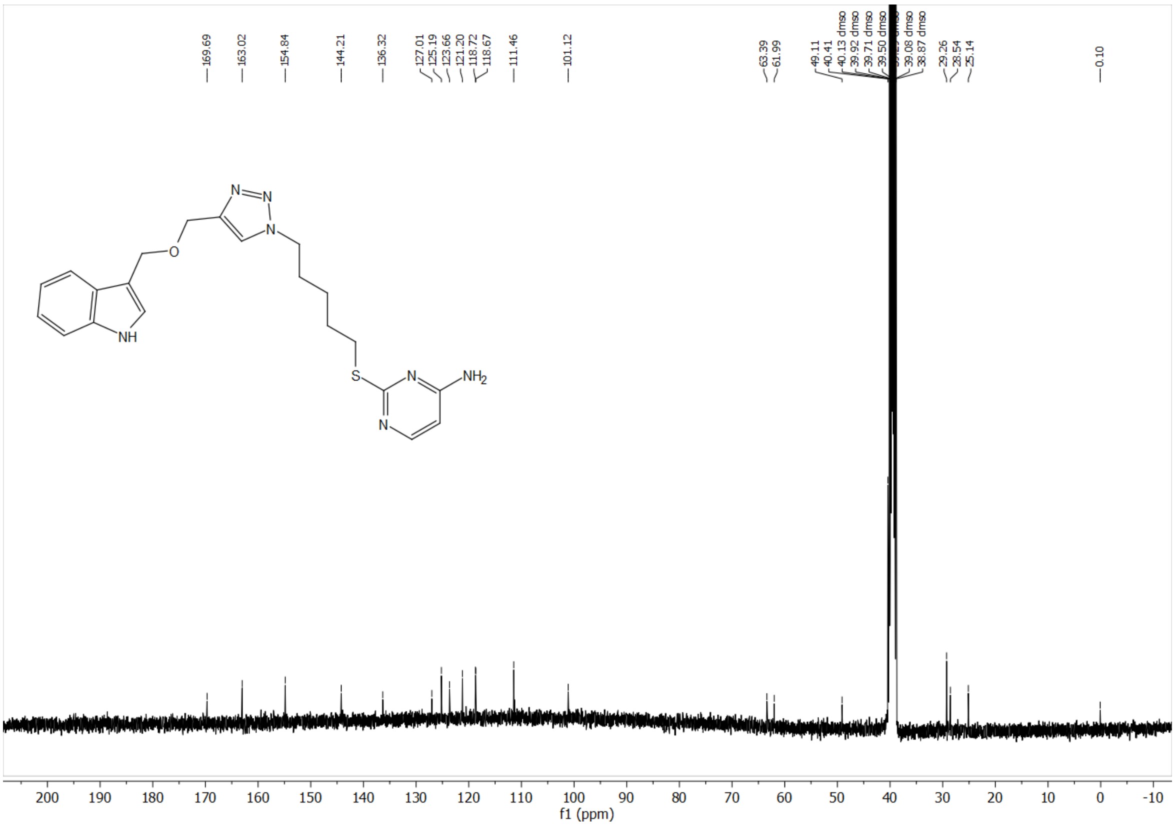

Fig. S14b ^{13}CNMR spectrum of compound **11f**

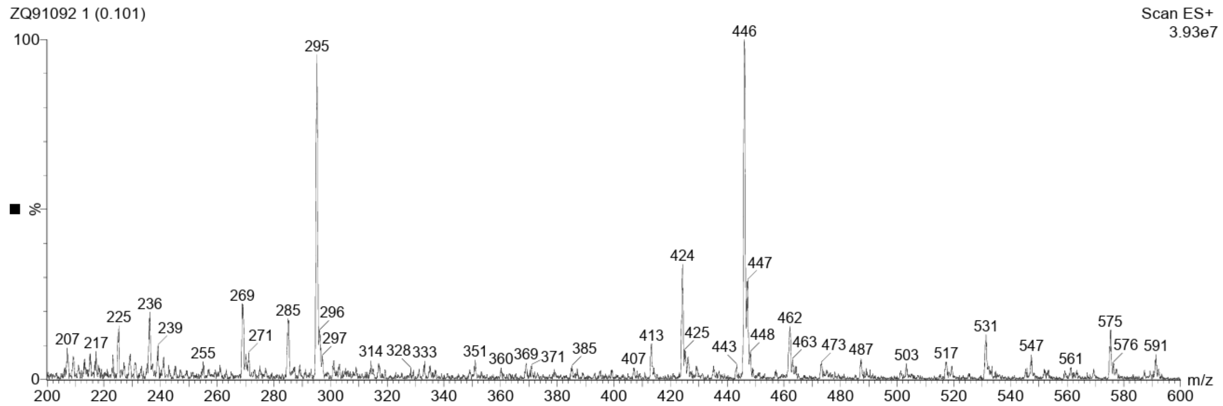

Fig. S14c ESI-MS spectrum of compound **11f**

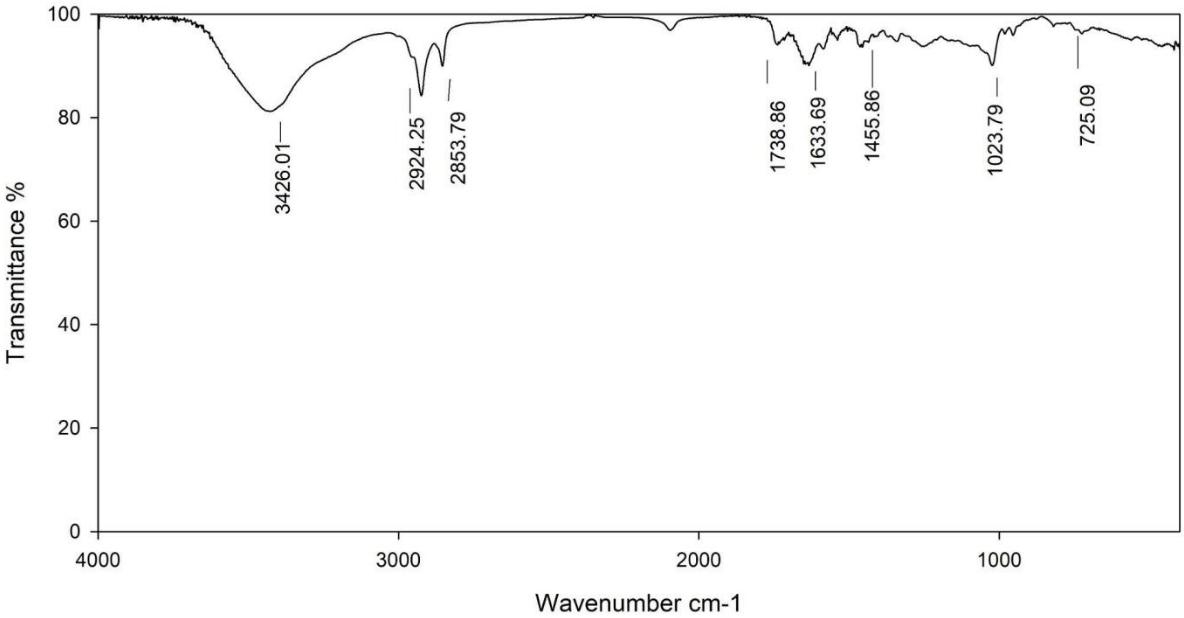

Fig. S14d FT-IR spectrum of compound **11f**

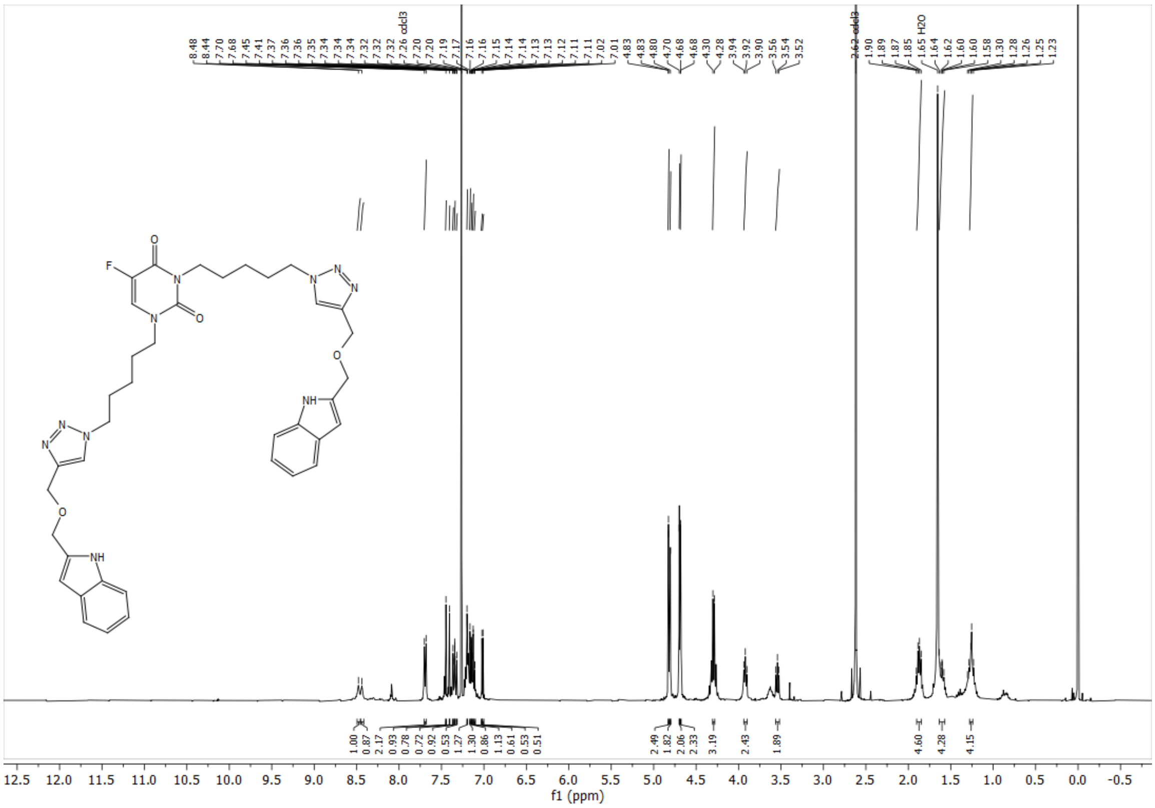

Fig. S15a ^{1}HNMR spectrum of compound **12**

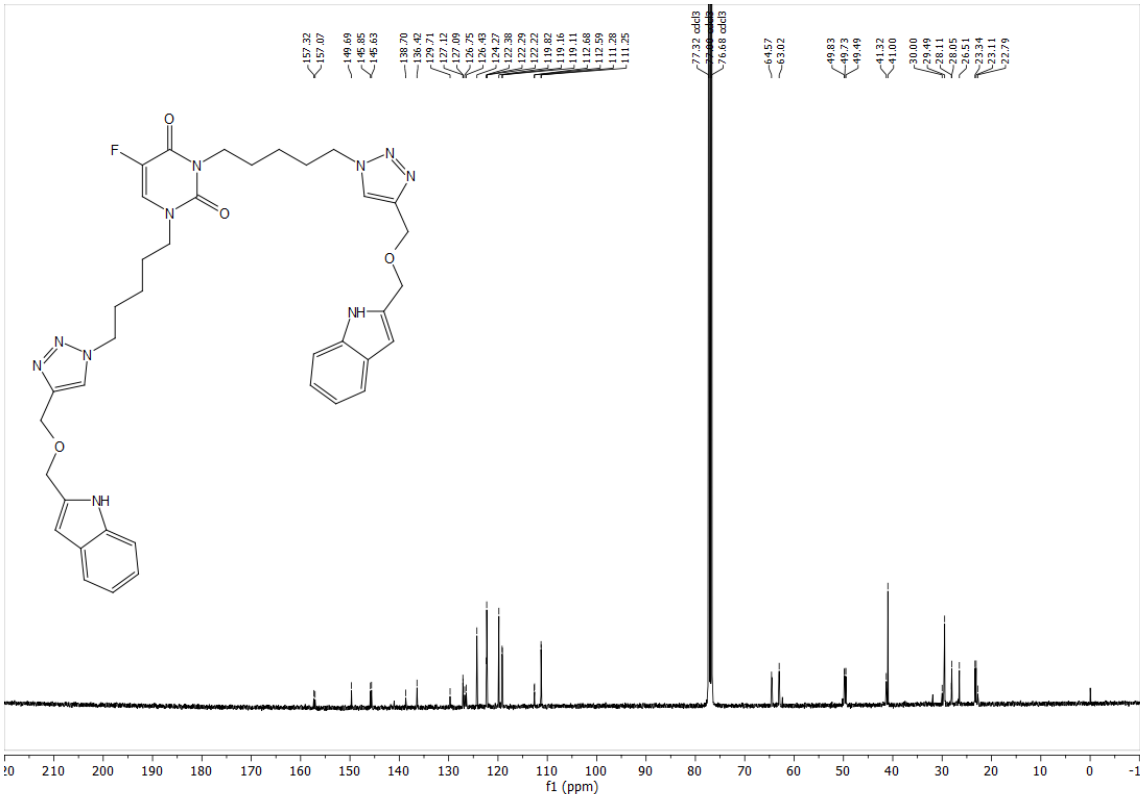

Fig. S15b ^{13}CNMR spectrum of compound **12**

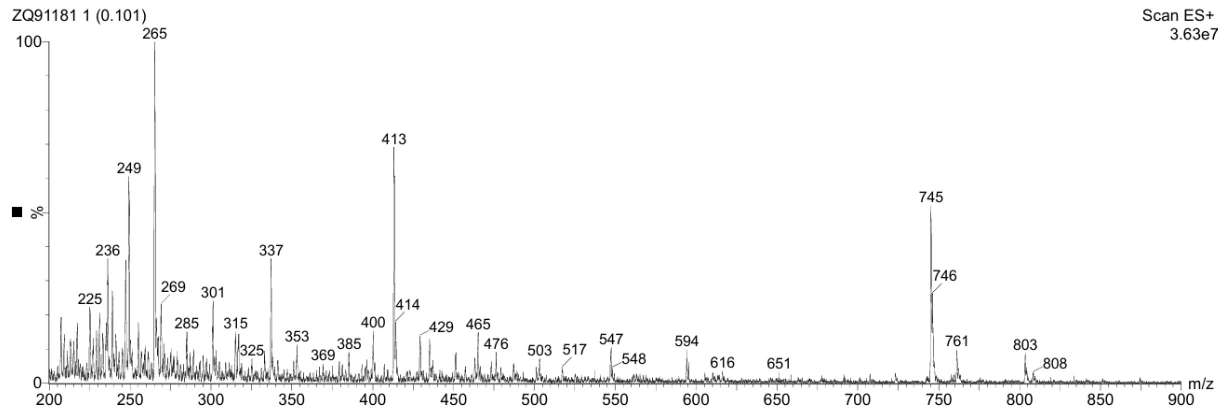

Fig. S15c ESI-MS spectrum of compound **12**

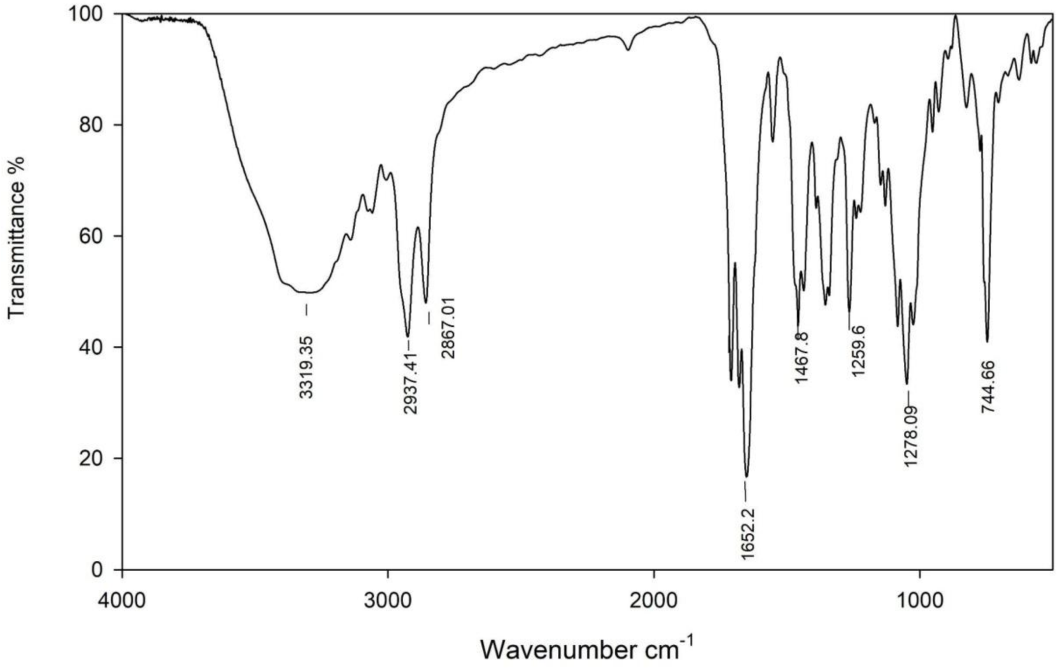

Fig. S15d FT-IR spectrum of compound **12**

Table. S1. Solubility in the water of all new compounds

Compound	logS	Solubility [mg/ml]	Solubility Class – logP scale
**9a**	-4.13	$1.2*10^{-1}$	Moderately Soluble
**9b**	-6.01	$4.4*10^{-3}$	Poorly Soluble
**9c**	-4.49	$6.04*10^{-2}$	Moderately Soluble
**9d**	-5,19	$1.85*10^{-2}$	Poorly Soluble
**9e**	-5.30	$1.40*10^{-2}$	Poorly Soluble
**9f**	-3.35	$3.75*10^{-1}$	Soluble
**10**	-3.40	$3.80*10^{-1}$	Moderately Soluble
**11a**	-5.16	$1.5*10^{-1}$	Poorly Soluble
**11b**	-7.03	$1.95*10^{-3}$	Poorly Soluble
**11c**	-5.52	$2.6*10^{-3}$	Moderately Soluble
**11d**	-6.22	$7.53*10^{-3}$	Poorly Soluble
	-6.32	$6.12*10^{-3}$	Poorly Soluble
**11f**	-4.37	$2.47*10^{-2}$	Moderately Soluble
**12**	-7.86	$7.85*10^{-4}$	Poorly Soluble

Table. S2. Antibacterial activity of all new compounds

Compound	*Micrococcus luteus*	*Bacillus subtilis*	*Escherichia coli*	*Pseudomonas fluorescens*
**9a**	3	0	4,5	2
**9b**	0	0	1	0
**9c**	4	2	5	0
**9d**	1	0	0	0
**9e**	0	0	3	1
**9f**	4	2	5	2,5
**10**	2,5	0	4	0
**11a**	1	2	3	0
**11b**	3,5	0	5	0
**11c**	2,5	0	4	0
**11d**	0	0	1	0
**11e**	0	0	5	0
**11f**	2,5	0	4	0
**12**	2	3	5	1,5
